# Estimation and inference on high-dimensional individualized treatment rule in observational data using split-and-pooled de-correlated score

**Published:** 2022

**Authors:** Muxuan Liang, Young-Geun Choi, Yang Ning, Maureen A Smith, Ying-Qi Zhao

**Affiliations:** Department of Biostatistics, University of Florida, Gainesville, Florida 32611, USA; Department of Statistics, Sookmyung Women’s University, Seoul 04310, Korea; Department of Statistics and Data Science, Cornell University, Ithaca, Newyork 14853, USA; Departments of Population Health and Family Medicine, University of Wisconsin-Madison, Madison, Wisconsin 53706, USA; Public Health Sciences Divisions, Fred Hutchinson Cancer Research Center, Seattle, Washington 98109, USA

**Keywords:** Individualized treatment rule, double-robustness, high-dimensional inference, semiparametric inference, precision medicine

## Abstract

With the increasing adoption of electronic health records, there is an increasing interest in developing individualized treatment rules, which recommend treatments according to patients’ characteristics, from large observational data. However, there is a lack of valid inference procedures for such rules developed from this type of data in the presence of high-dimensional covariates. In this work, we develop a penalized doubly robust method to estimate the optimal individualized treatment rule from high-dimensional data. We propose a split-and-pooled de-correlated score to construct hypothesis tests and confidence intervals. Our proposal adopts the data splitting to conquer the slow convergence rate of nuisance parameter estimations, such as non-parametric methods for outcome regression or propensity models. We establish the limiting distributions of the split-and-pooled de-correlated score test and the corresponding one-step estimator in high-dimensional setting. Simulation and real data analysis are conducted to demonstrate the superiority of the proposed method.

## 1. Introduction

An individualized treatment rule is a decision rule that maps the patient profiles X∈𝒳, a subspace of ${\mathbb{R}}^{p}$, into the intervention space A∈𝒜, where p is the number of the covariates and 𝒜 is the set of available interventions. Given an outcome of interest, the optimal individualized treatment rule maximizes the value function which is the mean outcome if it were applied to a target population. Understanding the driving factors of a data-driven treatment rule can help with identifying the source of the heterogeneous effects and with guiding practical applications of precision medicine.

The increasing adoption of electronic health records at healthcare centers has provided us unprecedented opportunities to understand the optimal individualized treatment rule through massive observational data. One of the difficulties in dealing with observational data is the high-dimensionality of the covariates. There have been various methods developed to estimate the optimal individualized treatment rule. For regression-based approaches, Q-learning methods (Watkins and Dayan, 1992; Chakraborty et al., 2010; Qian and Murphy, 2011; Laber et al., 2014a) pose a fully specified model assumption on the conditional mean of the outcomes given the covariates and treatments. Qian and Murphy (2011) approximates the conditional mean by a rich linear model, along with an $l_{1}$ penalty to accommodate high-dimensional data. A-learning methods (Murphy, 2003; Lu et al., 2013; Shi et al., 2016, 2018; Wu et al., 2021) pose a model assumption on the contrast function of the conditional means. With high-dimensional covariates, Shi et al. (2016, 2018) adopt penalized estimating equation or penalized regression with a linear contrast function. An alternative class of methods searches over a pre-specified class of individualized treatment rules to optimize an estimator of the mean outcome, usually called direct (Laber et al., 2014b), policy learning (Athey and Wager, 2017), value-search (Davidian et al., 2014) estimators, or C-learning (Zhang and Zhang, 2018). Especially, Zhang et al. (2012) adopts a doubly robust strategy to estimate the value under any treatment rules and directly optimize the estimated value. Their procedure can be applied to the observational data by plugging in a parametrically-estimated propensity score. Similarly, A-learning can also be extended to deal with observational data by plugging in a parametrically-estimated propensity score. Among these methods, Zhao et al. (2012) propose the outcome weighted learning approach based on an inverse probability weighted estimator of the value. Song et al. (2015) develop a variable selection method based on penalized outcome weighted learning for optimal individualized treatment selection.

Statistical inference for the optimal or estimated individualized treatment rule is particularly challenging in the presence of high-dimensional covariates. Confounding and selection bias presented in large observational data such as EHR data add one more layer of complexity. Liang et al. (2018b) propose a concordance-assisted learning algorithm in the presence of high-dimensional covariates. Nonetheless, they do not provide any inference procedures. Inference methods for A-learning approaches such as Song et al. (2017) and Jeng et al. (2018) are developed assuming the propensity score is known. Recently, Wu et al. (2021) provide an inference procedure for a high-dimensional single-index contrast function assuming a known propensity. Thus, their methods cannot be applied if data are collected from observational studies. Shi et al. (2018) derive the oracle inequalities of the proposed estimators for the parameters in a linear contrast function, but their work focuses on the selection consistency and has little discussion on the inference of the estimated rule. Their method depends on parametric assumptions on the propensity and outcome models, and thus may not be consistent when complex propensity or outcome models are expected. In practice, to avoid misspecification, flexible models may be adopted for the outcome regression or the propensity score. However, these models result in slow convergence rates for the nuisance parameters, and deteriorate the limiting distribution of the estimated decision rule. As such, it is important to propose an inference procedure for the estimated decision rule, which is valid under the high-dimensional setup and robust to flexible models for the nuisance parameters. Recent literature on the high-dimensional inference can assist with tackling this challenge. For example, van de Geer et al. (2014) propose a debiased Lasso approach for generalized linear models. Ning and Liu (2017) propose a de-correlated score test for low-dimensional parameters with the existence of the high-dimensional covariates, which is applicable for parametric models with correctly specified likelihoods. Dezeure et al. (2017) propose a bootstrap procedure for high-dimensional inference, but it is computationally intensive.

Another importance and related topic is the inference of the optimal value. The inference of the optimal value has been shown to be challenging at exceptional laws (non-regular case) where there exists a subgroup of patients for which treatment effect vanishes (Chakraborty et al., 2010; Laber et al., 2014c; Goldberg et al., 2014). To achieve the inference of the optimal value in low-dimensional setup, Chakraborty et al. (2014) propose an m-out of-n bootstrap to construct a confidence interval for the value. Luedtke and Van Der Laan (2016) propose an online one-step estimator which is the weighted average the values estimated on chunks of data increasing in size. Recently, Shi et al. (2020) use a subagging algorithm to aggregate value estimates obtained by repeated sample splittings. In both Luedtke and Van Der Laan (2016) and Shi et al. (2020), a single-split procedure is also discussed to facilitate the computation, though the resulting confidence interval might be wider. However, the value inference for high-dimensional setup is lacking.

In this work, we propose a novel penalized doubly robust approach, termed as penalized efficient augmentation and relaxation learning, to estimate the optimal individualized treatment rule in observational studies with high-dimensional covariates. We construct the decision rules by optimizing a convex relaxation of the augmented inverse probability weighted estimator of the value with penalties, which generalizes the method proposed in Zhao et al. (2019) to high-dimensional setup. The proposed procedure involves estimation of the conditional means of the outcomes and the propensity scores as nuisance parameters. As long as one of the nuisance models is correctly specified, we can consistently estimate the optimal individualized treatment rules under certain conditions. Furthermore, we propose a split-and-pooled de-correlated score test, which provides valid hypothesis testing and interval estimation procedures to identify the driving factors of the estimated decision rule. The proposed procedure generalizes the de-correlated score (Ning and Liu, 2017) to handle the potential slow convergence rates from the nuisance parameters estimation and to allow a general loss function. Sample-splitting is adopted to separate the estimation of the nuisance parameters from the construction of the de-correlated score, which is adopted in Chernozhukov et al. (2018) for inference on a low-dimensional parameter of interest in the presence of high-dimensional nuisance parameters. However, the inference on the estimated decision rule using the proposed approach requires a more sophisticated analysis due to the convex relaxation schemes. Theoretically, we show that the split-and-pooled de-correlated score is asymptotically normal even when the nuisance parameters are estimated non-parametrically with slow convergence rates. In addition, we use a single-split procedure to infer the value under the estimated decision rule.

## 2. Method

In this section, we propose the penalized efficient augmentation and relaxation learning and then introduce the proposed inference procedure.

### 2.1 Penalized Efficient Augmentation and Relaxation Learning

Let X be a p-dimensional random vector, which contains the baseline covariates capturing patient profiles. We assume that p can be much larger than the sample size n. Let A∈{−1, 1} be the treatment assignment, and Y∈ℝ be the observed outcome that higher values are preferred. Here, we adopt the framework of potential outcomes (Rubin, 1974, 2005). Denote the potential outcome under treatment a∈{−1, 1} as Y(a). Then the observed outcome is Y=Y(a)I{a=A}, where I{⋅} is the indicator function. An individualized treatment rule, denoted by D, is a mapping from the space of covariates $𝒳\subseteq {\mathbb{R}}^{p}$ to the space of treatments 𝒜={−1, 1}. With a slight abuse of notation, we write the observed outcome under this decision rule as $Y\left(D\right)=\sum_{a\in \left\{-1,1\right\}}Y\left(a\right)I\left\{a=D\left(X\right)\right\}$. The expectation of Y(D), V(D)=E(Y(D)), is called the value function which is the average of the outcomes over the population if the decision rule were to be adopted. In order to express the value in terms of the data generative model, we assume the following conditions: 1) the stable unit treatment value assumption (Imbens and Rubin, 2015); 2) the strong ignorability Y(−1),Y(1)⊥A∣X;3) Consistency Y=Y(A). The stable unit treatment value assumption assumes that the potential outcomes for a patient do not vary with the treatments assigned to other patients. It also implies that there are no different versions of the treatment. The strong ignorability condition means that there is no unmeasured confounding between the potential outcomes and the treatment assignment mechanism. The optimal individualized treatment rule is defined as $D_{\text{opt}}={\text{argmax}}_{D}\left\{V\left(D\right)\right\}$.

In this paper, due to the high-dimensional nature of the data we work with, we focus on deriving a linear decision rule of the form $D\left(x\right)=\text{sgn}\left(x^{⊤}\beta \right)$, where x∈𝒳 and the function sgn(t)=1 if t≥0; sgn(t)=−1 if t<0. To ensure the identifiability, we assume that the $k^{\text{*}}$-th coordinate of β, $\beta_{k^{\text{*}}}=1$, for some $k^{\text{*}}$. The choice of $k^{\text{*}}$ can be determined by the domain knowledge. Let π(a;x)=P(A=a∣X=x) and Q(a;x)=E(Y∣X=x,A=a) for a∈{−1, 1} and x∈𝒳. Define the weights

$${\hat{W}}_{a}=W_{a}\left(Y,X,A,\hat{\pi },\hat{Q}\right)=\frac{YI\left\{A=a\right\}}{\hat{\pi }\left(a;X\right)}-\frac{\left[I\left\{A=a\right\}-\hat{\pi }\left(a;X\right)\right]\hat{Q}\left(a;X\right)}{\hat{\pi }\left(a;X\right)}$$

for a∈{−1, 1}, where $\hat{\pi }\left(a;X\right)$ and $\hat{Q}\left(a;X\right)$ are the estimators of π(a;X) and Q(a;X) respectively. Under the conditions above, the augmented inverse probability weighted estimator of the value function is

$$\hat{V}\left(D\right)=E_{n}\left[{\hat{W}}_{1}I\left\{D\left(X\right)=1\right\}+{\hat{W}}_{-1}I\left\{D\left(X\right)=-1\right\}\right],$$

where $E_{n}\left[\cdot \right]$ denotes the empirical average. The estimator $\hat{V}\left(D\right)$ enjoys the double robustness property. Assume that $\hat{Q}\left(a;x\right)$ and $\hat{\pi }\left(a;x\right)$ converge in probability uniformly to some deterministic limits, denoted by $Q^{m}\left(a;x\right)$ and $\pi^{m}\left(a;x\right)$, respectively. $\hat{V}\left(D\right)$ converges to $V^{m}\left(D\right)$, where

$$V^{m}\left(D\right)=E\left[W_{1}^{m}I\left\{D\left(X\right)=1\right\}+W_{-1}^{m}I\left\{D\left(X\right)=-1\right\}\right].$$

Here, $W_{a}^{m}=W_{a}\left(Y,X,A,\pi^{m},Q^{m}\right)$ is the limit that ${\hat{W}}_{a}$ converges to,a=±1. As shown in Zhao et al. (2019), if either $\pi^{m}\left(a;x\right)=\pi \left(a;x\right)$ or $Q^{m}\left(a;x\right)=Q\left(a;x\right)$, but not necessarily both, then $V^{m}\left(D\right)=V\left(D\right)$.

To avoid negative ${\hat{W}}_{a}$, we consider its positive and negative parts separately and define ${\hat{W}}_{a,+}=\left|{\hat{W}}_{a}\right|1\left\{{\hat{W}}_{a}\geq 0\right\}$ and ${\hat{W}}_{a,-}=\left|{\hat{W}}_{a}\right|1\left\{{\hat{W}}_{a}\leq 0\right\}$. Maximizing $\hat{V}\left(D\right)$ is equivalent to minimizing

(1) $$E_{n}\left[\left({\hat{W}}_{1,+}+{\hat{W}}_{-1,-}\right)I\left\{D\left(X\right)\neq 1\right\}+\left({\hat{W}}_{1,-}+{\hat{W}}_{-1,+}\right)I\left\{D\left(X\right)\neq -1\right\}\right].$$

Directly optimizing (1) is infeasible due to the indicator functions in the objective function, especially with a large number of covariates. To avoid minimizing the indicator function, we replace the indicator function with a strictly convex surrogate loss. Due to the strict convexity, the minimizer of the surrogate loss is always unique. Thus, we can relax the constraint that $\beta_{k^{\text{*}}}=1$. Furthermore, we add a sparse penalty function, which enables us to eliminate the unimportant variables from the derived rule. We denote the weight encouraging A=1 as ${\hat{\Omega }}_{+}={\hat{W}}_{1,+}+{\hat{W}}_{-1,-}$ and the weight encouraging A=−1 as ${\hat{\Omega }}_{-}={\hat{W}}_{1,-}+{\hat{W}}_{-1,+}$. Our proposed estimator $\hat{\beta }$ is

(2) $$\hat{\beta }=\text{arg }\underset{\beta }{\text{min}}E_{n}\left[{\hat{\Omega }}_{+}\phi \left(X^{⊤}\beta \right)+{\hat{\Omega }}_{-}\phi \left(-X^{⊤}\beta \right)\right]+\lambda_{n}P\left(\beta \right),$$

where ϕ is a convex surrogate loss, P(β) is a sparse penalty function with respect to β, and $\lambda_{n}$ is a tuning parameter controlling the amount of penalization. In this paper, we focus on the $l_{1}$-lasso penalty $P\left(\beta \right)=∥\beta {∥}_{1}$. The framework allows a broad class of surrogate loss functions, such as logistic loss, $\phi \left(t\right)=\text{log}\left(1+e^{-t}\right)$, see Section 3 for the detailed technical conditions on ϕ. The estimated decision rule can be subsequently obtained as $\hat{D}\left(X\right)=\text{sgn}\left(X^{⊤}\hat{\beta }\right)$.

### 2.2 Split-and-pooled De-correlated Score Test

We define

$$l_{\phi }\left(\beta ;\Omega_{+}^{m},\Omega_{-}^{m}\right)=\Omega_{+}^{m}\phi \left(X^{⊤}\beta \right)+\Omega_{-}^{m}\phi \left(-X^{⊤}\beta \right)$$

and $\beta^{\text{*}}={\text{arg min}}_{\beta }E\left[l_{\phi }\left(\beta ;\Omega_{+}^{m},\Omega_{-}^{m}\right)\right]$, where $\Omega_{+}^{m}=W_{1,+}^{m}+W_{-1,-}^{m}$ and $\Omega_{-}^{m}=W_{1,-}^{m}+W_{-1,+}^{m}$. To simplify notations, we will suppress the superscript and write them as $\Omega_{+}$ and $\Omega_{-}$ instead. Let $X_{j}\in \mathbb{R}$ is the j-th covariate and $X_{-j}\in {\mathbb{R}}^{p-1}$ includes the remaining covariates. Likewise, let $\beta_{j}^{\text{*}}$ be the j-th coordinate of $\beta^{\text{*}}$ and $\beta_{-j}^{\text{*}}$ be a p−1 dimensional sub-vector of $\beta^{\text{*}}$ without $\beta_{j}^{\text{*}}$. Without loss of generality, suppose that $\beta_{j}^{\text{*}}$ is of interest. The statistical inference problem can be formulated as testing the null hypothesis $H_{0}:\beta_{j}^{\text{*}}=0$ versus $H_{1}:\;\beta_{j}^{\text{*}}\neq 0$, or constructing confidence intervals for $\beta_{j}^{\text{*}}$. The proposed method can be easily generalized to test any low-dimensional projection of $\beta^{\text{*}}$.

Before we propose our inference procedure for $\beta^{\text{*}}$, we introduce a lemma to show that under certain conditions, our inference procedure for $\beta^{\text{*}}$ can provide information on $D_{\text{opt}}\left(x\right)$. In Lemma 1, we assume that $D_{\text{opt}}\left(x\right)=\text{sgn}\left(x^{⊤}\beta^{\text{opt}}\right)$, which also indicates $D_{\text{opt}}\left(x\right)=\text{sgn}\left(cx^{⊤}\beta^{\text{opt}}\right)$ for any c>0. To avoid $\beta^{\text{opt}}=0$ and identifiability issue, we restrict inference to regimes in which $\beta_{k^{\text{*}}}^{\text{opt}}=1$. This implies that we would not infer $\beta_{k^{\text{*}}}^{\text{opt}}$ through $\beta_{k^{\text{*}}}^{\text{*}}$. In general, the contrast function Q(1;x)−Q(−1;x) could be a complex function of x, but in many situations, the optimal rule $D_{\text{opt}}\left(x\right)$ may only depend on a linear function of x (Xu et al., 2015). In Lemma 1, we provide sufficient conditions that $\beta^{\text{*}}$ satisfies $D_{\text{opt}}\left(X\right)=\text{sgn}\left(X^{\prime }\beta^{\text{opt}}\right)=\text{sgn}\left(X^{⊤}\beta^{\text{*}}\right)$. In this case, the results on the sparsity pattern of $\beta^{\text{*}}$ can be extended to inferring $\beta^{\text{opt}}$.

Define two subspaces depending on β,

$$\Delta_{\phi }\left(\beta \right)=\left\{f\left(X\right)\in L_{2}:\text{cov}\left[f\left(X\right),\left\{\phi \left(X^{⊤}\beta \right)-\phi \left(-X^{⊤}\beta \right)\right\}|X^{⊤}\beta^{\text{opt}}\right]\geq 0\right\},\;S_{\phi }\left(\beta \right)=\left\{f\left(X\right)\in L_{2}:\text{cov}\left[f\left(X\right),\left\{\phi \left(X^{⊤}\beta \right)+\phi \left(-X^{⊤}\beta \right)\right\}|X^{⊤}\beta^{\text{opt}}\right]\geq 0\right\}.$$

**Lemma 1** *If the* $D_{\text{opt}}\left(X\right)$ *has a linear form, and* $Q^{m}=Q$ or $\pi^{m}=\pi$ *in* $\Omega_{+}$ and $\Omega_{-}$, *then* $D_{\text{opt}}\left(X\right)=\text{sgn}\left(X^{⊤}\beta^{\text{*}}\right)$ *if the following conditions are satisfied*: (a) The contrast function $Q\left(1;X\right)-Q\left(-1;X\right)\in \Delta_{\phi }\left(\beta^{\text{*}}\right)$, *and the main effect* $E\left(Y\left(1\right)+Y\left(-1\right)|X\right)\in S_{\phi }\left(\beta^{\text{*}}\right);\left(b\right)$ *there exists a p-dimensional vector P such that $E\left(X|X^{⊤}\beta^{\text{opt}}\right)=PX^{⊤}\beta^{\text{opt}}$*.

The subspaces $\Delta_{\phi }\left(\beta \right)$ and $S_{\phi }\left(\beta \right)$ enjoy the following properties: (i) Any measurable function of $X^{⊤}\beta^{\text{opt}}$ belongs to $\Delta_{\phi }\left(\beta \right)\cap S_{\phi }\left(\beta \right),\forall \beta$; (ii) Suppose that a function $g\left(X\right)\in \Delta_{\phi }\left(\beta \right)$ (or $S_{\phi }\left(\beta \right)$), then the function $h\left(X^{⊤}\beta^{\text{opt}}\right)g\left(X\right)\in \Delta_{\phi }\left(\beta \right)$ (or $S_{\phi }\left(\beta \right)$), where h(⋅) is an arbitrary measurable function. Thus, if $E\left(Y_{1}|X\right)$ and $E\left(Y_{-1}|X\right)$ only depend on $X^{⊤}\beta^{\text{opt}}$, Condition (a) is easily satisfied. We provide examples in the Appendix B to further show that Condition (a) is satisfied by a large class of models, including data generative models that are dense and not single index models (see Example 2 in Appendix B). In particular, although method proposed in Wu et al. (2021) can deal with general single index models, it has more restriction on the sparsity level of the contrast function than our requirement.

Condition (b) on the design matrix X is common in the dimension reduction literature (Li, 1991; Zhu et al., 2006; Lin et al., 2018, 2019). It is satisfied if the distribution of X is elliptically symmetric. Li and Duan (1989); Duan and Li (1991) provide a thorough discussion on this condition in regression methods which aims to estimate a single index with an arbitrary and unknown link function. More specifically, they provide a bias bound when the elliptical symmetry is violated and show that the asymptotic bias is small when the elliptical symmetry is nearly satisfied. Further, Hall and Li (1993) shows that when the dimension of X is large, for most directions $\beta^{\text{opt}}$ even the most nonlinear regression is still nearly linear. In addition, empirical studies by Brillinger and others suggest that quite often the bias may be negligible even for a moderate violation of condition (b) (Brillinger, 2012; Li and Duan, 1989).

**Remark 2** *Alternatively, instead of assuming the conditions in Lemma 1, the desired relationship* $D_{\text{opt}}\left(X\right)=\text{sgn}\left(X^{⊤}\beta^{\text{*}}\right)$ *may still hold under some parametric assumptions on $E\left(Y_{1}|X\right)$ and $E\left(Y_{-1}|X\right)$*. *For example, if the outcomes are non-negative and the following conditions are satisfied*

(3) $$\text{log}\left\{E\left(Y_{1}|X\right)/E\left(Y_{-1}|X\right)\right\}=X^{⊤}\beta^{\text{opt}},$$

we still have $D_{\text{opt}}\left(X\right)=\text{sgn}\left(X^{⊤}\beta^{\text{*}}\right)$. *Condition* (3) *poses a parametric assumption on $E\left(Y_{1}|X\right)/E\left(Y_{-1}|X\right)$ (see* Appendix B *for the details). This ratio measures the relative change of the potential outcomes. Under Condition* (3), hypothesis testing of $\beta^{\text{*}}$ is equivalent to testing for the driving factors of the $D_{\text{opt}}$. *Furthermore, the interval estimation of* $\beta^{\text{*}}$ *can be interpreted through the specified model assumption in* (3).

Next, we introduce our proposed inference procedure. Suppose that $\Omega_{+}$ and $\Omega_{-}$ are known, then the estimator $\hat{\beta }$ is obtained by minimizing the empirical loss

$$E_{n}\left[l_{\phi }\left(\beta ;\Omega_{+},\Omega_{-}\right)\right]+\lambda_{n}P\left(\beta \right).$$

Let $\nabla l_{\phi }\left(\beta ;\Omega_{+},\Omega_{-}\right)=\Omega_{+}\phi^{\prime }\left(X^{⊤}\beta \right)-\Omega_{-}\phi^{\prime }\left(-X^{⊤}\beta \right)$. For $j\neq k^{\text{*}}$, the score function of $\beta_{j}$ is ${\text{E}}_{n}\left[\nabla l_{\phi }\left(\beta ;\Omega_{+},\Omega_{-}\right)X_{j}\right]$. Let ${\hat{\beta }}_{\text{null}\left(\text{j}\right)}$ be a vector that equals to $\hat{\beta }$ with the j-th coordinate replaced by 0. In the low-dimensional setting where p is fixed, the score function with ${\hat{\beta }}_{\text{null}\left(\text{j}\right)}$, $E_{n}\left[\nabla l_{\phi }\left({\hat{\beta }}_{\text{null(j)}};\Omega_{+},\Omega_{-}\right)X_{j}\right]$, is asymptotically normal. Nevertheless, in a high-dimensional setting, the asymptotic normality of the score function $E_{n}\left[\nabla l_{\phi }\left({\hat{\beta }}_{\text{null}\left(\text{j}\right)};\Omega_{+},\Omega_{-}\right)X_{j}\right]$ is deteriorated by the high-dimensionality of ${\hat{\beta }}_{-j}$. Following Ning and Liu (2017), we use the semiparametric theory to de-couple the estimation error of ${\hat{\beta }}_{-j}$ with the score function of $\beta_{j}$. A de-correlated score function is defined as $E_{n}\left[\nabla l_{\phi }\left({\hat{\beta }}_{\text{null(j)}};\Omega_{+},\Omega_{-}\right)\left(X_{j}-X_{-j}^{⊤}w_{j}^{\text{*}}\right)\right]$, where $w_{j}^{\text{*}}={\left(I_{-j,-j}^{\text{*}}\right)}^{-1}I_{-j,j}^{\text{*}}$ is chosen to reduce the uncertainty of the score function due to the estimation error of ${\hat{\beta }}_{-j}$. Denote $\nabla^{2}l_{\phi }\left(\beta ;\Omega_{+},\Omega_{-}\right)=\Omega_{+}\phi^{\prime \prime }\left(X^{⊤}\beta \right)+\Omega_{-}\phi^{\prime \prime }\left(-X^{⊤}\beta \right)$. The $I_{-j,-j}^{\text{*}}$ and $I_{-j,j}^{\text{*}}$ are the corresponding partitions of $I^{\text{*}}=E\left[\nabla^{2}l_{\phi }\left(\beta^{\text{*}};\Omega_{+},\Omega_{-}\right)XX^{⊤}\right]$.

Under the null hypothesis, this de-correlated score function follows

$$n^{1/2}E_{n}\left[\nabla l_{\phi }\left({\hat{\beta }}_{\text{null}\left(\text{j}\right)};\Omega_{+},\Omega_{-}\right)\left(X_{j}-X_{-j}^{⊤}w_{j}^{\text{*}}\right)\right]\to N\left(0,{\left(\nu_{j}^{\text{*}}\right)}^{⊤}I\nu_{j}^{\text{*}}\right),$$

where $\nu_{j}^{\text{*}}$ is a vector whose j-th coordinate is 1 and other coordinates equal to $-w_{j}^{\text{*}}$. We propose to estimate the nuisance parameter $w_{j}^{\text{*}}$ via

$$\underset{w}{\text{min}}E_{n}\left[\nabla^{2}l_{\phi }\left(\hat{\beta };\Omega_{+},\Omega_{-}\right){\left(X_{j}-X_{-j}^{⊤}w\right)}^{2}\right]+{\tilde{\lambda }}_{n}∥w{∥}_{1},$$

where ${\tilde{\lambda }}_{n}$ is a tuning parameter. Denote the estimator for $w_{j}^{\text{*}}$ as ${\hat{w}}_{j}$. A valid test for $H_{0}:\beta_{j}^{\text{*}}=0$ is constructed based on

(4) $$E_{n}\left[\nabla l_{\phi }\left({\hat{\beta }}_{\text{null}\left(\text{j}\right)};\Omega_{+},\Omega_{-}\right)\left(X_{j}-X_{-j}^{⊤}{\hat{w}}_{j}\right)\right].$$

The nuisance parameters, $\Omega_{+}$ and $\Omega_{-}$ are unknown in practice, and are estimated via modeling π and Q. To avoid misspecification, they can be estimated using flexible non-parametric or machine learning methods, which may lead to convergence rates slower than $n^{-1/2}$. To overcome the possible slow convergence rates of $\hat{\pi }$ and $\hat{Q}$, we propose a split-and-pooled de-correlated score, where we consider a sample split procedure in constructing the de-correlated score function (Chernozhukov et al., 2018).

Let $I_{1},\ldots ,I_{K}$ be a random partition of the observed data with approximately equal sizes, where K≥2 is a fixed pre-specified integer. We assume that $\left\lfloor n/K\right\rfloor \leq \left|I_{k}\right|\leq \left\lfloor n/K\right\rfloor +1$, for all k=1,…,K. Let $E_{n}^{\left(k\right)}\left[\cdot \right]$ denote the expectation defined by the data in $I_{k}$. For each k∈{1,…,K}, we repeat the following procedure. First, we obtain ${\hat{\pi }}_{\left(-k\right)}$ and ${\hat{Q}}_{\left(-k\right)}$ using the data excluding $I_{k}$. In the presence of high-dimensional covariates, we can use generalized linear model with penalties (van de Geer, 2008) or kernel regression after a model-free variable screening (Li et al., 2012; Cui et al., 2015) for estimating π and Q. A data-split estimator ${\hat{\beta }}^{\left(k\right)}$ is obtained by

(5) $${\hat{\beta }}^{\left(k\right)}=\text{arg }\underset{\beta }{\text{min}}\;E_{n}^{\left(k\right)}\left[l_{\phi }\left(\beta ;{\hat{\Omega }}_{+}^{\left(-k\right)},{\hat{\Omega }}_{-}^{\left(-k\right)}\right)\right]+\lambda_{n,k}{\left\|\beta \right\|}_{1},$$

where ${\hat{\Omega }}_{+}^{\left(-k\right)}$ and ${\hat{\Omega }}_{-}^{\left(-k\right)}$ are computed with ${\hat{\pi }}_{\left(-k\right)}$ and ${\hat{Q}}_{\left(-k\right)}$ plugged in, and $\lambda_{n,k}$ is a tuning parameter. Then, we estimate $w_{j}^{\text{*}}$ by

(6) $${\hat{w}}_{j}^{\left(k\right)}=\text{arg }\underset{w}{\text{min}}\;E_{n}^{\left(k\right)}\left[\nabla^{2}l_{\phi }\left({\hat{\beta }}^{\left(k\right)};{\hat{\Omega }}_{+}^{\left(-k\right)},{\hat{\Omega }}_{-}^{\left(-k\right)}\right){\left(X_{j}-X_{-j}^{⊤}w\right)}^{2}\right]+{\tilde{\lambda }}_{n,k}{\left\|w\right\|}_{1},$$

where ${\tilde{\lambda }}_{n,k}$ is a tuning parameter. Let $\left({\hat{\beta }}_{\text{null}\left(\text{j}\right)}^{\left(k\right)}\right)$ be a vector that equals ${\hat{\beta }}^{\left(k\right)}$ except its j-th coordinate replaced by 0. Finally, we construct the data-split de-correlated score test statistic $S_{j}^{\left(k\right)}\left({\hat{\beta }}_{\text{null}\left(\text{j}\right)}^{\left(k\right)},{\hat{w}}_{j}^{\left(k\right)}\right)$ as

(7) $$S_{j}^{\left(k\right)}\left({\hat{\beta }}_{\text{null}\left(\text{j}\right)}^{\left(k\right)},{\hat{w}}_{j}^{\left(k\right)}\right)=E_{n}^{\left(k\right)}\left[\nabla l_{\phi }\left({\hat{\beta }}_{\text{null}\left(\text{j}\right)}^{\left(k\right)};{\hat{\Omega }}_{+}^{\left(-k\right)},{\hat{\Omega }}_{-}^{\left(-k\right)}\right)\left(X_{j}-X_{-j}^{⊤}{\hat{w}}_{j}^{\left(k\right)}\right)\right].$$

Combining K data-split estimators, we obtain the pooled estimator $\hat{\beta }=K^{-1}\sum_{k=1}^{K}{\hat{\beta }}^{\left(k\right)}$. Likewise, the pooled de-correlated score test statistic is $S_{j}=K^{-1}\sum_{k=1}^{K}S_{j}^{\left(k\right)}\left({\hat{\beta }}_{\text{null}\left(\text{j}\right)}^{\left(k\right)},{\hat{w}}_{j}^{\left(k\right)}\right)$.

As shown in Theorem 4, under null hypothesis $\left(\beta_{j}^{\text{*}}=0\right)$, we have

$$n^{1/2}S_{j}\to N\left(0,{\left(\nu_{j}^{\text{*}}\right)}^{⊤}I\nu_{j}^{\text{*}}\right),$$

uniformly holds over all j‘s. The detailed algorithm is provided in Algorithm 1. In this algorithm, for a fixed $1\leq k\leq K,{\hat{\pi }}_{\left(-k\right)}$ and ${\hat{Q}}_{\left(-k\right)}$ are trained on a subset of samples of size n(K−1)/K.

### 2.3 Confidence Intervals

We use the data-split de-correlated score to construct a valid confidence interval of $\beta_{j}^{\text{*}}$. This is motivated from the fact that the data-split de-correlated score $S_{j}^{\left(k\right)}\left(\beta ,{\hat{w}}_{j}^{\left(k\right)}\right)$ is also an unbiased estimating equation for $\beta_{j}^{\text{*}}$ when fixing $\beta_{-j}=\beta_{-j}^{\text{*}}$. However, directly solving this estimating equation has several drawbacks, such as the existence of multiple roots or illposed Hessian (Chapter 5 in van der Vaart (2000)). Ning and Liu (2017) proposed a one-step estimator, which solved a first order approximation of the de-correlated score. Following their procedure, we construct the data-split one-step estimator, ${\tilde{\beta }}_{j}^{\left(k\right)}$, as the solution to,

$$S_{j}^{\left(k\right)}\left({\hat{\beta }}^{\left(k\right)},{\hat{w}}_{j}^{\left(k\right)}\right)+E_{n}^{\left(k\right)}\left[\nabla^{2}l_{\phi }\left({\hat{\beta }}^{\left(k\right)};{\hat{\Omega }}_{+}^{\left(-k\right)},{\hat{\Omega }}_{-}^{\left(-k\right)}\right)X_{j}\left(X_{j}-X_{-j}^{⊤}{\hat{w}}_{j}^{\left(k\right)}\right)\right]\left(\beta_{j}-{\hat{\beta }}_{j}^{\left(k\right)}\right)=0.$$

Hence, we have that ${\tilde{\beta }}_{j}^{\left(k\right)}={\hat{\beta }}_{j}^{\left(k\right)}-S_{j}^{\left(k\right)}\left({\hat{\beta }}^{\left(k\right)},{\hat{w}}_{j}^{\left(k\right)}\right)/{\hat{I}}_{j|-j}^{\left(k\right)}$, where

$${\hat{I}}_{j|-j}^{\left(k\right)}=E_{n}^{\left(k\right)}\left[\nabla^{2}l_{\phi }\left({\hat{\beta }}^{\left(k\right)};{\hat{\Omega }}_{+}^{\left(-k\right)},{\hat{\Omega }}_{-}^{\left(-k\right)}\right)X_{j}\left(X_{j}-X_{-j}^{⊤}{\hat{w}}_{j}^{\left(k\right)}\right)\right].$$

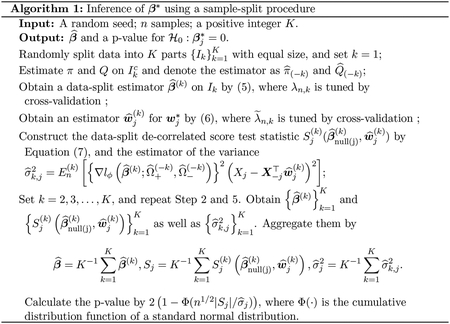


Finally, the pooled one-step estimator is the aggregation of these data-split one-step estimators following ${\tilde{\beta }}_{j}=K^{-1}\sum_{k=1}^{K}{\tilde{\beta }}_{j}^{\left(k\right)}$. In Section 3, we will show the asymptotic normality of the pooled one-step estimator ${\tilde{\beta }}_{j}$, which provides a valid confidence interval for $\beta_{j}^{\text{*}}$. The algorithm for constructing confidence intervals is presented in Algorithm 2.


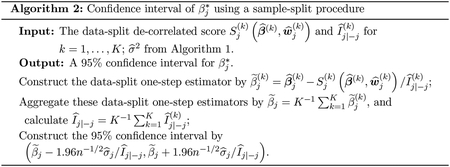


### 2.4 Inference of the Value

We adopt an analogy of the single-split procedure (Luedtke and Van Der Laan, 2016; Shi et al., 2020) to infer the value under $D^{\text{*}}\left(X\right),V\left(D^{\text{*}}\right)$, where $D^{\text{*}}\left(X\right)=\text{sgn}\left(X^{⊤}\beta^{\text{*}}\right)$. The single-split procedure splits the entire data set into two parts. We use one part for training and nuisance parameter fitting, and conduct inference on the other part. When $\beta^{\text{*}}\propto \beta_{\text{opt}}$, i.e., there is a constant c>0 such that $\beta^{\text{*}}=c\beta_{\text{opt}}$, our procedure provides a valid inference procedure for the optimal value. The detailed procedure for inference of the value is presented in Algorithm 3.

## 3. Theoretical Properties

We assume the following conditions.

(C1) Each covariate $X_{j}$‘s is sub-Gaussian with common proxy $\sigma_{x},{\left\|\beta^{\text{*}}\right\|}_{1}\leq R$, and ${\text{max}}_{j}\left\{{\left\|w_{j}^{\text{*}}\right\|}_{1}\right\}\leq R;{\text{sup}}_{x\in 𝒳}\left|Q\left(a;X\right)\right|$ is bounded, and the conditional distribution of Y(a)−Q(a;X) given X is sub-exponential, i.e., it is either bounded or satisfies that there exists some constants $M,\nu_{0}\in R$ such that

$$E\left[\text{exp}\left\{\left|Y\left(a\right)-Q\left(a;X\right)\right|/M\right\}-1-\left|Y\left(a\right)-Q\left(a;X\right)\right|/M|X\right]M^{2}\leq \nu_{0}/2$$

for both a=1 and a=−1.


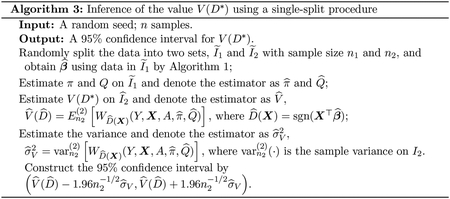


(C2) There exists some constants $0<\pi_{\text{min}}<\pi_{\text{max}}<1$ such that $\pi_{\text{min}}\leq \pi \left(a;X\right)\leq \pi_{\text{max}}$ with probability 1.

(C3) ϕ is convex, and $\phi^{\prime }$ is bounded with $\phi^{\prime }\left(0\right)<0$; for any $t\in \left[-\bar{c}-\epsilon ,\bar{c}+\epsilon \right]$ with some constant ϵ>0 and a sequence $t_{1}$ satisfying $\left|t_{1}-t\right|=o\left(1\right)$, it holds that $0<\phi^{\prime \prime }\left(t\right)\leq C$ and $\left|\phi^{\prime \prime }\left(t_{1}\right)-\phi^{\prime \prime }\left(t\right)\right|\leq C\left|t_{1}-t\right|\phi^{\prime \prime }\left(t\right)$ for some constant C>0.

(C4) The smallest eigenvalue of $E\left[\nabla^{2}l_{\phi }\left(\beta^{\text{*}};\Omega_{+},\Omega_{-}\right)XX^{⊤}\right]$ is larger than κ, where κ is a positive constant.

(C5) Suppose that for some $\alpha ,\beta >0,{\text{sup}}_{x}\left|\hat{\pi }\left(a;x\right)-\pi \left(a;x\right)\right|=O_{p}\left(n^{-\alpha }\right)$ and ${\text{sup}}_{x}\left|\hat{Q}\left(a;x\right)-Q\left(a;x\right)\right|=O_{p}\left(n^{-\beta }\right)$ for a=1 and −1, we require that $Rn^{-\alpha -\beta +1/2}=o\left(1\right)$. In addition, we require that

(8) $$R\text{ max}\left\{s^{\text{*}},s^{\prime }\right\}\text{log }n{\left(\text{log }p\right)}^{3/2}=o\left(n^{1/2}\right),$$

and

(9) $$\left(n^{-\alpha }+n^{-\beta }\right)s^{\text{*}}\sqrt{\text{log }p}\to 0,$$

where $s^{\text{*}}={\left\|\beta^{\text{*}}\right\|}_{0}$ and $s^{\prime }={\text{max}}_{j}{\left\|w_{j}^{\text{*}}\right\|}_{0}$.

Condition (C1) on the joint distribution of (X, A, Y) is weaker than the assumption in high-dimensional inference literature (van de Geer et al., 2014; Ning and Liu, 2017). Instead of sub-gaussian design, they only consider that the design is uniformly bounded. We also assume that Y(a)−Q(a;X) is sub-exponential or bounded. This condition enables a faster convergence rate of high-dimensional empirical processes involving the estimation errors of $\hat{\pi }$ and $\hat{Q}$. Under this condition, if ${\text{sup}}_{X}\left|\hat{Q}\left(a;X\right)-Q\left(a;X\right)\right|=o_{p}\left(1\right)$, we have

$${\left\|E_{n}\left[\left\{Y\left(a\right)-Q\left(a;X\right)\right\}\left\{Q\left(a;X\right)-\hat{Q}\left(a;X\right)\right\}X\right]\right\|}_{\infty }=o_{p}\left({\left(\text{log }p/n\right)}^{1/2}\right).$$

Condition (C2) prevents the extreme values in the true propensities. Condition (C3) requires that the surrogate loss ϕ has bounded first-order and second-order derivatives. The logistic loss satisfies these conditions. Condition (C4) is standard in high-dimensional inference literature (Ning and Liu, 2017; van de Geer et al., 2014; Dezeure et al., 2017). Condition (C5) is imposed for Algorithm 1. We assume that it holds on each split data set. To simplify the notation, we do not distinguish $\hat{\pi }$ and $\hat{Q}$ with ${\hat{\pi }}_{\left(-k\right)}$ and ${\hat{Q}}_{\left(-k\right)}$ for a fixed k. First it requires that both $\hat{\pi }$ and $\hat{Q}$ are consistent and the convergence rates satisfy $Rn^{-\alpha -\beta +1/2}=o\left(1\right)$. This can be attained if either the convergence rate of $\hat{\pi }$ or $\hat{Q}$ is sufficiently fast. For example, assuming R=O(1), if π is estimated by a regression spline estimator and is known to be $p_{\pi }$-dimensional (low dimension) by design, we have ${\text{sup}}_{X}\left|\hat{\pi }\left(a;X\right)-\pi \left(a;X\right)\right|=O_{p}\left(n^{-1/3}\right)$, where π is assumed to belong to the Hölder class with a smoothness parameter greater than $5p_{\pi }$ (Newey, 1997). Then $n^{-\alpha -\beta }\ll n^{-1/2}$ is satisfied when $n^{-\beta }\ll n^{-1/6}$. Second, formula (8) in Condition (C5) requires that the number of nonzero entries of $\beta^{\text{*}}$ and $w_{j}^{\text{*}}$ is smaller than the order of $n^{1/2}/{\left(\text{log }p\right)}^{3/2}$, which is slightly more restrictive than the conditions in the high-dimensional inference literature (van de Geer et al., 2014; Ning and Liu, 2017) due to the sub-gaussian design. Finally, formula (9) of Condition (C5) indicates the convergence rates of the nuisance parameter estimations cannot be too slow if $s^{\text{*}}$ and p increase fast with the sample size n.

**Theorem 3** *Assume that Conditions (C1)*–*(C5) hold. By choosing $\lambda_{n,k}≍{\left(\text{log}p/n\right)}^{1/2}$*, we have ${\left\|\hat{\beta }-\beta^{\text{*}}\right\|}_{1}=O_{p}\left(s^{\text{*}}{\left(\text{log }p/n\right)}^{1/2}\right)$.

Theorem 3 assumes that both the outcome and propensity score models are correctly specified, $Q^{m}=Q$ and $\pi^{m}=\pi$ (implied by Condition (C5)). Nonetheless, our proposed estimator enjoys the doubly robustness property in the sense that $\hat{\beta }$ is still consistent if either $Q^{m}=Q$ or $\pi^{m}=\pi$. When $Q^{m}\neq Q$ and $\pi^{m}=\pi$, we have ${\left\|\hat{\beta }-\beta^{\text{*}}\right\|}_{1}=O_{p}\left(s^{\text{*}}\text{max}\left\{{\left(\text{log }p/n\right)}^{1/2},n^{-\alpha }\right\}\right)$; when $\pi^{m}\neq \pi$ and $Q^{m}=Q$, we have ${\left\|\hat{\beta }-\beta^{\text{*}}\right\|}_{1}=O_{p}\left(s^{\text{*}}\text{max}\left\{{\left(\text{log }p/n\right)}^{1/2},n^{-\beta }\right\}\right)$. This also indicates that as long as one of the estimators $\hat{\pi }$ and $\hat{Q}$ has a reasonably fast rate, the estimator $\hat{\beta }$ is consistent.

Theorems 4 and 5 provide the uniform validity of the testing procedures in Algorithm 1 and the confidence interval constructed using the pooled one-step estimator ${\tilde{\beta }}_{j}$‘s in Algorithm 2 via sample-splitting, respectively.

**Theorem 4** *Assume that Conditions (C1)*–*(C5) hold. For* Algorithm 1, under the null hypothesis $H_{0}:\beta_{j}^{\text{*}}=0,\forall j\in 𝒥\subset \left\{1,\cdots ,p\right\}$, *by choosing $\lambda_{n,k}≍{\tilde{\lambda }}_{n,k}≍{\left(\text{log}p/n\right)}^{1/2}$*, *we have*

$$\underset{j\in 𝒥}{\text{max}}\underset{\alpha \in \left(0,1\right)}{\text{sup}}\left|P\left(\left|\sigma_{j}^{-1}n^{1/2}S_{j}\right|\leq \Phi^{-1}\left(1-\alpha /2\right)\right)-\left(1-\alpha \right)\right|=o_{p}(1).$$

*and ${\text{max}}_{j}\left|{\hat{\sigma }}_{j}-\sigma_{j}^{2}\right|=o_{p}\left(1\right)$*, *where* ${\hat{\sigma }}_{j}^{2}$ *is given in* Algorithm 1, *and*

$$\sigma_{j}^{2}={\left(\nu_{j}^{\text{*}}\right)}^{⊤}\text{var}\left[\nabla^{2}l_{\phi }\left(\beta^{\text{*}};\Omega_{+},\Omega_{-}\right)\right]\nu_{j}^{\text{*}}.$$

**Theorem 5** *Assume that Conditions (C1*)–*(C5) hold. The pooled one-step estimator satisfies*

$$\underset{j}{\text{max}}\underset{\alpha \in \left(0,1\right)}{\text{sup}}\left|P\left(\left|n^{1/2}\left({\tilde{\beta }}_{j}-\beta_{j}^{\text{*}}\right){\hat{I}}_{j|-j}/{\hat{\sigma }}_{j}\right|\leq \Phi^{-1}\left(1-\alpha /2\right)\right)-\left(1-\alpha \right)\right|=o_{p}\left(1\right).$$

**Remark 6** *Theorems 4 and 5 assume that both the propensity and the outcome models are correctly specified and estimated*. *Nonetheless, when the propensity score is known by the design of the experiment, the conclusions in Theorems 4 and 5 still hold even if the outcome model is misspecified. In contrast, Q-learning requires correctly specified outcome models even when the propensity is known. In practice, an individualized treatment rule can still be linear even if the contrast function is non-linear. As such, our modeling framework is more flexible. The advantages of our methods extend to the high-dimensional setting. The outcome weighted learning approach does not involve modeling outcomes. However, the corresponding penalized estimator in the outcome weighted learning approach may have a slower convergence rate than the proposed estimator in Theorem 3 when the propensity score is estimated with a slow rate. Therefore, the de-correlated score or the one-step estimator based on the outcome weighted learning approach cannot achieve a limiting distribution with $n^{1/2}$ convergence rate as in Theorems 4 and 5*.

To derive the asymptotic property of the inference procedure for the value, we further introduce the following conditions:

(C6) There exists an increasing function ψ such that 1) ψ(0)=0; 2) there exists ζ>0 and ${\text{lim sup}}_{t\to 0}\psi \left(t\right)/t^{\zeta }<+\infty ;3)\left|E\left(Y\left(1\right)-Y\left(-1\right)|X\right)\right|\leq \psi \left(\left|X^{⊤}\beta^{\text{*}}\right|\right)$ when $\left|X^{⊤}\beta^{\text{*}}\right|\leq t_{0}$, where $t_{0}$ is a constant.

(C7) There exist constants γ>0 and $C_{\gamma }>0$ such that for any t in some neighborhood of 0, we have that $P\left(0<\left|X^{⊤}\beta^{\text{*}}\right|\leq t\right)\leq C_{\gamma }t^{\gamma }$.

**Theorem 7** *Assume that* Y *is bounded and denote the sample size of ${\tilde{I}}_{1}$ as* $n_{1}$ *and* ${\tilde{I}}_{2}$ *as $n_{2}$*. *In addition to the conditions in Theorem 3, we further assume $n_{1}^{-\alpha -\beta }n_{2}^{1/2}=o\left(1\right)$ and one of the following conditions: 1) Conditions (C6) and (C7) holds with ${\left(s{\left(\text{log}p/n_{1}\right)}^{1/2}\right)}^{\zeta +\gamma }=o_{p}\left(n_{2}^{-1/2}\right)$*; 2) Condition (C7) holds with $P\left(\left|X^{⊤}\beta^{\text{*}}\right|=0\right)=0$ and ${\left(s{\left(\text{log}p/n_{1}\right)}^{1/2}\right)}^{\gamma }=o_{p}\left(n_{2}^{-1/2}\right)$, *then we have*

$$n_{2}{\;}^{1/2}\sigma_{V}^{-2}\left(\hat{V}\left(\hat{D}\right)-V\left(D^{\text{*}}\right)\right)\to N\left(0,1\right)$$

*where $\sigma_{V}^{2}=\text{var}\left[W_{\hat{D}\left(X\right)}\left(Y,X,A,\pi ,Q\right)\right]$*.

Condition (C6) implicitly assumes that $\beta^{\text{*}}$ corresponds to the optimal individualized treatment rule. When Condition (C6) fails, the inference of the value under $D^{\text{*}}\left(X\right)$ requires stronger assumptions (see Theorem 9 in Appendix D for details). In the simulation studies and application, we choose $n_{1}=n_{2}=n/2$.

## 4. Simulation Studies

In this section, we test our estimation and inference procedure under various simulation scenarios. Let Δ(X)={Q(1;X)−Q(−1;X)}/2 and S(X)={Q(1;X)+Q(−1;X)}/2. We generate $X\sim N\left(0,I_{p\times p}\right)$, and Y=AΔ(X)+S(X)+ϵ,ϵ~N(0, 1). Let $\beta^{\text{opt}}={\left(1,\;1,-1,-1,\;0,\ldots ,0\right)}^{⊤},\beta_{S}^{\text{*}}={\left(-1,-1,\;1,-1,\;0,\ldots ,0\right)}^{⊤}$, and $\beta_{\pi }^{\text{*}}={\left(1,-1,\;0,\ldots ,0\right)}^{⊤}$. The following scenarios are considered: (I) $\Delta \left(X\right)=\xi X^{⊤}\beta^{\text{opt}},S\left(X\right)=0.4X^{⊤}\beta_{S}^{\text{*}}$, and $\pi \left(1;X\right)=\text{exp}\left(0.4X^{⊤}\beta_{\pi }^{\text{*}}\right)/\left\{1+\text{exp}\left(0.4X^{⊤}\beta_{\pi }^{\text{*}}\right)\right\};$ (II) $\Delta \left(X\right)=\left\{\Phi \left(\xi X^{⊤}\beta^{\text{opt}}\right)-0.5\right\}\times \tilde{\Delta }\left(X\right),S\left(X\right)=\text{exp}\left(0.4X^{⊤}\beta_{S}^{\text{*}}\right),\pi \left(1;X\right)=\text{exp}\left\{\left(X_{1}^{2}+X_{2}^{2}+X_{1}X_{2}\right)/4\right\}/\left[1+\text{exp}\left\{\left(X_{1}^{2}+X_{2}^{2}+X_{1}X_{2}\right)/4\right\}\right]$, where $\tilde{\Delta }\left(X\right)=2{\left(\sum_{j=1}^{4}X_{j}\right)}^{2}+2\xi$ and Φ(⋅) is the cdf of the standard normal distribution.

Under these settings, the magnitude of the treatment effect Δ(X) changes with ξ, which ranges from 0.1 to 1. Scenario (I) features a linear outcome model Q(a;X) for both a=1 and a=−1, and a logistic model for the propensity. Scenario (II) has a nonlinear treatment effect Δ(X), though the decision boundary is still linear. The treatment assignment mechanism is also complex. More simulation results with a mixture of both discrete and continuous covariates, as well as highly correlated design matrices and non-regular cases, can be found in Appendix A.

We compare the pooled estimator with Q-learning, a regression-based method (Qian and Murphy, 2011). With high-dimensional covariates, we fit a linear regression with a lasso penalty in Q-learning for all scenarios. The inference target of interest is $\beta^{\text{opt}}$. However, the limits of the coefficients estimates using either proposed method or Q-learning may not be identical to $\beta^{\text{opt}}$. In our simulation experiments, we will test and construct confidence intervals for $\beta_{j}^{\text{*}}$’s, j=1,…,8, the j-th coordinate of $\beta^{\text{*}}$, which by abuse of notations, denote the limits of estimates under either method. We generate large data sets multiple times using the same data-generating process, and empirically verify that the sparsity pattern of $\beta^{\text{*}}$ matches with that of $\beta^{\text{opt}}$. Hence, inferences on $\beta^{\text{*}}$ provide insights on the true optimal decisions. We conduct the hypothesis testing for Q-learning using the de-correlated score test proposed in Ning and Liu (2017), and construct 95% confidence intervals for the coefficients of interest in the context of Q-learning. For value inference, we implement the Algorithm 3 as our proposed approach; for Q-learning, we implement the Algorithm 3 with the coefficients $\hat{\beta }$ estimated from Q-learning approach. The true value $V\left(\beta^{\text{*}}\right)$ is approximated by the average of estimated values on a large independent data set. An R package called ITRInference (see https://github.com/muxuanliang/ITRInference.git) is coded to implement the proposed method and Q-learning approach. For the proposed method, the user can specify the method or select from a list of candidates to estimate nuisance parameters. In our implementation, we choose to estimate π and Q functions nonparametrically for all scenarios. To be more specific, we first implement a distance correlation-based variable screening procedure (Li et al., 2012). We then fit a kernel regression using the selected variables after screening. When estimating π, we set caps at 0.1 and 0.9 to trim extreme values.

In all scenarios, the sample size n and the dimension p range from 350, 500, 800, 1600, 2500 to 8000. We set the nominal significance level at 0.05, and the nominal coverage at 95%. We report the type I errors, the powers of the hypothesis tests, and the value functions under the estimated decision rules out of 500 replications. In particular, we present the type I errors for testing $\beta_{5}^{\text{*}}$ to $\beta_{8}^{\text{*}}$, and the powers for testing $\beta_{1}^{\text{*}}$ to $\beta_{4}^{\text{*}}$. For each method, we also present the coverage of the interval estimations around the limiting coefficients. We also present the bias and the length of the confidence interval for the coefficients estimations and value estimations.

**Figure 1:**
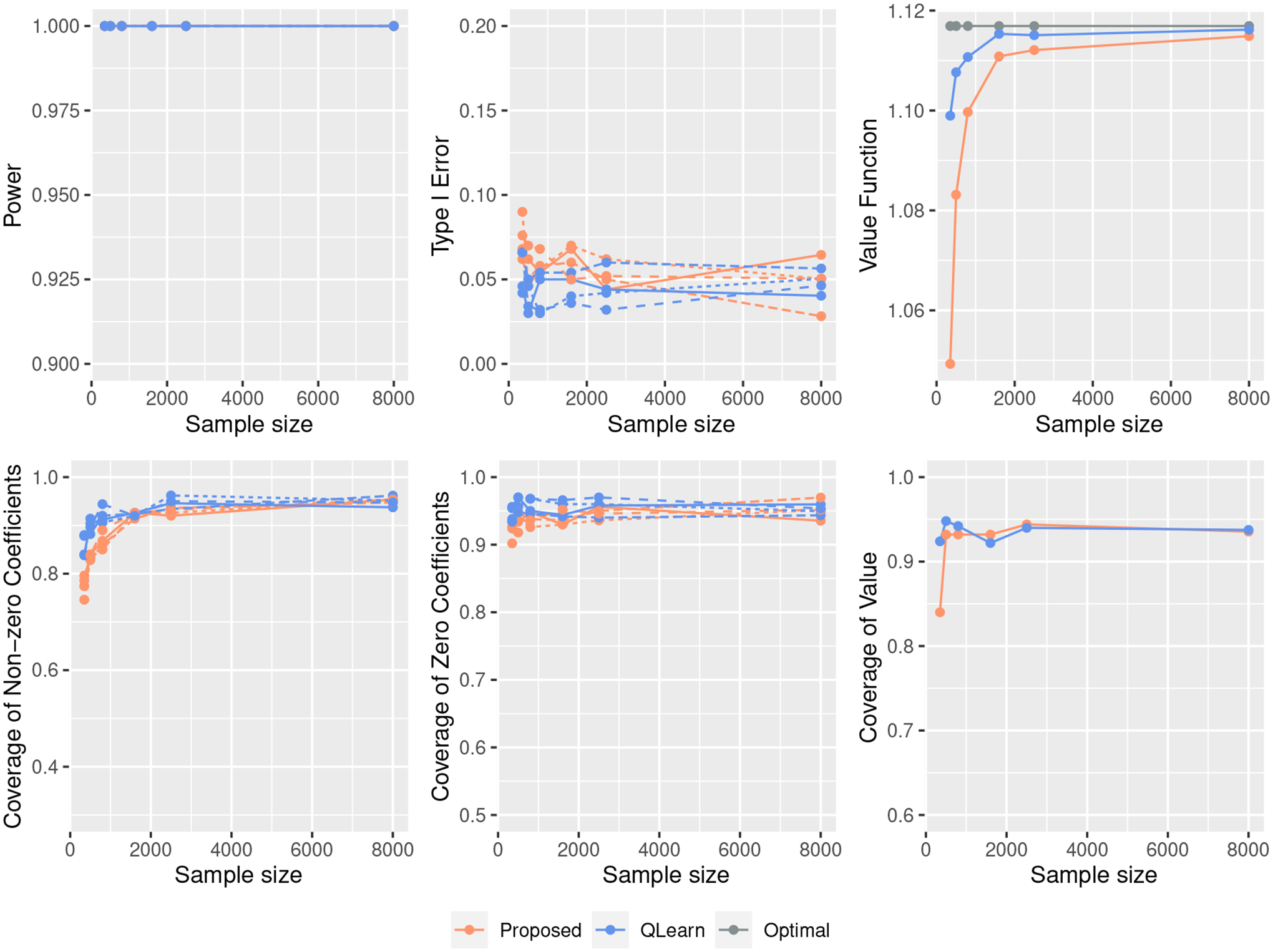
Simulation results for Scenario (I) with the change of sample size when ξ=0.7 and p=2500. Types of the line represent different coefficients.

**Figure 2:**
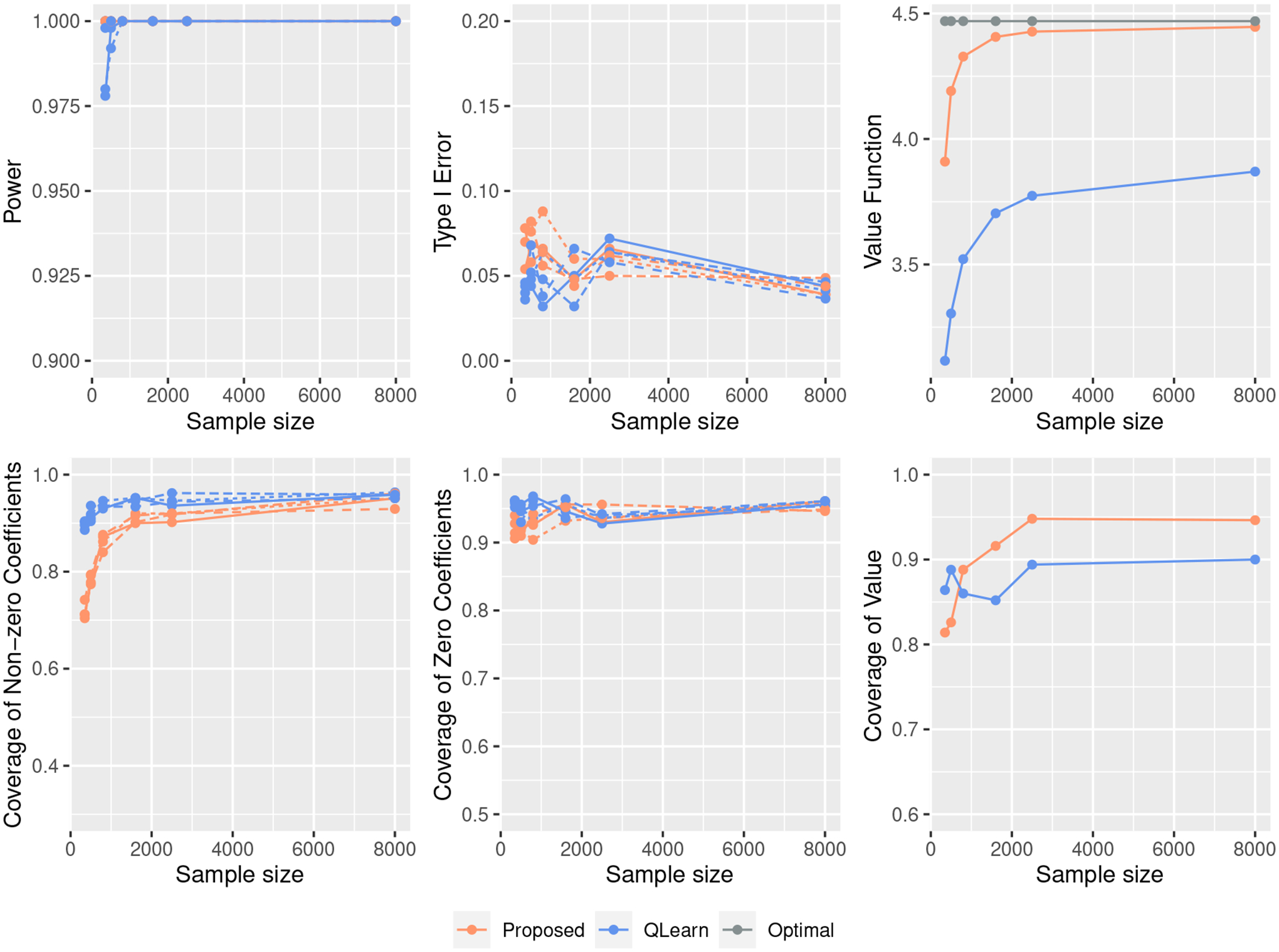
Simulation results for Scenario (II) with the change of sample size when ξ=0.8 and p=2500. Types of the line represent different coefficients.

**Figure 3:**
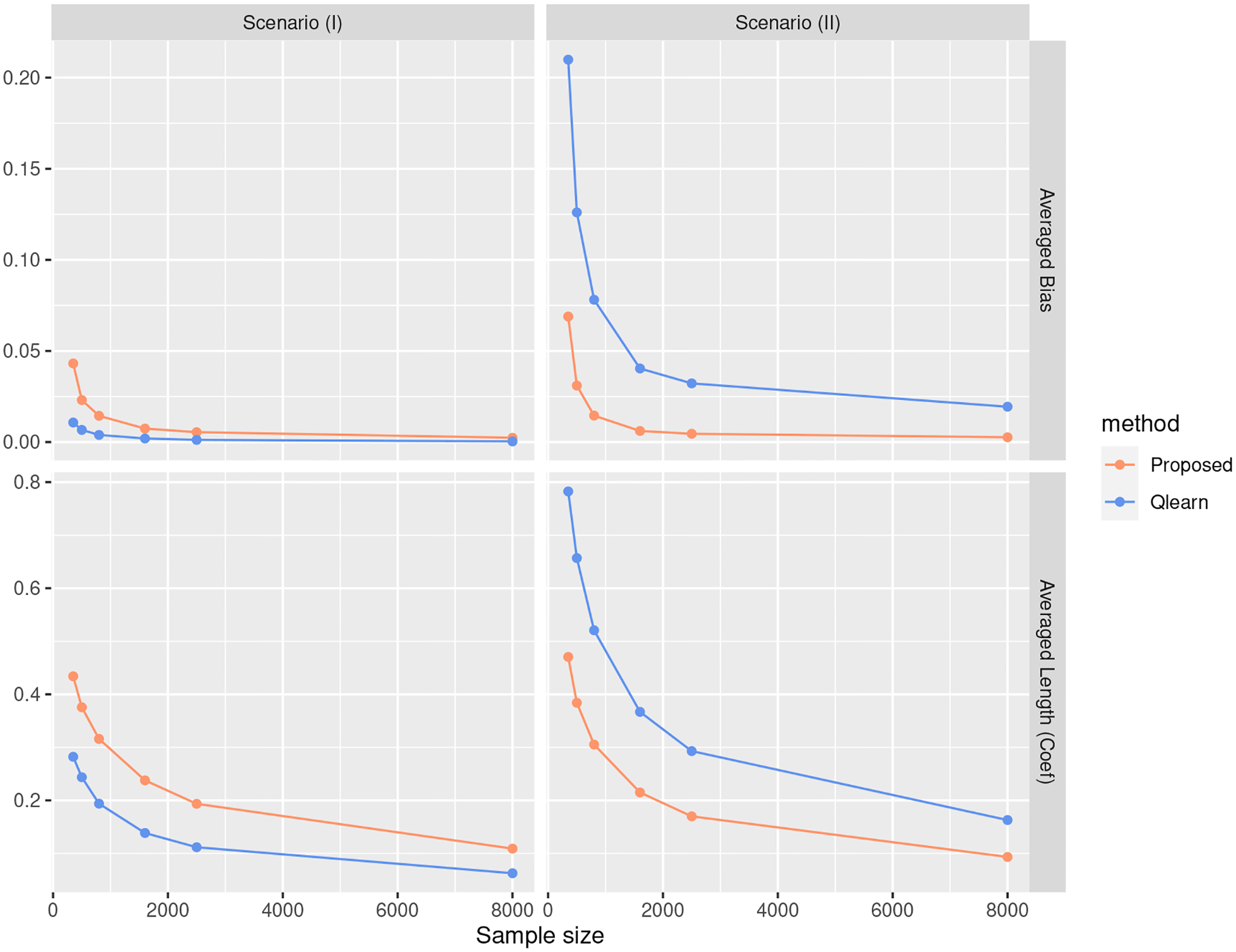
Simulation results in terms of the averaged bias of the coefficient estimates and the averaged length of the confidence intervals.

Figures 1 – 3 show the simulation results for different scenarios, with the sample size n varied and the p and ξ fixed. Additional results on varying p with n and ξ fixed can be found in Appendix A. As expected, in Scenarios (I) (Figure 1) where the regression model is correctly specified for Q-learning, Q-learning yields a better value function. Conversely, the proposed method outperforms the Q-learning method in Scenario (II) (Figure 2). In terms of the type I error and power, the proposed method is comparable to the Q-learning approach in Scenario (I) (Figure 1). For Scenarios (II) (Figure 2), our method is more powerful, and the type I errors are well controlled. Figure 3 also shows that the proposed method leads to less biased point estimations and shorter confidence intervals. The excessive bias of point estimations and lengths of interval estimations for the Q-learning approach may be due to the model misspecification. The coverage of $\beta_{5}^{\text{*}}$ to $\beta_{8}^{\text{*}}$ are concentrated near 95%, and the coverage of the $\beta_{1}^{\text{*}}$ to $\beta_{4}^{\text{*}}$ gradually approach 95% for the proposed method. For the coverage of the value $V\left(\beta^{\text{*}}\right)$, the inference procedure achieves a valid CI for the value under the proposed approach in both scenarios when the sample size is large enough. However, the inference for the value under the Q-learning is under-coverage due to the model misspecification. In Appendix A, we also compare the proposed value inference procedure with bootstrap methods in terms of the coverage probabilities and lengths of confidence intervals.

## 5. Application to Complex Patients with Type-II Diabetes

In this section, we apply our proposed estimation and inference procedures to construct the optimal individualized treatment rule for complex patients with type-II diabetes. The data are collected from the electronic health records through Health Innovation Program at University of Wisconsin. The entire data set includes n=9101 patients. There are 40 covariates, including socio-demographic variables, previous disease experiences, and baseline HbA1c levels, etc. The outcome is the indicator whether the patient successfully controls the HbA1c below 8% after a year. The treatment A=1 if the patient received any medications, including insulin, sulphnea or OHA, and A=−1 otherwise. Among 9101 patients, 17.1% had a missing post-treatment HbA1c measurement, and 15.4% had the missing baseline HbA1c measurements. We impute missing values using Multivariate Imputation by Chained Equations (MICE package in R), which is based on the estimated conditional distributions of each covariate given other covariates (van Buuren and Groothuis-Oudshoorn, 2011). To address the possible interactions among covariates, we consider both raw covariates and all first-order interactions. We rank these covariates by their variances and select p=100 covariates with top variances.

We split the data set into a training data set (80% of the entire data set) and a testing data set (20% of the entire data set). The proposed method and Q-learning are fitted on the training data set using the same strategies as described in simulation studies. To evaluate these estimated decision rules, we calculate the value function by $E_{n}\left[Y1\left\{A=\hat{D}\left(X\right)\right\}/{\hat{\pi }}_{0}\right]$, on the testing data set, where $\hat{D}$ is the estimated decision rules on the training data set and ${\hat{\pi }}_{0}$ is the estimated propensity scores on the testing data set. The entire procedure is repeated 100 times with random training and testing data splits. The mean and standard deviation (sd) of the value functions over these repeats are summarized in Table 1. Both the proposed and Q-learning methods construct decision rules that yield better results than the current clinical practice (sd of the difference is 0.0138 (Proposed); 0.0143 (Q-Learning)). Furthermore, our proposed method achieves a higher value function than Q-learning approach as shown in Table 1 (sd of the difference is 0.0115).

Next, we conduct the inference procedure to identify driving factors of the optimal individualized treatment rule as well as to provide an interval estimation using the entire data set. Results are presented in Table 2. After controlling for the false discovery rate below 0.05, our results indicate that a female patient with a higher HbA1c value at baseline is more likely to benefit from the treatment. The figure comparing the list of significant covariates selected by the proposed method and Q-learning can be found in Appendix C.

## 6. Discussion

In this paper, we consider a single-stage problem and assume a high-dimensional linear decision rule. In practice, especially in managing chronic diseases, dynamic treatment regimes are widely adopted, where sequential decision rules for individual patients adapt overtime to the evolving disease. One future direction is to develop inferential methods in the multi-decision setup. We can also extend the linear decision rule to a single index decision rule $d\left(X^{⊤}\beta^{\text{*}}\right)$, where d is an unknown function. Throughout, we require that the surrogate loss function be differentiable. A non-differentiable surrogate loss such as the hinge loss does not have a well-defined Hessian, which hinders the construction of the decorrelated score. This can be addressed by a smoothed hinge loss or an approximation of the Hessian. We are currently working on these possible extensions.

In this work, we adopt the de-correlated score to infer the high-dimensional linear decision rule. It is also possible to use other high-dimensional influential tools developed recently. Partial penalized tests proposed in Shi et al. (2019) allow to test hypotheses involving a growing number of coefficients as the sample size increases. Ma et al. (2021) consider the global and simultaneous hypothesis testing for high-dimensional logistic regression models. Although a modified algorithm 1 can be combined with these methods, its theoretical property, especially the consequences of nuisance parameter estimation with slow rates, needs future investigations. In addition, this work can be extended to test multi-dimensional hypotheses, i.e., ${ℋ}_{0}:\beta_{j}^{\text{*}}=0,j\in 𝒢$, where 𝒢 is a subset of {1,⋯,p}. For a low-dimensional sub-vector, .i.e, |𝒢|, the proposed de-biased estimators can be used to construct p-values or confidence regions. For a high-dimensional sub-vector, i.e. 𝒢, the current procedure can be extended as well. However, the required relationship among α, β, p, and n needs future investigation. Thus, we will leave it as future work.

In addition, in this work, we assume that both nuisance parameters are correctly specified and estimated by nonparametric methods after the variable screening. Some recent literature in estimating the average treatment effect assumes that only one of the nuisance parameters is correctly specified (Athey et al., 2018; Ning et al., 2020; Tan, 2020; Smucler et al., 2019). In these approaches, the correct specified nuisance parameter is assumed to follow a structural model such as linear or partially linear models. It would be interesting to investigate how to extend these approaches to ITR inference.

In this work, we approximate the indicator function by a smooth convex surrogate. In addition to a smooth surrogate, many other non-smooth or non-convex loss functions such as the hinge loss (Cortes and Vapnik, 1995), the ramp loss (Collobert et al., 2006a, b) and the ψ-learning loss (Shen et al., 2003) can be considered to approximate the indicator function. Especially, the non-convex loss function such as the ramp loss and the ψ-learning loss is more robust to the presence of outliers. However, the non-convex/non-smooth surrogate loss may be hard to optimize and the non-convexity creates an additional barrier in high-dimensional inference. We would consider this extension as our future work.

Another future work is to extend the proposed approach to multiple treatment options setup. There are several possible directions. The first direction is to transform the multiple treatment problem into multiple binary decision problems. We can consider a sequential decision-making strategy (Zhou et al., 2018) by conducting a series of binary treatment selections. It is shown that such strategy is Fisher consistent. Another direction is to adopt techniques used in multi-label classification problems to estimate the optimal individualized treatment rule (Liang et al., 2018a). We can incorporate the weights based on the outcome model and propensity model into this framework and develop the corresponding inferential procedures. We are currently working on these extensions.

## Supplementary Material

Appendix A and Appendix B

## Figures and Tables

**Table 1:** Comparisons on value functions.

	Mean	Standard deviation
Observed	0.860	0.008
Proposed approach	0.877	0.015
Q-Learning	0.869	0.015

**Table 2:** Coefficients and p-value for the significant covariates of the estimated decision rule (CIs are included in Appendix C). Special chronic conditions refer to chronic conditions including amputation, chronic blood loss, drug abuse, lymphoma, metastatistic cancer, and peptic ulcer disease. Bucketized age refers to a variable created by bucketizing the raw age by its observed quartiles. Other Race refers to the race excluding White and Black.

	Coef	P-value
Chronic Complications : Fluid and Electrolyte Disorders	−0.024	4:71 × 10^−2^
Chronic Complications : African American	−0.027	3:58 × 10^−2^
Alcohol Abuse : Entitlement Disability	−0.054	3:33 × 10^−2^
HCC Community Score : Special Chronic Conditions	−0.022	2:99 × 10^−2^
Hypertension : Lower Extremity Ulcer	−0.036	2:39 × 10^−2^
HbA1c at Baseline : African American	0.019	2:26 × 10^−2^
Entitlement Disability : Hypothyroidism	−0.024	2:25 × 10^−2^
Cardiac Heart Failure : Peripheral Vascular Disease	−0.029	2:24 × 10^−2^
Chronic Kidney Disease : HbA1c at Baseline	0.081	1:97 × 10^−2^
Other Race : Special Chronic Conditions	0.016	1:95 × 10^−2^
Liver Disease : Weight Loss	0.015	1:72 × 10^−2^
Other Neurological Disorders : Female	−0.021	1:28 × 10^−2^
Lower Extremity Ulcer : HbA1c at Baseline	0.039	9:60 × 10^−3^
Chronic Complications : Bucketized Age	0.040	9:05 × 10^−4^
HbA1c at Baseline : Female	0.044	8:47 × 10^−8^
