# Supplementary material for: Estimation and inference on high-dimensional individualized treatment rule in observational data using split-and-pooled de-correlated score
Source: J Mach Learn Res. Author manuscript; Available in PMC 2023 Dec 14. (PMC10720606)
Supplement: Appendix A and Appendix B [file NIHMS1949967-supplement-Appendix_A_and_Appendix_B.pdf]

Appendix A.

In Appendix A, we provide additional simulation results.

Simulation Results with ξ and p Changed

In this section, we provides more simulation results. Let $\Delta(\boldsymbol{X}) = \{Q(1;\boldsymbol{X}) - Q(-1;\boldsymbol{X})\}/2$ and $S(\boldsymbol{X}) = \{Q(1;\boldsymbol{X}) + Q(-1;\boldsymbol{X})\}/2$. We generate $\boldsymbol{X} \sim N(0, \boldsymbol{I}_{p\times p})$, and $Y = A\Delta(\boldsymbol{X}) + S(\boldsymbol{X}) + \epsilon$, where $\epsilon \sim N(0,1)$ is the random error.

Denote $\boldsymbol{\beta}^{\text{opt}} = (1,1,-1,-1,0,\cdots,0)^\top$, $\boldsymbol{\beta}^*_S = (-1,-1,1,-1,0,\cdots,0)^\top$, and $\boldsymbol{\beta}^*_\pi = (1,1,1,0,-1,0,-1,0,\cdots,0)^\top$. The following scenarios are considered:
(I) $\Delta(\boldsymbol{X}) = \xi\boldsymbol{X}^\top\boldsymbol{\beta}^{\text{opt}}$, $S(\boldsymbol{X}) = 0.8\boldsymbol{X}^\top\boldsymbol{\beta}^*_S$, and $\pi(1;\boldsymbol{X}) = 1/\left\{1+\exp(-0.4\boldsymbol{X}^\top\boldsymbol{\beta}^*_\pi)\right\}$;
(II) $\Delta(\boldsymbol{X}) = \left\{\Phi\left(\xi\boldsymbol{X}^\top\boldsymbol{\beta}^{\text{opt}}\right) - 0.5\right\}\times\widetilde{\Delta}(\boldsymbol{X})$, $S(\boldsymbol{X}) = \exp\left(0.4\boldsymbol{X}^\top\boldsymbol{\beta}^*_S\right)$, $\pi(1;\boldsymbol{X}) = \exp((X_1^2 + X_2^2 + X_1X_2)/4)/\left\{1+\exp((X_1^2+X_2^2+X_1X_2)/4)\right\}$, where $\widetilde{\Delta}(\boldsymbol{X}) = 2(\sum_{j=1}^4 X_j)^2 + 2\xi$ and $\Phi(\cdot)$ is the cdf of the standard normal distribution.

Under these settings, the magnitude of the treatment effect $\Delta(\boldsymbol{X})$ changes with ξ, which ranges from 0.1 to 1. In all scenarios, the sample size n and the dimension p range from 350, 500, 800, 1600, 2500, to 8000. When p changes from 350 to 2500, these settings include both low-dimensional ($p = 350$, $n = 2500$) and high-dimensional ($n = 350$, $p = 2500$) settings. We set the nominal significant level at 0.05, and the nominal coverage at 95%. We report the type I error, the power of the testing, and the value functions under the estimated decision rules out of 500 replications. For simplicity, we only test for β_l^*'s, $l = 1,\cdots,8$, where β_l^* is the l-th coordinate of $\boldsymbol{\beta}^*$.

Figuress 4 and 5 show the simulation results for Scenario (I) and (II). Figure 4 shows that when we fix $n = 500$, the proposed method suffers from the high-dimensional nonparametric estimation of Q compared with Q-learning in terms of the value function as p increases. Figure 5 shows that the proposed method dominates the Q-learning in terms of the value function no matter how ξ and p changes.

Non-regular Scenarios

In this section, we provides two additional simulation scenarios. These two scenarios share the same conditional distribution of Y given $A, \boldsymbol{X}$ but have different design matrix. Both scenarios are non-regular in the sense that there exists a subgroup of patients for whom treatment is neither beneficial nor harmful. Specifically, we consider a function $z(t) = (z-0.2)^3 I\{z \geq 0.2\} + (z+0.2)^3 I\{z \leq -0.2\}$. Then we choose $\Delta(\boldsymbol{X}) = z\left(\xi\left\{4\Phi\left(\xi\boldsymbol{X}^\top\boldsymbol{\beta}^{\text{opt}}\right) - 2\right\}\right)$, where $\Phi(\cdot)$ is the cdf of the standard normal distribution; $S(\boldsymbol{X}) = \exp\left(0.4\boldsymbol{X}^\top\boldsymbol{\beta}^*_S\right)$; $\pi(1;\boldsymbol{X}) = \exp(0.25(X_1^2+X_2^2+X_1X_2))/\left\{1+\exp(0.25(X_1^2+X_2^2+X_1X_2))\right\}$.

In Scenario III.a, the design matrix involves only continuous variables. In Scenario III.b, the design matrix involves discrete variables and a dense correlation matrix between covariates. Specially, we consider (III.a) $\boldsymbol{X} \sim N(0, \boldsymbol{I}_{p\times p})$; (III.b) $\widetilde{\boldsymbol{X}}$ follows $N(0,\boldsymbol{\Sigma})$, and then $X_j = I\{j = 4i+3, i \in \mathbb{N}\}I\{\widetilde{X}_j > 0\} + I\{j \neq 4i+3, i\in\mathbb{N}\}\widetilde{X}_j$, where $\boldsymbol{\Sigma}$ is a $p\times p$ matrix with the (i,j)th entry $0.2^{|i-j|}$.

Figures 6 and 7 show the results under non-regular cases. Compared with Q-learning, the proposed method has higher value and comparable testing power. On the coverage of value, the proposed method is less susceptible to model mis-specification compared with

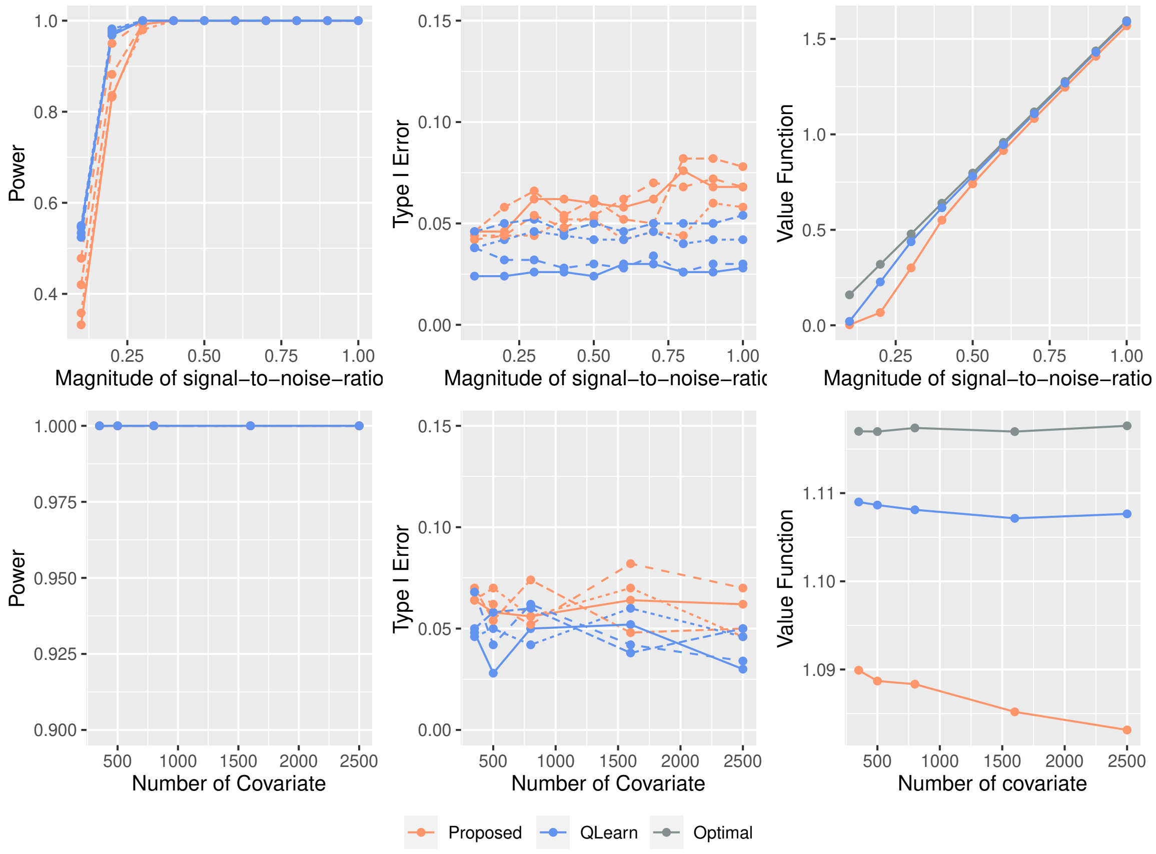

Figure 4: Simulation results for Scenario (I) with the change of ξ ($p = 2500$) and p ($\xi = 0.7$) when $n = 500$. Types of the line represent different coefficients.

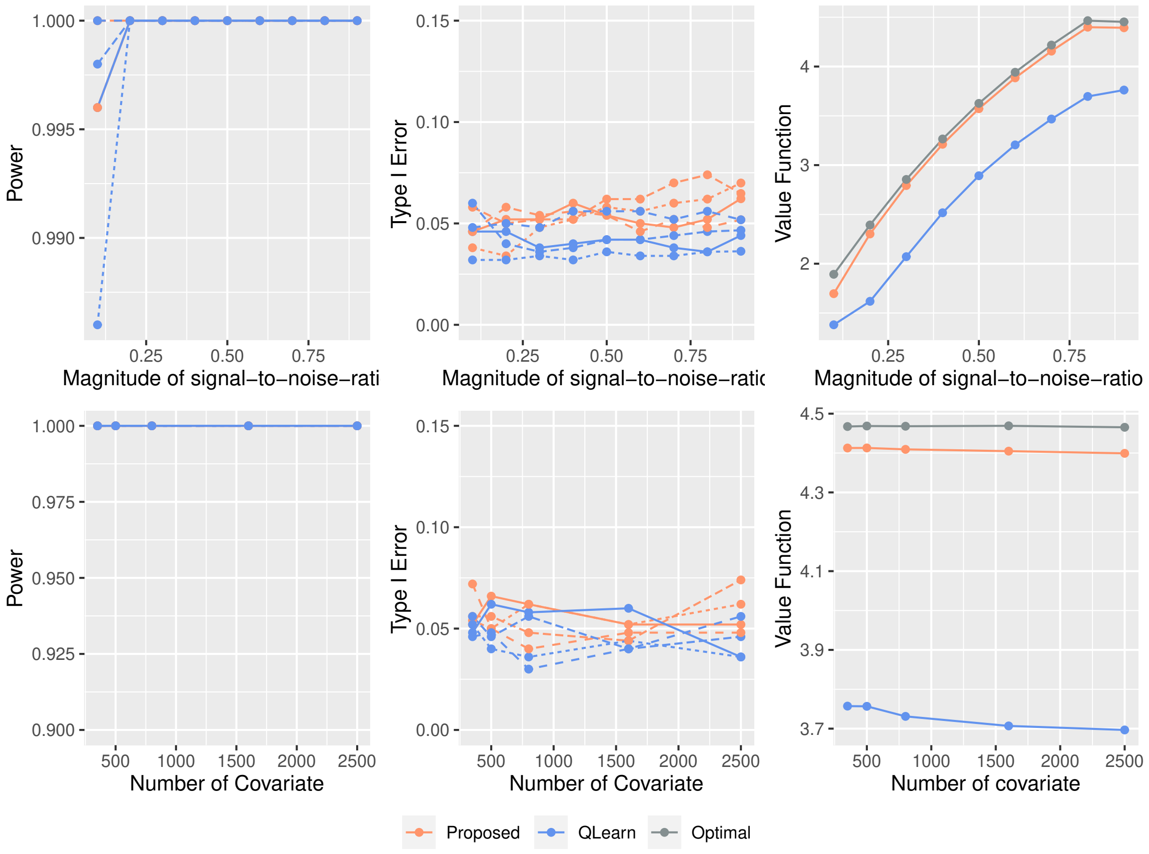

Figure 5: Simulation results for Scenario (II) with the change of ξ ($p = 2500$) and p ($\xi = 0.8$) when $n = 1600$. Types of the line represent different coefficients.

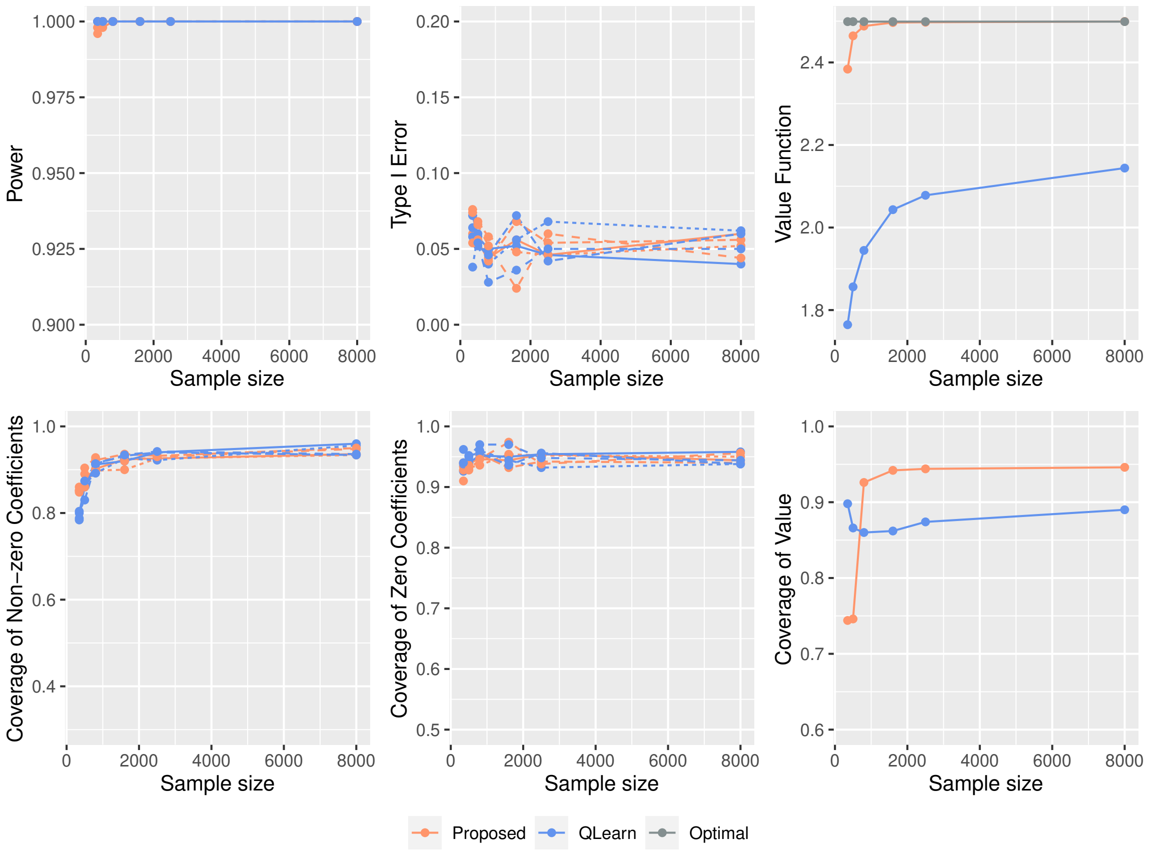

Figure 6: Simulation results for Scenario (III.a) with the change of sample size when $\xi = 0.8$ and $p = 2500$. Types of the line represent different coefficients.

the Q-learning approach. Figures 8 and 9 show the results with changing ξ and p. When ξ increases, the region where treatment effect vanishes gets smaller. Especially, when $\xi = 0.1$, there is no treatment effect for any patients. We can see that the proposed approach has higher value function no matter how ξ and p changes. The Q-learning has slightly worse lower when the non-regularity is severe.

Comparison of Inference Methods for the Optimal Value

In this section, we compare different methods to infer the optimal value. We consider the proposed method (direct), standard bootstrap procedure (bootstrap), and the weighted bootstrap procedure (weightedBootstrap). For the weighted bootstrap procedure, for each bootstrap, we only bootstrap the sampling weighted from a random variable with a mean 1 and unit variance. We do $B = 1000$ bootstraps for each bootstrap-based procedure. For each inference method, we use the PEARL and Q-learning to estimate the optimal decision rules. We follow the data generation procedure in Scenario (I), (II), (III.a), and (III.b) and report the coverages of the optimal value and the lengths of the confidence interval.

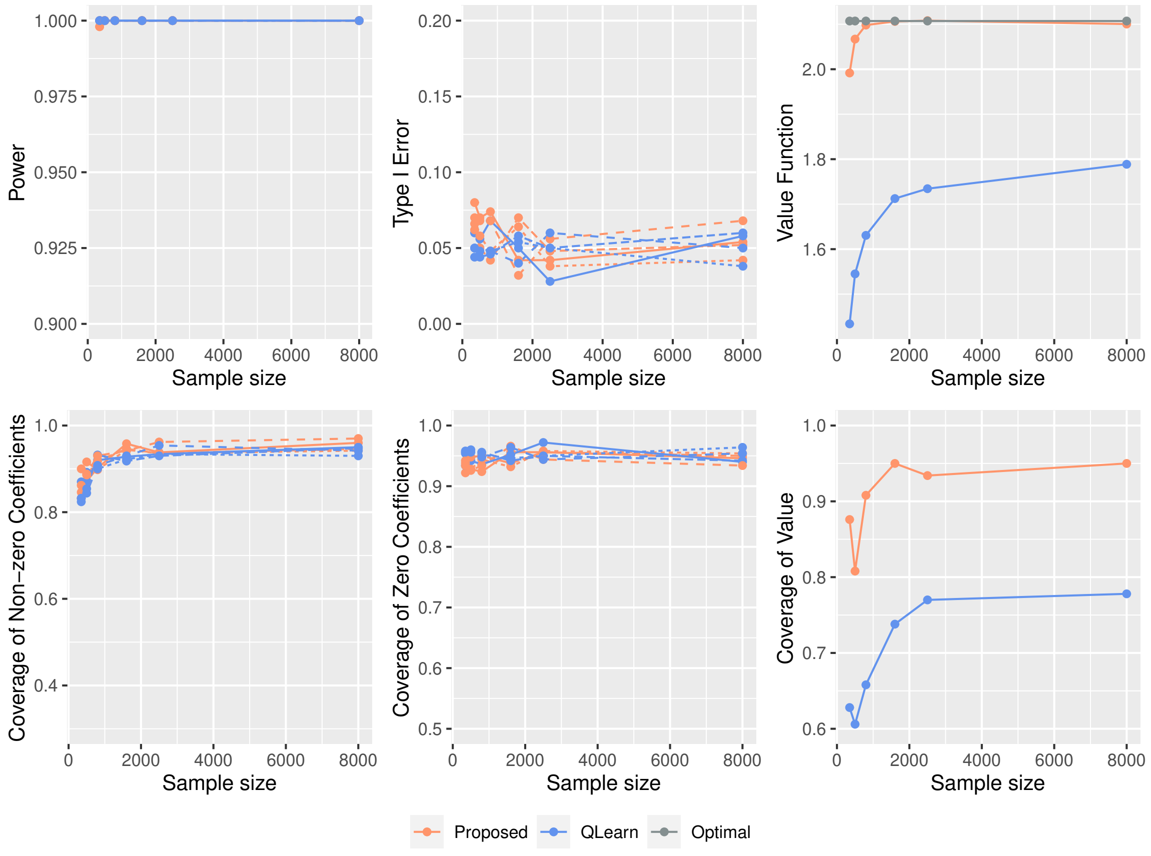

Figure 7: Simulation results for Scenario (III.b) with the change of sample size when $\xi = 0.8$ and $p = 2500$. Types of the line represent different coefficients.

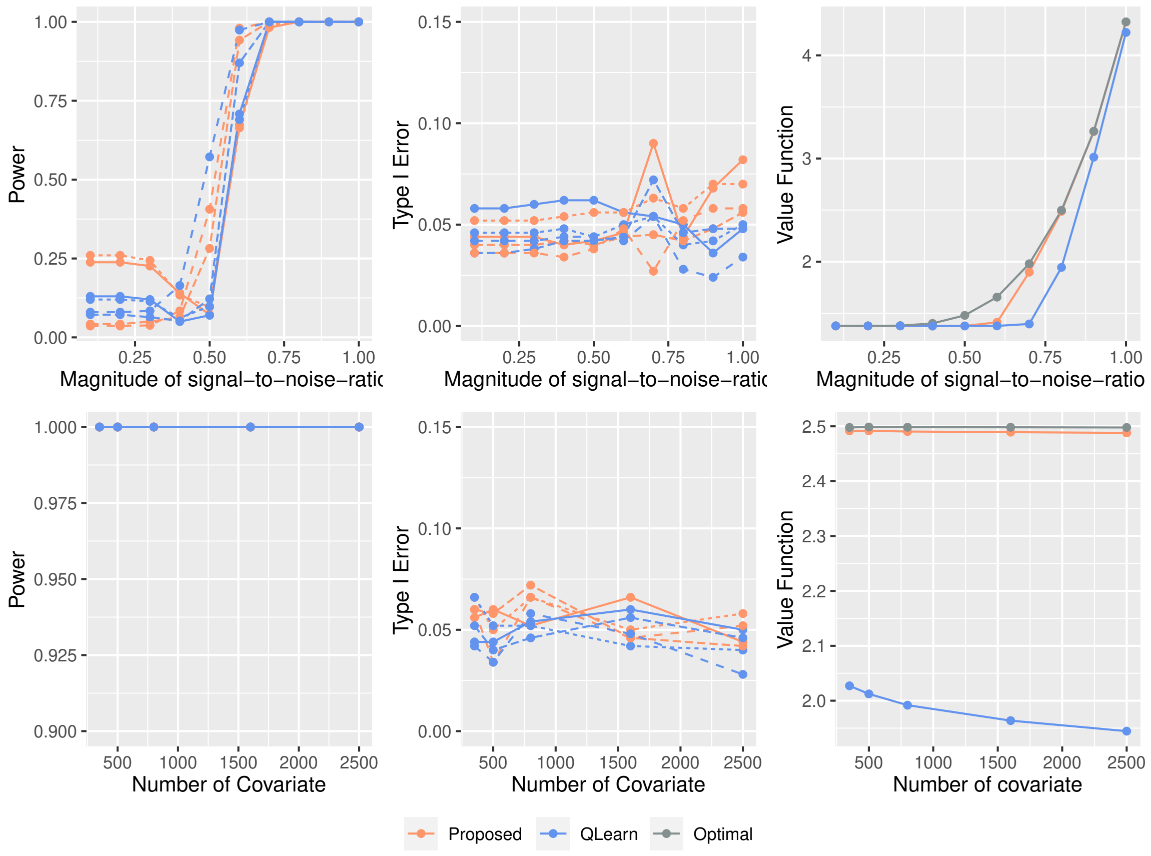

Figure 8: Simulation results for Scenario (III.a) with the change of ξ ($p = 2500$) and p ($\xi = 0.8$) when $n = 800$. Types of the line represent different coefficients.

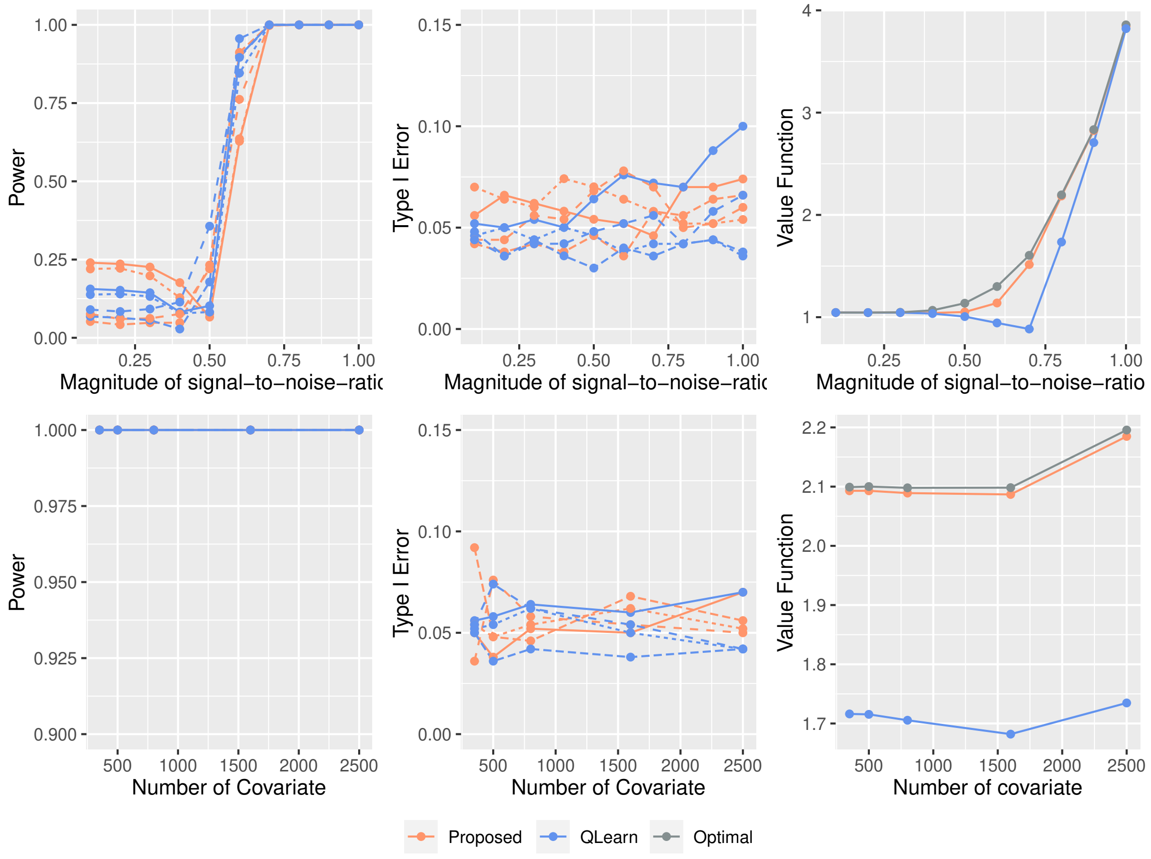

Figure 9: Simulation results for Scenario (III.b) with the change of ξ ($p = 2500$) and p ($\xi = 0.8$) when $n = 800$. Types of the line represent different coefficients.

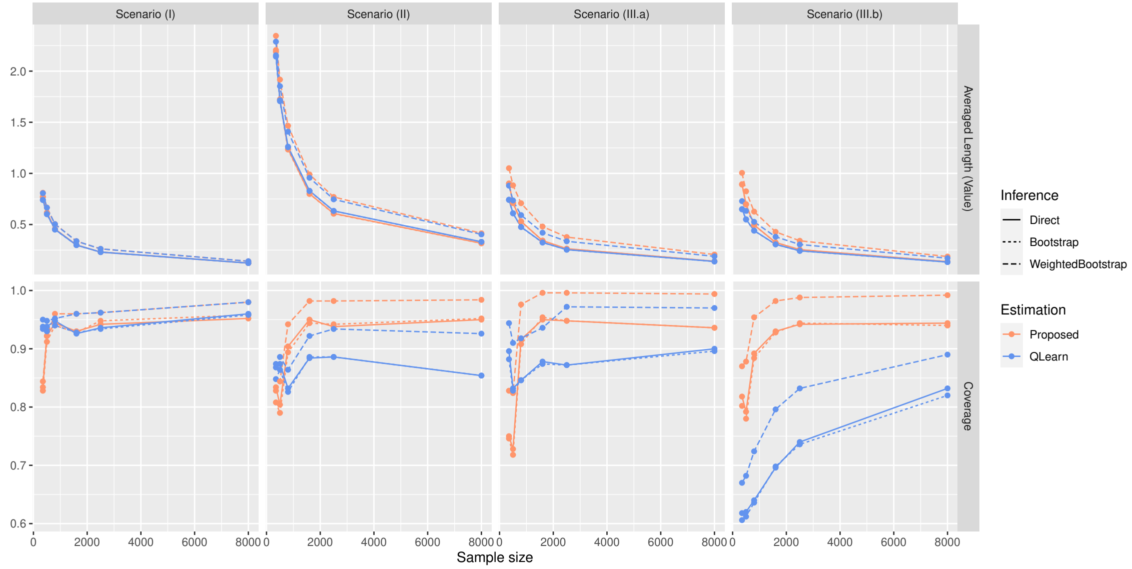

Figure 10: Simulation results for value inference.

From Figure 10, the proposed method has a similar performance to the standard bootstrap method in terms of the coverages and lengths of the CIs. However, the weighted bootstrap leads to slightly over-coverage, especially for non-regular settings, i.e., Scenario (III.a) and (III.b).

Illustration of Double Robustness

In this section, we check the double robustness of the proposed method under either misspecified propensity model or outcome models. We generate the data following Scenario (II) and consider alternative methods to estimate the propensity and outcome models. Specifically, we consider 1) the proposed method with non-parametrically estimated propensity and outcome models (Both Correct); 2) the proposed method with a non-parametrically estimated propensity and outcome models estimated by linear regressions (Outcome Missed); 3) the proposed method with non-parametrically estimated outcome models and a propensity estimated by logistic regression (Propensity Missed). Figure 11 shows that as the sample size increases, the values of the decision rules derived by all these approaches achieve the optimal value. This implies that our proposed method is consistent if either the outcome models or the propensity is correctly specified.

Appendix B.

In Appendix B, we provide a sufficient condition and examples for $\boldsymbol{\beta}^* \propto \boldsymbol{\beta}^{\text{opt}}$. Lemma 1 provides sufficient conditions that $\boldsymbol{\beta}^*$ satisfies $D_{\text{opt}}(\boldsymbol{X}) = \text{sgn}(\boldsymbol{X}^\top \boldsymbol{\beta}^{\text{opt}}) = \text{sgn}(\boldsymbol{X}^\top \boldsymbol{\beta}^*)$, which indicates the inference of $\boldsymbol{\beta}^*$ is equivalent to that of $\boldsymbol{\beta}^{\text{opt}}$. First, we show the proof of Lemma 1.

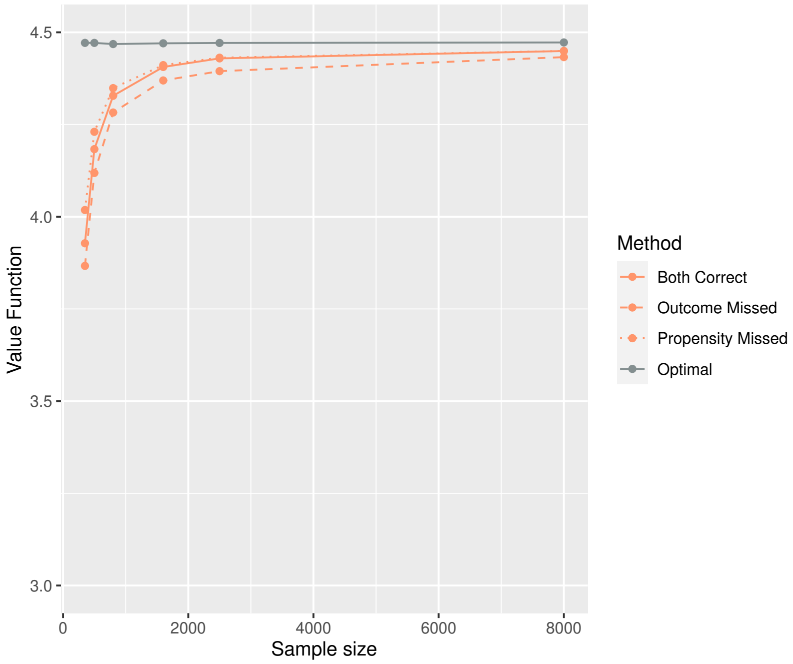

Figure 11: Simulation results for double robustness.

Proof. First, we provide a proof for Lemma 1. Under the Lemma 1, we show that $\boldsymbol{\beta}^* \propto \boldsymbol{\beta}^{\text{opt}}$. Without loss of generality, we assume that W_a's are non-negative.

For $\beta = \boldsymbol{\beta}^*$, we have the following

$$
\begin{aligned}
& E\left[E(\Omega_+ \mid \boldsymbol{X})\phi\left(\boldsymbol{X}^\top\boldsymbol{\beta}\right) + E(\Omega_- \mid \boldsymbol{X})\phi(-\boldsymbol{X}^\top\boldsymbol{\beta})\right] \\
= \; & E\left[\Delta/2\left\{\phi(\boldsymbol{X}^\top\boldsymbol{\beta}) - \phi(-\boldsymbol{X}^\top\boldsymbol{\beta})\right\} + S/2\left\{\phi(\boldsymbol{X}^\top\boldsymbol{\beta}) + \phi(-\boldsymbol{X}^\top\boldsymbol{\beta})\right\}\right] \\
= \; & E\left[E\left[E[\Delta/2\left\{\phi(\boldsymbol{X}^\top\boldsymbol{\beta}) - \phi(-\boldsymbol{X}^\top\boldsymbol{\beta})\right\} + S/2\left\{\phi(\boldsymbol{X}^\top\boldsymbol{\beta}) + \phi(-\boldsymbol{X}^\top\boldsymbol{\beta})\right\} \mid \boldsymbol{X}^\top\boldsymbol{\beta}^{\text{opt}}\right]\right] \\
\geq \; & E\left[E(\Delta(\boldsymbol{X})/2 \mid \boldsymbol{X}^\top\boldsymbol{\beta}^{\text{opt}})E\left[\phi(\boldsymbol{X}^\top\boldsymbol{\beta}) - \phi(-\boldsymbol{X}^\top\boldsymbol{\beta}) \mid \boldsymbol{X}^\top\boldsymbol{\beta}^{\text{opt}}\right]\right. \\
& \left. + E\left[S(\boldsymbol{X})/2 \mid \boldsymbol{X}^\top\boldsymbol{\beta}^{\text{opt}}\right]E\left[\phi(\boldsymbol{X}^\top\boldsymbol{\beta}) + \phi(-\boldsymbol{X}^\top\boldsymbol{\beta}) \mid \boldsymbol{X}^\top\boldsymbol{\beta}^{\text{opt}}\right]\right)\right] \\
= \; & E\left[E(\Omega_+ \mid \boldsymbol{X}^\top\boldsymbol{\beta}^{\text{opt}})E\left[\phi\left(\boldsymbol{X}^\top\boldsymbol{\beta}\right) \mid \boldsymbol{X}^\top\boldsymbol{\beta}^{\text{opt}}\right]\right. \\
& \left. + E(\Omega_- \mid \boldsymbol{X}^\top\boldsymbol{\beta}^{\text{opt}})E\left[\phi\left(-\boldsymbol{X}^\top\boldsymbol{\beta}\right) \mid \boldsymbol{X}^\top\boldsymbol{\beta}^{\text{opt}}\right]\right] \\
\geq \; & E\left[E(\Omega_+ \mid \boldsymbol{X}^\top\boldsymbol{\beta}^{\text{opt}})\phi\left(\boldsymbol{P}^\top\boldsymbol{\beta}\boldsymbol{X}^\top\boldsymbol{\beta}^{\text{opt}}\right) + E(\Omega_- \mid \boldsymbol{X}^\top\boldsymbol{\beta}^{\text{opt}})\phi\left(-\boldsymbol{P}^\top\boldsymbol{\beta}\boldsymbol{X}^\top\boldsymbol{\beta}^{\text{opt}}\right)\right] \\
= \; & E\left[E(\Omega_+ \mid \boldsymbol{X})\phi\left(\boldsymbol{P}^\top\boldsymbol{\beta}\boldsymbol{X}^\top\boldsymbol{\beta}^{\text{opt}}\right) + \mathrm{E}(\Omega_- \mid \boldsymbol{X})\phi\left(-\boldsymbol{P}^\top\boldsymbol{\beta}\boldsymbol{X}^\top\boldsymbol{\beta}^{\text{opt}}\right)\right].
\end{aligned}
$$

The first inequality comes from Condition (a). The last equality comes from the convexity of $\phi(\cdot)$ and Condition (b). Next, we notice that

$$\begin{aligned} & E\left[E(\Omega_+ \mid \boldsymbol{X})\phi\left(\boldsymbol{P}^\top\boldsymbol{\beta}\boldsymbol{X}^\top\boldsymbol{\beta}^{\text{opt}}\right) + E(\Omega_- \mid \boldsymbol{X})\phi\left(-\boldsymbol{P}^\top\boldsymbol{\beta}\boldsymbol{X}^\top\boldsymbol{\beta}^{\text{opt}}\right)\right] \\ & -E\left[E(\Omega_+ \mid \boldsymbol{X})\phi\left(-\boldsymbol{P}^\top\boldsymbol{\beta}\boldsymbol{X}^\top\boldsymbol{\beta}^{\text{opt}}\right) + E(\Omega_- \mid \boldsymbol{X})\phi\left(\boldsymbol{P}^\top\boldsymbol{\beta}\boldsymbol{X}^\top\boldsymbol{\beta}^{\text{opt}}\right)\right] \\ = \; & E\left[\{E(\Omega_+ \mid \boldsymbol{X}) - E(\Omega_- \mid \boldsymbol{X})\}\left\{\phi\left(\boldsymbol{P}^\top\boldsymbol{\beta}\boldsymbol{X}^\top\boldsymbol{\beta}^{\text{opt}}\right) - \phi\left(-\boldsymbol{P}^\top\boldsymbol{\beta}\boldsymbol{X}^\top\boldsymbol{\beta}^{\text{opt}}\right)\right\}\right] \\ = \; & E\left[\{Q(1;\boldsymbol{X}) - Q(-1;\boldsymbol{X})\}\left\{\phi\left(\boldsymbol{P}^\top\boldsymbol{\beta}\boldsymbol{X}^\top\boldsymbol{\beta}^{\text{opt}}\right) - \phi\left(-\boldsymbol{P}^\top\boldsymbol{\beta}\boldsymbol{X}^\top\boldsymbol{\beta}^{\text{opt}}\right)\right\}\right] \end{aligned}$$

If $\boldsymbol{P}^\top\boldsymbol{\beta} > 0$, we have

$$\begin{aligned} & E\left[E(\Omega_+ \mid \boldsymbol{X})\phi\left(\boldsymbol{P}^\top\boldsymbol{\beta}\boldsymbol{X}^\top\boldsymbol{\beta}^{\text{opt}}\right) + E(\Omega_- \mid \boldsymbol{X})\phi\left(-\boldsymbol{P}^\top\boldsymbol{\beta}\boldsymbol{X}^\top\boldsymbol{\beta}^{\text{opt}}\right)\right] \\ \leq \; & E\left[E(\Omega_+ \mid \boldsymbol{X})\phi\left(-\boldsymbol{P}^\top\boldsymbol{\beta}\boldsymbol{X}^\top\boldsymbol{\beta}^{\text{opt}}\right) + E(\Omega_- \mid \boldsymbol{X})\phi\left(\boldsymbol{P}^\top\boldsymbol{\beta}\boldsymbol{X}^\top\boldsymbol{\beta}^{\text{opt}}\right)\right]. \end{aligned}$$

If $\boldsymbol{P}^\top\boldsymbol{\beta} \leq 0$, we have

$$\begin{aligned} & E\left[E(\Omega_+ \mid \boldsymbol{X})\phi\left(\boldsymbol{P}^\top\boldsymbol{\beta}\boldsymbol{X}^\top\boldsymbol{\beta}^{\text{opt}}\right) + E(\Omega_- \mid \boldsymbol{X})\phi\left(-\boldsymbol{P}^\top\boldsymbol{\beta}\boldsymbol{X}^\top\boldsymbol{\beta}^{\text{opt}}\right)\right] \\ \geq \; & E\left[E(\Omega_+ \mid \boldsymbol{X})\phi\left(-\boldsymbol{P}^\top\boldsymbol{\beta}\boldsymbol{X}^\top\boldsymbol{\beta}^{\text{opt}}\right) + E(\Omega_- \mid \boldsymbol{X})\phi\left(\boldsymbol{P}^\top\boldsymbol{\beta}\boldsymbol{X}^\top\boldsymbol{\beta}^{\text{opt}}\right)\right]. \end{aligned}$$

Combining this inequality with the inequality above, we have for $\boldsymbol{\beta} = \boldsymbol{\beta}^*$,

$$\begin{aligned} & E\left[E(\Omega_+ \mid \boldsymbol{X})\phi\left(\boldsymbol{X}^\top\boldsymbol{\beta}\right) + E(\Omega_- \mid \boldsymbol{X})\phi\left(-\boldsymbol{X}^\top\boldsymbol{\beta}\right)\right] \\ \geq \; & E\left[E(\Omega_+ \mid \boldsymbol{X})\phi\left(|\boldsymbol{P}^\top\boldsymbol{\beta}|\boldsymbol{X}^\top\boldsymbol{\beta}^{\text{opt}}\right) + E(\Omega_- \mid \boldsymbol{X})\phi\left(-|\boldsymbol{P}^\top\boldsymbol{\beta}|\boldsymbol{X}^\top\boldsymbol{\beta}^{\text{opt}}\right)\right] \end{aligned}$$

Notice the fact that $\boldsymbol{\beta}^*$ minimizes

$$E\left[E(\Omega_+ \mid \boldsymbol{X})\phi\left(\boldsymbol{X}^\top\boldsymbol{\beta}\right) + E(\Omega_- \mid \boldsymbol{X})\phi\left(-\boldsymbol{X}^\top\boldsymbol{\beta}\right)\right].$$

By the strict convexity of ϕ, we have $\boldsymbol{\beta}^* = |\boldsymbol{P}^\top\boldsymbol{\beta}^*|\boldsymbol{\beta}^{\text{opt}}$. This concludes the proof.

Next, we provide a proof for our remark. Notice that $\boldsymbol{\beta}^*$ solves

$$E\left[\left\{E(\Omega_+ \mid \boldsymbol{X})\phi'\left(\boldsymbol{X}^\top\boldsymbol{\beta}\right) - E(\Omega_- \mid \boldsymbol{X})\phi'\left(-\boldsymbol{X}^\top\boldsymbol{\beta}\right)\right\}\boldsymbol{X}\right] = 0.$$

Take the logistic loss as an example where $\phi'(t) = -\exp(-t)/(1+\exp(-t))$. We will show that

$$E[\Omega_+ \mid \boldsymbol{X}]\phi'\left\{\boldsymbol{X}^\top\boldsymbol{\beta}^{\text{opt}}\right\} - E[\Omega_- \mid \boldsymbol{X}]\phi'\left\{-\boldsymbol{X}^\top\boldsymbol{\beta}^{\text{opt}}\right\} = 0.$$

From the equation in the remark, we have that

$$\begin{aligned} & (Q(1;\boldsymbol{X}) - Q(-1;\boldsymbol{X}))/(Q(1;\boldsymbol{X}) + Q(-1;\boldsymbol{X})) \\ = \; & (\phi'(\boldsymbol{X}^\top\boldsymbol{\beta}^{\text{opt}}) - \phi'(-\boldsymbol{X}^\top\boldsymbol{\beta}^{\text{opt}}))/(\phi'(\boldsymbol{X}^\top\boldsymbol{\beta}^{\text{opt}}) + \phi'(-\boldsymbol{X}^\top\boldsymbol{\beta}^{\text{opt}})). \end{aligned}$$

Notice that $Q(1;\boldsymbol{X}) - Q(-1;\boldsymbol{X}) = E(\Omega_+ \mid \boldsymbol{X}) - E(\Omega_- \mid \boldsymbol{X})$ and $Q(1;\boldsymbol{X}) + Q(-1;\boldsymbol{X}) = E(\Omega_+ \mid \boldsymbol{X}) + E(\Omega_- \mid \boldsymbol{X})$. As such, we have

$$E(\Omega_+ \mid \boldsymbol{X})\phi^{'}\left(\boldsymbol{X}^\top\boldsymbol{\beta}^{\rm opt}\right) - E(\Omega_- \mid \boldsymbol{X})\phi^{'}\left(-\boldsymbol{X}^\top\boldsymbol{\beta}^{\rm opt}\right) = 0,$$

and

$$E\left[\left\{E(\Omega_+ \mid \boldsymbol{X})\phi^{'}\left(\boldsymbol{X}^\top\boldsymbol{\beta}^{\rm opt}\right) - E(\Omega_- \mid \boldsymbol{X})\phi^{'}\left(-\boldsymbol{X}^\top\boldsymbol{\beta}^{\rm opt}\right)\right\}\boldsymbol{X}\right] = 0.$$

By the strict convexity of ϕ, we have $\boldsymbol{\beta}^* = \boldsymbol{\beta}^{\rm opt}$.

At last, we claim that when $Y(a)$'s are bounded, we can shift $Y(a)$ by a constant such that $E(|W_a| \mid \boldsymbol{X}) = Q(a;\boldsymbol{X})$. Take $a = 1$ as an example.

$$\begin{aligned}
& E(|W_1| \mid \boldsymbol{X}) \\
= \; & E\left[\pi^{-1}(1;\boldsymbol{X})\left|YI\{A=1\} - [I\{A=1\} - \pi(1;\boldsymbol{X})]\,Q(1;\boldsymbol{X})\right| \mid \boldsymbol{X}\right] \\
= \; & E\left[\left|(Y(1) - Q(1;\boldsymbol{X}))I\{A=1\}/\pi(1;\boldsymbol{X}) + Q(1;\boldsymbol{X})\right| \mid \boldsymbol{X}\right] \\
= \; & E\left[|Q(1;\boldsymbol{X})| \mid \boldsymbol{X}\right]\pi(-1;\boldsymbol{X}) + E\left[\left|\{Y(1) - Q(1;X)\}/\pi(1;\boldsymbol{X}) + Q(1;\boldsymbol{X})\right| \mid \boldsymbol{X}\right]\pi(1;\boldsymbol{X})
\end{aligned}$$

Now, we shift $Y(a)$ by a constant and notice that $Y(1) - Q(1;\boldsymbol{X})$ does not change under any constant shift. With a sufficiently large shift, we can guarantee that $Q(1;\boldsymbol{X}) \geq 0$ and $\{Y(1) - Q(1;\boldsymbol{X})\}/\pi(1;\boldsymbol{X}) + Q(1;\boldsymbol{X}) \geq 0$. Thus, with a sufficiently large shift on the outcome, we can achieve $E(|W_a| \mid \boldsymbol{X}) = Q(a;\boldsymbol{X})$. □

In the following, we show two non-trivial examples where Condition (a) in Lemma 1 is satisfied.

Example 1 *If X_j's are independent with mean 0, let $\mathcal{A} = \left\{j \in \mathbb{N} : \beta_j^{\rm opt} \neq 0\right\}$. Consider the following model*

$$E(Y(a) \mid \boldsymbol{X}) = f_a(\boldsymbol{X}^\top\boldsymbol{\beta}^{\rm opt}) + g(\boldsymbol{X}_{\mathcal{A}^c}).$$

Define

$$\mathcal{G} = \left\{g(\boldsymbol{X}_{\mathcal{A}^c}) : E(g(\boldsymbol{X}_{\mathcal{A}^c})X_j) = 0, E(g(\boldsymbol{X}_{\mathcal{A}^c})) = 0, \forall j \in \mathcal{A}^c\right\}.$$

Then if $g(\boldsymbol{X}_{\mathcal{A}^c}) \in \mathcal{G}$, then Condition (a) is satisfied for the model above.

Proof. First, we have

$$E(Y(1) \mid \boldsymbol{X}) - E(Y(-1) \mid \boldsymbol{X}) = f_1(\boldsymbol{X}^\top\boldsymbol{\beta}^{\rm opt}) - f_{-1}(\boldsymbol{X}^\top\boldsymbol{\beta}^{\rm opt}) \in \Delta_\phi(\boldsymbol{\beta}),$$

for any $\boldsymbol{\beta}$. Next, we will show that $E(Y(1) \mid \boldsymbol{X}) + E(Y(-1) \mid \boldsymbol{X}) \in S_\phi(\boldsymbol{\beta}^*)$. Because $f_1(\boldsymbol{X}^\top\boldsymbol{\beta}^{\rm opt}) + f_{-1}(\boldsymbol{X}^\top\boldsymbol{\beta}^{\rm opt}) \in S_\phi(\boldsymbol{\beta})$ for all β, we just need to verify

$$\begin{aligned}
& E\left[g(\boldsymbol{X}_{\mathcal{A}^c})\left\{\phi(\boldsymbol{X}^\top\boldsymbol{\beta}^*) + \phi(-\boldsymbol{X}^\top\boldsymbol{\beta}^*)\right\} \mid \boldsymbol{X}^\top\boldsymbol{\beta}^{\rm opt}\right] \\
- \; & E\left[g(\boldsymbol{X}_{\mathcal{A}^c}) \mid \boldsymbol{X}^\top\boldsymbol{\beta}^{\rm opt}\right]E\left[\phi(\boldsymbol{X}^\top\boldsymbol{\beta}^*) + \phi(-\boldsymbol{X}^\top\boldsymbol{\beta}^*) \mid \boldsymbol{X}^\top\boldsymbol{\beta}^{\rm opt}\right] = 0.
\end{aligned}$$

Notice that $E\left[g(\boldsymbol{X}_{\mathcal{A}^c}) \mid \boldsymbol{X}^\top\boldsymbol{\beta}^{\rm opt}\right] = E\left[g(\boldsymbol{X}_{\mathcal{A}^c})\right] = 0$, we just need to show that

$$E\left[g(\boldsymbol{X}_{\mathcal{A}^c})\left\{\phi(\boldsymbol{X}^\top\boldsymbol{\beta}^*) + \phi(-\boldsymbol{X}^\top\boldsymbol{\beta}^*)\right\} \mid \boldsymbol{X}^\top\boldsymbol{\beta}^{\rm opt}\right] = 0.$$

Define $\mathcal{A}^* = \left\{j \in \mathbb{N} : \beta_j^* \neq 0\right\}$. This is equivalent to show that

$$\begin{aligned} 0 &= E\left[g(\boldsymbol{X}_{\mathcal{A}^c})\left\{\phi(\boldsymbol{X}^\top\boldsymbol{\beta}^*) + \phi(-\boldsymbol{X}^\top\boldsymbol{\beta}^*)\right\} \mid \boldsymbol{X}^\top\boldsymbol{\beta}^{\mathrm{opt}}\right] \\ &= E\left[g(\boldsymbol{X}_{\mathcal{A}^c})\left\{\phi(\boldsymbol{X}_{\mathcal{A}^*}^\top\boldsymbol{\beta}_{\mathcal{A}^*}^*) + \phi(-\boldsymbol{X}_{\mathcal{A}^*}^\top\boldsymbol{\beta}_{\mathcal{A}^*}^*)\right\} \mid \boldsymbol{X}_{\mathcal{A}}^\top\boldsymbol{\beta}_{\mathcal{A}}^{\mathrm{opt}}\right]. \end{aligned}$$

This is satisfied if $\mathcal{A}^* \subset \mathcal{A}$ due to $E[g(\boldsymbol{X}_{\mathcal{A}^c})] = 0$. To show this, assume W_1 and W_{-1} are positive, we consider the optimization problem

$$E[E(Y(1) \mid \boldsymbol{X})\phi(\boldsymbol{X}_{\mathcal{A}}^\top\boldsymbol{\beta}_{\mathcal{A}}) + E(Y(-1) \mid \boldsymbol{X})\phi(-\boldsymbol{X}_{\mathcal{A}}^\top\boldsymbol{\beta}_{\mathcal{A}})].$$

Due to the strictly convexity, we can find the unique minimizer of this optimization problem and denote it as $\boldsymbol{\beta}_{\mathcal{A}}^*$. The $\boldsymbol{\beta}_{\mathcal{A}}^*$ satisfies that

$$E[\{E(Y(1) \mid \boldsymbol{X})\phi^{'}(\boldsymbol{X}_{\mathcal{A}}^\top\boldsymbol{\beta}_{\mathcal{A}}^*) - E(Y(-1) \mid \boldsymbol{X})\phi^{'}(-\boldsymbol{X}_{\mathcal{A}}^\top\boldsymbol{\beta}_{\mathcal{A}}^*)\}X_j],$$

for all $j \in \mathcal{A}$ due to the first-order condition. Now, we claim that $\boldsymbol{\beta}^* = (\boldsymbol{\beta}_{\mathcal{A}}^*, 0)^\top$. By first order condition, we just need to show that

$$E[\{E(Y(1) \mid \boldsymbol{X})\phi^{'}(\boldsymbol{X}_{\mathcal{A}}^\top\boldsymbol{\beta}_{\mathcal{A}}^*) - E(Y(-1) \mid \boldsymbol{X})\phi^{'}(-\boldsymbol{X}_{\mathcal{A}}^\top\boldsymbol{\beta}_{\mathcal{A}}^*)\}X_j] = 0,$$

for all $j \in \mathcal{A}^c$. Notice that

$$\begin{aligned} & E[\{E(Y(1) \mid \boldsymbol{X})\phi^{'}(\boldsymbol{X}_{\mathcal{A}}^\top\boldsymbol{\beta}_{\mathcal{A}}^*) - E(Y(-1) \mid \boldsymbol{X})\phi^{'}(-\boldsymbol{X}_{\mathcal{A}}^\top\boldsymbol{\beta}_{\mathcal{A}}^*)\}X_j] \\ = & E[\{f_1(\boldsymbol{X}^\top\boldsymbol{\beta}^{\mathrm{opt}})\phi^{'}(\boldsymbol{X}_{\mathcal{A}}^\top\boldsymbol{\beta}_{\mathcal{A}}^*) - f_{-1}(\boldsymbol{X}^\top\boldsymbol{\beta}^{\mathrm{opt}})\phi^{'}(-\boldsymbol{X}_{\mathcal{A}}^\top\boldsymbol{\beta}_{\mathcal{A}}^*)\}X_j] \\ & +E[\{g(\boldsymbol{X}_{\mathcal{A}^c})\phi^{'}(\boldsymbol{X}_{\mathcal{A}}^\top\boldsymbol{\beta}_{\mathcal{A}}^*) - g(\boldsymbol{X}_{\mathcal{A}^c})\phi^{'}(-\boldsymbol{X}_{\mathcal{A}}^\top\boldsymbol{\beta}_{\mathcal{A}}^*)\}X_j] \\ = & E[f_1(\boldsymbol{X}^\top\boldsymbol{\beta}^{\mathrm{opt}})\phi^{'}(\boldsymbol{X}_{\mathcal{A}}^\top\boldsymbol{\beta}_{\mathcal{A}}^*) - f_{-1}(\boldsymbol{X}^\top\boldsymbol{\beta}^{\mathrm{opt}})\phi^{'}(-\boldsymbol{X}_{\mathcal{A}}^\top\boldsymbol{\beta}_{\mathcal{A}}^*)]E[X_j] \\ & +E[\phi^{'}(\boldsymbol{X}_{\mathcal{A}}^\top\boldsymbol{\beta}_{\mathcal{A}}^*) - \phi^{'}(-\boldsymbol{X}_{\mathcal{A}}^\top\boldsymbol{\beta}_{\mathcal{A}}^*)]E[g(\boldsymbol{X}_{\mathcal{A}^c})X_j] \\ = & 0. \end{aligned}$$

Thus, we have that $\boldsymbol{\beta}^* = (\boldsymbol{\beta}_{\mathcal{A}}^*, 0)^\top$, which implies that $\mathcal{A}^* \subset \mathcal{A}$. $\square$

Example 2 *If $\boldsymbol{\beta}^{\mathrm{opt}} = e_1$, $\boldsymbol{X} \sim N(0, \boldsymbol{I})$, and ϕ is a logistics loss, define*

$$\mathcal{A} = \left\{j \in \mathbb{N} : \beta_j^{\mathrm{opt}} \neq 0\right\},$$

and

$$\mathcal{F} = \left\{f(\boldsymbol{X}_{\mathcal{A}^c}) \in R_+ : \mathrm{cov}\left(f(\boldsymbol{X}_{\mathcal{A}^c}), \boldsymbol{X} \mid \boldsymbol{X}_1\right) = 0 \text{ and } E(f(\boldsymbol{X}_{\mathcal{A}^c})\boldsymbol{X}_{\mathcal{A}^c}) = 0\right\}.$$

Consider the following model

$$E(Y(a) \mid \boldsymbol{X}) = f_a(\boldsymbol{X}^\top\boldsymbol{\beta}^{\mathrm{opt}})f(\boldsymbol{X}_{\mathcal{A}^c}) + g(\boldsymbol{X}_{\mathcal{A}^c}).$$

then if $g(\boldsymbol{X}_{\mathcal{A}^c}) \in \mathcal{G}$ and $f(\boldsymbol{X}_{\mathcal{A}^c}) \in \mathcal{F}$, then Condition (a) is satisfied for the model above. Especially, all even polynomials $B_l(\boldsymbol{X}_{\mathcal{A}^c}) = \prod_{j \in \boldsymbol{X}_{\mathcal{A}^c}} X_j^{2k_j}$ belongs to $\mathcal{F}$, where $k_j \in N$.

Proof. Let

$$\begin{aligned}
& C(\boldsymbol{\beta}; f) \\
= \ & \text{cov}\left[E(Y(1) \mid \boldsymbol{X}) - E(Y(-1) \mid \boldsymbol{X}), \phi(\boldsymbol{X}^\top \boldsymbol{\beta}) - \phi(-\boldsymbol{X}^\top \boldsymbol{\beta}) \mid \boldsymbol{X}^\top \boldsymbol{\beta}^{\text{opt}}\right] \\
= \ & \text{cov}\left[(f_1(\boldsymbol{X}^\top \boldsymbol{\beta}^{\text{opt}}) - f_{-1}(\boldsymbol{X}^\top \boldsymbol{\beta}^{\text{opt}}))f(\boldsymbol{X}_{\mathcal{A}^c}), \phi(\boldsymbol{X}^\top \boldsymbol{\beta}) - \phi(-\boldsymbol{X}^\top \boldsymbol{\beta}) \mid \boldsymbol{X}^\top \boldsymbol{\beta}^{\text{opt}}\right] \\
= \ & (f_1(\boldsymbol{X}^\top \boldsymbol{\beta}^{\text{opt}}) - f_{-1}(\boldsymbol{X}^\top \boldsymbol{\beta}^{\text{opt}}))\text{cov}\left[f(\boldsymbol{X}_{\mathcal{A}^c}), \phi(\boldsymbol{X}^\top \boldsymbol{\beta}) - \phi(-\boldsymbol{X}^\top \boldsymbol{\beta}) \mid \boldsymbol{X}^\top \boldsymbol{\beta}^{\text{opt}}\right].
\end{aligned}$$

We just need to show that

$$\text{cov}\left[f(\boldsymbol{X}_{\mathcal{A}^c}), \phi(\boldsymbol{X}^\top \boldsymbol{\beta}) - \phi(-\boldsymbol{X}^\top \boldsymbol{\beta}) \mid \boldsymbol{X}^\top \boldsymbol{\beta}^{\text{opt}}\right] = 0.$$

The gradient of $C(\boldsymbol{\beta}; f)$ is proportional to $\text{cov}\left(f(\boldsymbol{X}_{\mathcal{A}^c}), \boldsymbol{X} \mid X_1\right)$. Because

$$\text{cov}\left(f(\boldsymbol{X}_{\mathcal{A}^c}), \boldsymbol{X} \mid X_1\right) = 0,$$

we can then conclude that $C(\boldsymbol{\beta}; f) = 0$ for all $\boldsymbol{\beta}$. Thus, we have that

$$E(Y(1) \mid \boldsymbol{X}) - E(Y(-1) \mid \boldsymbol{X}) \in \Delta_\phi(\boldsymbol{\beta}^*).$$

Next, we will verify that

$$E(Y(1) \mid \boldsymbol{X}) + E(Y(-1) \mid \boldsymbol{X}) \in S_\phi(\boldsymbol{\beta}^*).$$

Similar to Example 1, we will show that $\mathcal{A}^* \subset \mathcal{A}$. To show this, assume W_1 and W_{-1} are positive, we consider the optimization problem

$$E[E(Y(1) \mid \boldsymbol{X})\phi(\boldsymbol{X}_{\mathcal{A}}^\top \boldsymbol{\beta}_{\mathcal{A}}) + E(Y(-1) \mid \boldsymbol{X})\phi(-\boldsymbol{X}_{\mathcal{A}}^\top \boldsymbol{\beta}_{\mathcal{A}})].$$

Due to the strictly convexity, we can find the unique minimizer of this optimization problem and denote it as $\boldsymbol{\beta}_{\mathcal{A}}^*$. The $\boldsymbol{\beta}_{\mathcal{A}}^*$ satisfies that

$$E[\{E(Y(1) \mid \boldsymbol{X})\phi^{'}(\boldsymbol{X}_{\mathcal{A}}^\top \boldsymbol{\beta}_{\mathcal{A}}^*) - E(Y(-1) \mid \boldsymbol{X})\phi^{'}(-\boldsymbol{X}_{\mathcal{A}}^\top \boldsymbol{\beta}_{\mathcal{A}}^*)\}X_j],$$

for all $j \in \mathcal{A}$ due to the first-order condition. Now, we claim that $\boldsymbol{\beta}^* = (\boldsymbol{\beta}_{\mathcal{A}}^*, 0)^\top$. By first order condition, we just need to show that

$$E[\{E(Y(1) \mid \boldsymbol{X})\phi^{'}(\boldsymbol{X}_{\mathcal{A}}^\top \boldsymbol{\beta}_{\mathcal{A}}^*) - E(Y(-1) \mid \boldsymbol{X})\phi^{'}(-\boldsymbol{X}_{\mathcal{A}}^\top \boldsymbol{\beta}_{\mathcal{A}}^*)\}X_j] = 0,$$

for all $j \in \mathcal{A}^c$. Notice that

$$\begin{aligned}
& E[\{E(Y(1) \mid \boldsymbol{X})\phi^{'}(\boldsymbol{X}_{\mathcal{A}}^\top \boldsymbol{\beta}_{\mathcal{A}}^*) - E(Y(-1) \mid \boldsymbol{X})\phi^{'}(-\boldsymbol{X}_{\mathcal{A}}^\top \boldsymbol{\beta}_{\mathcal{A}}^*)\}X_j] \\
= \ & E[\{f_1(\boldsymbol{X}^\top \boldsymbol{\beta}^{\text{opt}})f(\boldsymbol{X}_{\mathcal{A}^c})\phi^{'}(\boldsymbol{X}_{\mathcal{A}}^\top \boldsymbol{\beta}_{\mathcal{A}}^*) - f_{-1}(\boldsymbol{X}^\top \boldsymbol{\beta}^{\text{opt}})f(\boldsymbol{X}_{\mathcal{A}^c})\phi^{'}(-\boldsymbol{X}_{\mathcal{A}}^\top \boldsymbol{\beta}_{\mathcal{A}}^*)\}X_j] \\
& + E[\{g(\boldsymbol{X}_{\mathcal{A}^c})\phi^{'}(\boldsymbol{X}_{\mathcal{A}}^\top \boldsymbol{\beta}_{\mathcal{A}}^*) - g(\boldsymbol{X}_{\mathcal{A}^c})\phi^{'}(-\boldsymbol{X}_{\mathcal{A}}^\top \boldsymbol{\beta}_{\mathcal{A}}^*)\}X_j] \\
= \ & E[f_1(\boldsymbol{X}^\top \boldsymbol{\beta}^{\text{opt}})\phi^{'}(\boldsymbol{X}_{\mathcal{A}}^\top \boldsymbol{\beta}_{\mathcal{A}}^*) - f_{-1}(\boldsymbol{X}^\top \boldsymbol{\beta}^{\text{opt}})\phi^{'}(-\boldsymbol{X}_{\mathcal{A}}^\top \boldsymbol{\beta}_{\mathcal{A}}^*)]E[f(\boldsymbol{X}_{\mathcal{A}^c})X_j] \\
& + E[\phi^{'}(\boldsymbol{X}_{\mathcal{A}}^\top \boldsymbol{\beta}_{\mathcal{A}}^*) - \phi^{'}(-\boldsymbol{X}_{\mathcal{A}}^\top \boldsymbol{\beta}_{\mathcal{A}}^*)]E[g(\boldsymbol{X}_{\mathcal{A}^c})X_j] \\
= \ & 0.
\end{aligned}$$

Thus, we have that $\boldsymbol{\beta}^* = (\boldsymbol{\beta}^*_{\mathcal{A}}, 0)^\top$, which implies that $\mathcal{A}^* \subset \mathcal{A}$. Thus, we have that

$$\begin{aligned} & E\left[g(\boldsymbol{X}_{\mathcal{A}^c})\left\{\phi(\boldsymbol{X}^\top\boldsymbol{\beta}^*) + \phi(-\boldsymbol{X}^\top\boldsymbol{\beta}^*)\right\} \mid \boldsymbol{X}^\top\boldsymbol{\beta}^{\text{opt}}\right] \\ - \quad & E(g(\boldsymbol{X}_{\mathcal{A}^c}) \mid \boldsymbol{X}^\top\boldsymbol{\beta}^{\text{opt}})E(\phi(\boldsymbol{X}^\top\boldsymbol{\beta}^*) + \phi(-\boldsymbol{X}^\top\boldsymbol{\beta}^*) \mid \boldsymbol{X}^\top\boldsymbol{\beta}^{\text{opt}}) = 0, \end{aligned}$$

and

$$\begin{aligned} & E\left[(f_1(\boldsymbol{X}^\top\boldsymbol{\beta}^{\text{opt}}) + f_{-1}(\boldsymbol{X}^\top\boldsymbol{\beta}^{\text{opt}}))f(\boldsymbol{X}_{\mathcal{A}^c})\left\{\phi(\boldsymbol{X}^\top\boldsymbol{\beta}^*) + \phi(-\boldsymbol{X}^\top\boldsymbol{\beta}^*)\right\} \mid \boldsymbol{X}^\top\boldsymbol{\beta}^{\text{opt}}\right] \\ - \quad & E[(f_1(\boldsymbol{X}^\top\boldsymbol{\beta}^{\text{opt}}) + f_{-1}(\boldsymbol{X}^\top\boldsymbol{\beta}^{\text{opt}}))f(\boldsymbol{X}_{\mathcal{A}^c}) \mid \boldsymbol{X}^\top\boldsymbol{\beta}^{\text{opt}}] \\ \times \quad & E(\phi(\boldsymbol{X}^\top\boldsymbol{\beta}^*) + \phi(-\boldsymbol{X}^\top\boldsymbol{\beta}^*) \mid \boldsymbol{X}^\top\boldsymbol{\beta}^{\text{opt}}) = 0. \end{aligned}$$

Thus, we have that

$$E(Y(1) \mid \boldsymbol{X}) + E(Y(-1) \mid \boldsymbol{X}) \in S_\phi(\boldsymbol{\beta}^*).$$

At the end, we will verify that $\operatorname{cov}(B_l(\boldsymbol{X}_{\mathcal{A}^c}), \boldsymbol{X} \mid \boldsymbol{X}_1) = 0$ and $E(f(\boldsymbol{X}_{\mathcal{A}^c})\boldsymbol{X}_{\mathcal{A}^c}) = 0$. When $j \neq 1$, we have

$$\operatorname{cov}(B_l(\boldsymbol{X}_{\mathcal{A}^c}), X_j \mid \boldsymbol{X}_1) = E(B_l(\boldsymbol{X}_{\mathcal{A}^c})X_j \mid \boldsymbol{X}_1) = 0,$$

by $E(X_j \mid X_1) = E(X_j) = 0$ and the distribution of X_j is symmetric. When $j = 1$, we also have that $\operatorname{cov}(B_l(\boldsymbol{X}_{\mathcal{A}^c}), X_1 \mid X_1) = 0$ by the definition of conditional expectation. This concludes that $B_l(\boldsymbol{X}_{\mathcal{A}^c}) = \prod_{j\in\mathcal{A}^c} X_j^{2k_j} \in \mathcal{F}$. $\square$

Appendix C.

In Appendix C, we compared the list of significant covariates selected by the proposed method and Q-learning. The proposed approach identified most of the covariates selected by Q-learning. In addition, the proposed approach also identifies 10 new driving factors, which provide additional insights for further investigations. The figure 12 shows the Venn plot of the selected covariates. The 95%-confidence interval of the selected covariates by the proposed method is reported in Table 3.

Appendix D.

In Appendix D, we study the limiting property of the proposed method. We show the proof of Theorems 3 - 7. In addition, we also show the validity of the Algorithm 4, where the nuisance parameters are estimated nonparametrically and there is no sample splitting procedure.

Theorem 8 shows that Algorithm 2, where there is no sample-splitting procedure, is valid when the nuisance parameters are estimated parametrically.

Theorem 8 *Assume that $\boldsymbol{X}$ is bounded. Suppose that $\pi(a;\boldsymbol{X})$ and $Q(a;\boldsymbol{X})$ are known to follow parametric models $\pi(a;\boldsymbol{X},\boldsymbol{\beta}_\pi)$ and $Q(a;\boldsymbol{X},\boldsymbol{\beta}_Q)$ with true parameters $\boldsymbol{\beta}^*_\pi$ and $\boldsymbol{\beta}^*_Q$ respectively. Assume $\pi(a;\boldsymbol{X},\boldsymbol{\beta}_\pi)$ and $Q(a;\boldsymbol{X},\boldsymbol{\beta}_Q)$ are second order continuously differentiable, and $\|\nabla_{\boldsymbol{\beta}_\pi}\pi(a;\boldsymbol{X},\boldsymbol{\beta}^*_\pi)\|_\infty$ and $\left\|\nabla_{\boldsymbol{\beta}_Q}Q(a;\boldsymbol{X},\boldsymbol{\beta}^*_Q)\right\|_\infty$ are bounded. Further, there*

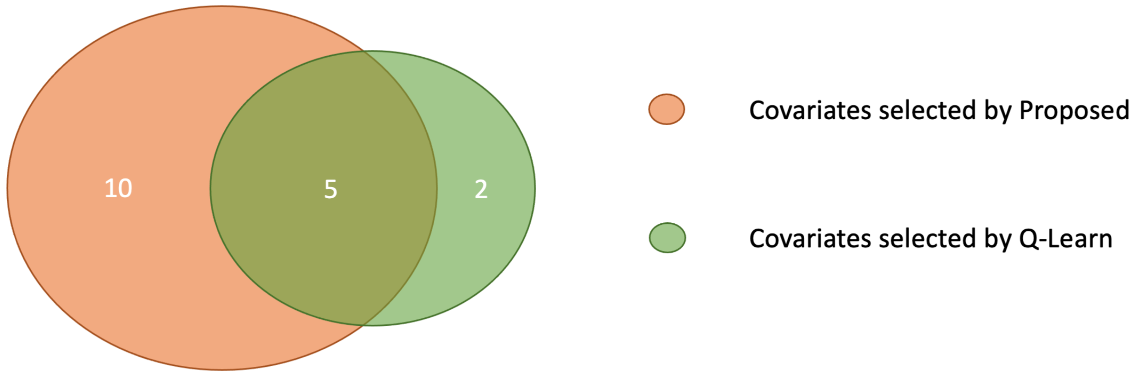

Figure 12: Variable selection using the proposed method and Q-learning.

Algorithm 4: Inference of $\boldsymbol{\beta}^*$ with parametric propensity and outcome model estimations

---

Input: n samples.

Output: $\widehat{\beta}$ and a p-value for $\mathcal{H}_0 : \boldsymbol{\beta}_1^* = 0$.

Use all data to fit a parametric regression model with a lasso penalty and obtain an estimator $\widehat{\pi}$ for the propensity and an estimator $\widehat{Q}$ for the outcome model;

Obtain the proposed estimator $\widehat{\beta}$ by $\min_\beta E_n \left[l_\phi \left(\beta; \widehat{\Omega}_+, \widehat{\Omega}_- \right) \right] + \lambda_n \|\beta\|_1$, where $\widehat{\Omega}_+$ and $\widehat{\Omega}_-$ are computed with $\widehat{\pi}$ and $\widehat{Q}$ plugged in, and λ_n is tuned by cross-validation;

Obtain an estimator $\widehat{w}$ for w^* by $\min_w E_n \left[\nabla^2 l_\phi \left(\widehat{\beta}; \widehat{\Omega}_+, \widehat{\Omega}_- \right) \left(X_1 - X_{-1}^\top w \right)^2 \right] + \widetilde{\lambda}_n \|w\|_1$, where $\widetilde{\lambda}_n$ is tuned by cross-validation;

Let $\left(\widehat{\beta}_{\text{null}} \right)^\top = \left(0, \left(\widehat{\beta}_{-1} \right)^\top \right)$, where $\widehat{\beta}_{-1}$ is a $p-1$ dimensional sub-vector of $\widehat{\beta}$ without $\widehat{\beta}_1$. Construct the de-correlated score test statistic $S(\widehat{\beta}_{\text{null}}, \widehat{w})$ as $S \left(\widehat{\beta}_{\text{null}}, \widehat{w} \right) = E_n \left[\nabla l_\phi \left(\widehat{\beta}_{\text{null}}; \widehat{\Omega}_+, \widehat{\Omega}_- \right) \left(X_1 - X_{-1}^\top \widehat{w} \right) \right]$, and the estimator of the variance $\widehat{\sigma}^2 = E_n \left[\left\{ \nabla l_\phi \left(\widehat{\beta}_{\text{null}}; \widehat{\Omega}_+, \widehat{\Omega}_- \right) \right\}^2 \left(X_1 - X_{-1}^\top \widehat{w} \right)^2 \right]$;

Calculate the p-value by $2 \left(1 - \Phi(|S|/\widehat{\sigma}) \right)$, where $\Phi(\cdot)$ is the cumulative distribution function of a standard normal distribution.

	Coef	95% - CI
Chronic Complications : Fluid and Electrolyte Disorders	-0.024	[-0.047,-0.001]
Chronic Complications : African American	-0.027	[-0.052,-0.001]
Alcohol Abuse : Entitlement Disability	-0.054	[-0.104,-0.004]
HCC Community Score : Special Chronic Conditions	-0.022	[-0.042,-0.002]
Hypertension : Lower Extremity Ulcer	-0.036	[-0.068,-0.005]
HbA1c at Baseline : African American	0.019	[0.003,0.036]
Entitlement Disability : Hypothyroidism	-0.024	[-0.045,-0.003]
Cardiac Heart Failure : Peripheral Vascular Disease	-0.029	[-0.057, -0.001]
Chronic Kidney Disease : HbA1c at Baseline	0.081	[0.014, 0.149]
Other Race : Special Chronic Conditions	0.016	[0.003,0.029]
Liver Disease : Weight Loss	0.015	[0.003,0.027]
Other Neurological Disorders : Female	-0.021	[-0.038,-0.005]
Lower Extremity Ulcer : HbA1c at Baseline	0.039	[0.010,0.069]
Chronic Complications : Bucketized Age	0.040	[0.016,0.063]
HbA1c at Baseline : Female	0.044	[0.028,0.061]

Table 3: Coefficients and CI for the significant covariates of the estimated decision rule. Special chronic conditions refer to chronic conditions including amputation, chronic blood loss, drug abuse, lymphoma, metastatistic cancer, and peptic ulcer disease. Bucketized age refers to a variable created by bucketizing the raw age by its observed quartiles. Other Race refers to the race excluding White and Black.

exist constants C_π and C_Q such that $\nabla^2_{\boldsymbol{\beta}_\pi}\pi(a;\boldsymbol{X},\boldsymbol{\beta}_\pi) \prec C_\pi \boldsymbol{X}\boldsymbol{X}^\top$ and $\nabla^2_{\boldsymbol{\beta}_Q}Q(a;\boldsymbol{X},\boldsymbol{\beta}_Q) \prec C_Q\boldsymbol{X}\boldsymbol{X}^\top$, where for two matrices A and B, $A \prec B$ implies that $B - A$ is positive semi-definite. In addition, suppose that $\|\widehat{\boldsymbol{\beta}}_\pi - \boldsymbol{\beta}^*_\pi\|_1 = O_p(n^{-\alpha})$ and $\|\widehat{\boldsymbol{\beta}}_Q - \boldsymbol{\beta}^*_Q\|_1 = O_p(n^{-\beta})$ for some $\alpha, \beta > 0$, we require that $\alpha + \beta > 1/2$. In addition, we require that

$$R\max\{s^*, s'\}\log n(\log p)^{3/2} = o(n^{1/2})$$

and

$$(n^{-\alpha} + n^{-\beta})R \to 0,$$

where $s^* = \|\boldsymbol{\beta}^*\|_0$ and $s' = \max_j \|\boldsymbol{w}^*_j\|_0$.

Assume that Conditions (C1)-(C4) hold. For Algorithm 4, under the null hypothesis $H_0 : \beta^*_j = 0$, by choosing $\lambda_n \asymp \tilde{\lambda}_n \asymp (\log p/n)^{1/2}$, we have

$$n^{1/2}S_j \to N(0, \sigma^2),$$

and $\widehat{\sigma}^2_j \to \sigma^2_j$, where $\widehat{\sigma}^2_j$ is given in Algorithm 2, and $\sigma^2_j = \left(\boldsymbol{\nu}^*_j\right)^\top \mathrm{var}\left[\nabla^2 l_\phi(\boldsymbol{\beta}^*;\Omega_+,\Omega_-)\right]\boldsymbol{\nu}^*_j$.

For the value inference, we also have the following theorem.

Theorem 9 *Assume that* Y *is bounded and denote the sample size of* $\widetilde{I}_1$ *as* n_1 *and* $\widetilde{I}_2$ *as* n_2. *In addition to the conditions in Theorem 3, we further assume* $n_1^{-\alpha-\beta}n_2^{1/2} = o(1)$ *and one of the following conditions: 1) Conditions (C6) and (C7) holds with* $\left(s(\log p/n_1)^{1/2}\right)^{\zeta+\gamma} = o_p(n_2^{-1/2})$*; 2) Condition (C7) holds with* $P\left(\left|\boldsymbol{X}^\top\boldsymbol{\beta}^*\right| = 0\right) = 0$ *and* $\left(s(\log p/n_1)^{1/2}\right)^{\gamma} = o_p(n_2^{-1/2})$*, then we have*

$$n_2{}^{1/2}\sigma_V^{-2}(\widehat{V}(\widehat{D}) - V(D^*)) \to N(0,1),$$

where $\sigma_V^2 = \mathrm{var}\left[W_{\widehat{D}(\boldsymbol{X})}(Y, \boldsymbol{X}, A, \pi, Q)\right]$.

Under Conditions (C6) and (C7) with $\left(s(\log p/n_1)^{1/2}\right)^{\zeta+\gamma} = o_p(n_2^{-1/2})$, Theorem 7 holds for both regular and non-regular cases. Condition (C6) implicitly assumes that $\boldsymbol{\beta}^*$ corresponds to the optimal individualized treatment rule. When Condition (C6) fails, the inference of the value under $D^*(\boldsymbol{X})$ is challenging but possible if Condition (C7) holds with $P\left(\left|\boldsymbol{X}^\top\boldsymbol{\beta}^*\right| = 0\right) = 0$ and $\left(s(\log p/n_1)^{1/2}\right)^{\gamma} = o_p(n_2^{-1/2})$.

Proof of Theorems and Corollary.

The proof of Thoerem 3 can be found for the proof of Lemma 10. The following provides the proof of Theorem 4.

Proof of Thoerem 4. Let's compute the following

$$
\begin{aligned}
& (n/K)^{1/2} S_j^{(k)}\left(\widehat{\boldsymbol{\beta}}^{(k)}_{\mathrm{null(j)}}, \widehat{\boldsymbol{w}}_j^{(k)}\right) && (10)\\
= \; & (n/K)^{1/2} E_n^{(k)}\left[\left\{\widehat{\Omega}_+^{(k)}\phi'(\boldsymbol{X}^\top\widehat{\boldsymbol{\beta}}^{(k)}_{\mathrm{null(j)}}) - \widehat{\Omega}_-^{(k)}\phi'(-\boldsymbol{X}^\top\widehat{\boldsymbol{\beta}}^{(k)}_{\mathrm{null(j)}})\right\}(X_j - \boldsymbol{X}_{-j}^\top\widehat{\boldsymbol{w}}_j^{(k)})\right] && (11)\\
= \; & (n/K)^{1/2} E_n^{(k)}\left[\left\{\widehat{\Omega}_+^{(k)}\phi'(\boldsymbol{X}^\top\widehat{\boldsymbol{\beta}}^{(k)}_{\mathrm{null(j)}}) - \widehat{\Omega}_-^{(k)}\phi'(-\boldsymbol{X}^\top\widehat{\boldsymbol{\beta}}^{(k)}_{\mathrm{null(j)}})\right\}(X_j - \boldsymbol{X}_{-j}^\top\boldsymbol{w}_j^{*})\right] && (12)\\
& +(n/K)^{1/2} E_n^{(k)}\left[\left\{\widehat{\Omega}_+^{(k)}\phi'(\boldsymbol{X}^\top\widehat{\boldsymbol{\beta}}^{(k)}_{\mathrm{null(j)}}) - \widehat{\Omega}_-^{(k)}\phi'(-\boldsymbol{X}^\top\widehat{\boldsymbol{\beta}}^{(k)}_{\mathrm{null(j)}})\right\}(\boldsymbol{X}_{-j}^\top\widehat{\boldsymbol{w}}_j^{(k)} - \boldsymbol{X}_{-j}^\top\boldsymbol{w}_j^{*})\right]\\
= \; & (n/K)^{1/2} E_n^{(k)}\left[\left\{\widehat{\Omega}_+^{(k)}\phi'(\boldsymbol{X}^\top\boldsymbol{\beta}^*) - \widehat{\Omega}_-^{(k)}\phi'(-\boldsymbol{X}^\top\boldsymbol{\beta}^*)\right\}(X_j - \boldsymbol{X}_{-j}^\top\boldsymbol{w}_j^{*})\right]\\
& +(n/K)^{1/2} E_n^{(k)}\left[\left\{\widehat{\Omega}_+^{(k)}\phi''(\boldsymbol{X}^\top\boldsymbol{\beta}^*) + \widehat{\Omega}_-^{(k)}\phi''(-\boldsymbol{X}^\top\boldsymbol{\beta}^*)\right\}\boldsymbol{X}_{-j}^\top(\widehat{\boldsymbol{\beta}}^{(k)}_{-j} - \boldsymbol{\beta}^*_{-j})\right.\\
& \left.(X_j - \boldsymbol{X}_{-j}^\top\boldsymbol{w}_j^{*})\right]\\
& +(n/K)^{1/2} E_n^{(k)}\left[\left\{\widehat{\Omega}_+^{(k)}\phi'(\boldsymbol{X}^\top\boldsymbol{\beta}^*) - \widehat{\Omega}_-^{(k)}\phi'(-\boldsymbol{X}^\top\boldsymbol{\beta}^*)\right\}(X_{-j}^\top\widehat{\boldsymbol{w}}_j^{(k)} - \boldsymbol{X}_{-j}^\top\boldsymbol{w}_j^{*})\right]\\
& +o_p(1), && (13)
\end{aligned}
$$

uniformly over $j \in \{1, \cdots, J\}$. The first line from (10) to (11) is the definition of

$$S_j^{(k)}\left(\widehat{\boldsymbol{\beta}}^{(k)}_{\mathrm{null(j)}}, \widehat{\boldsymbol{w}}_j^{(k)}\right).$$

From Line (12) to (13), we use that

$$
\begin{aligned}
& E_n^{(k)}\left[\left\{\widehat{\Omega}_+^{(k)}\phi'(\boldsymbol{X}^\top\boldsymbol{\beta}^*)-\widehat{\Omega}_-^{(k)}\phi'(-\boldsymbol{X}^\top\boldsymbol{\beta}^*)\right\}(X_j-\boldsymbol{X}_{-j}^\top\boldsymbol{w}_j^*)\right]\\
= & E_n^{(k)}\left[\left\{\widehat{\Omega}_+^{(k)}\phi'(\boldsymbol{X}^\top\boldsymbol{\beta}^*)-\widehat{\Omega}_-^{(k)}\phi'(-\boldsymbol{X}^\top\boldsymbol{\beta}^*)\right\}(X_j-\boldsymbol{X}_{-j}^\top\boldsymbol{w}_j^*)\right]\\
& +E_n^{(k)}\left[\left\{\widehat{\Omega}_+^{(k)}\phi''(\boldsymbol{X}^\top\boldsymbol{\beta}_{\text{null,j}})+\widehat{\Omega}_-^{(k)}\phi''(-\boldsymbol{X}^\top\boldsymbol{\beta}_{\text{null,j}})\right\}\boldsymbol{X}_{-j}^\top(\widehat{\boldsymbol{\beta}}_{-j}^{(k)}-\boldsymbol{\beta}_{-j}^*)\right.\\
& \left.(X_j-\boldsymbol{X}_{-j}^\top\boldsymbol{w}_j^*)\right]\\
= & E_n^{(k)}\left[\left\{\widehat{\Omega}_+^{(k)}\phi'(\boldsymbol{X}^\top\boldsymbol{\beta}^*)-\widehat{\Omega}_-^{(k)}\phi'(-\boldsymbol{X}^\top\boldsymbol{\beta}^*)\right\}(X_j-\boldsymbol{X}_{-j}^\top\boldsymbol{w}_j^*)\right]\\
& +E_n^{(k)}\left[\left\{\widehat{\Omega}_+^{(k)}\phi''(\boldsymbol{X}^\top\boldsymbol{\beta}^*)+\widehat{\Omega}_-^{(k)}\phi''(-\boldsymbol{X}^\top\boldsymbol{\beta}^*)\right\}\boldsymbol{X}_{-j}^\top(\widehat{\boldsymbol{\beta}}_{-j}^{(k)}-\boldsymbol{\beta}_{-j}^*)(X_j-\boldsymbol{X}_{-j}^\top\boldsymbol{w}_j^*)\right]\\
& +E_n^{(k)}\left[\left\{\widehat{\Omega}_+^{(k)}\left[\phi''(\boldsymbol{X}^\top\boldsymbol{\beta}^*)-\phi''(\boldsymbol{X}^\top\boldsymbol{\beta}_{\text{null(j)}})\right]\right.\right.\\
& \left.\left.+\widehat{\Omega}_-^{(k)}\left[\phi''(-\boldsymbol{X}^\top\boldsymbol{\beta}^*)-\phi''(-\boldsymbol{X}^\top\boldsymbol{\beta}_{\text{null,j}})\right]\right\}\boldsymbol{X}_{-j}^\top(\widehat{\boldsymbol{\beta}}_{-j}^{(k)}-\boldsymbol{\beta}_{-j}^*)(X_j-\boldsymbol{X}_{-j}^\top\boldsymbol{w}_j^*)\right],
\end{aligned}
$$

$$
\begin{aligned}
& \left|E_n^{(k)}\left[\left\{\widehat{\Omega}_+^{(k)}\left[\phi''(\boldsymbol{X}^\top\boldsymbol{\beta}^*)-\phi''(\boldsymbol{X}^\top\beta_{\text{null}})\right]+\widehat{\Omega}_-^{(k)}\left[\phi''(-\boldsymbol{X}^\top\boldsymbol{\beta}^*)-\phi''(-\boldsymbol{X}^\top\boldsymbol{\beta}_{\text{null}})\right]\right\}\right.\right.\\
& \left.\left.\boldsymbol{X}_{-j}^\top(\widehat{\boldsymbol{\beta}}_{-j}^{(k)}-\boldsymbol{\beta}_{-j}^*)(X_j-\boldsymbol{X}_{-j}^\top\boldsymbol{w}_j^*)\right]\right|\\
\leq & E_n^{(k)}\left[\left\{\widehat{\Omega}_+^{(k)}\left|\phi''(\boldsymbol{X}^\top\boldsymbol{\beta}^*)-\phi''(\boldsymbol{X}^\top\beta_{\text{null}})\right|+\widehat{\Omega}_-^{(k)}\left|\phi''(-\boldsymbol{X}^\top\boldsymbol{\beta}^*)-\phi''(-\boldsymbol{X}^\top\beta_{\text{null}})\right|\right\}\right.\\
& \left.|\boldsymbol{X}_{-j}^\top(\widehat{\boldsymbol{\beta}}_{-j}^{(k)}-\boldsymbol{\beta}_{-j}^*)||X_j-\boldsymbol{X}_{-j}^\top\boldsymbol{w}_j^*|\right]\\
\leq & CE_n^{(k)}\left[\left\{\widehat{\Omega}_+^{(k)}\phi''(\boldsymbol{X}^\top\boldsymbol{\beta}^*)+\widehat{\Omega}_-^{(k)}\phi''(-\boldsymbol{X}^\top\boldsymbol{\beta}^*)\right\}|\boldsymbol{X}_{-j}^\top(\widehat{\boldsymbol{\beta}}_{-j}^{(k)}-\boldsymbol{\beta}_{-j}^*)|^2|X_j-\boldsymbol{X}_{-j}^\top\boldsymbol{w}_j^*|\right].
\end{aligned}
$$

By the sub-gaussian of $X_j-\boldsymbol{X}_{-j}^\top\boldsymbol{w}_j^*$ and $\sup_j\|\boldsymbol{w}_j^*\|_1\leq R$, there exists a constant σ_x such that

$$
P\left\{\max_{j=1,\cdots,p}\max_{1\leq i\leq n}|X_{i,j}-\boldsymbol{X}_{i,-j}^\top\boldsymbol{w}_j^*|\geq 2\sigma_xR\sqrt{\log(np)}\right\}\leq 2\exp\{-\log(np)\}.
$$

One the event that $\max_{j=1,\cdots,p}\max_{1\leq i\leq n}|X_{i,j}-X_{i,-j}^\top\boldsymbol{w}_j^*|\leq 2\sigma_wR\sqrt{\log(np)}$, we have

$$
\begin{aligned}
& E_n^{(k)}\left[\left\{\widehat{\Omega}_+^{(k)}\phi''(\boldsymbol{X}^\top\boldsymbol{\beta}^*)+\widehat{\Omega}_-^{(k)}\phi''(-\boldsymbol{X}^\top\boldsymbol{\beta}^*)\right\}|\boldsymbol{X}_{-j}^\top(\widehat{\beta}_{-1}^{(k)}-\beta_{-1}^*)|^2|X_j-\boldsymbol{X}_{-j}^\top\boldsymbol{w}_j^*|\right]\\
\leq & 2\sigma_wR\sqrt{\log(np)}E_n^{(k)}\left[\left\{\widehat{\Omega}_+^{(k)}\phi''(\boldsymbol{X}^\top\boldsymbol{\beta}^*)+\widehat{\Omega}_-^{(k)}\phi''(-\boldsymbol{X}^\top\boldsymbol{\beta}^*)\right\}|\boldsymbol{X}_{-j}^\top(\widehat{\beta}_{-1}^{(k)}-\beta_{-1}^*)|^2\right].
\end{aligned}
$$

By Lemma 10, we have that

$$
\begin{aligned}
& \sup_{j=1,\cdots,p}E_n^{(k)}\left[\left\{\widehat{\Omega}_+^{(k)}\phi''(\boldsymbol{X}^\top\boldsymbol{\beta}^*)+\widehat{\Omega}_-^{(k)}\phi''(-\boldsymbol{X}^\top\boldsymbol{\beta}^*)\right\}|\boldsymbol{X}_{-j}^\top(\widehat{\boldsymbol{\beta}}_{-j}^{(k)}-\boldsymbol{\beta}_{-j}^*)|^2\right]\\
= & O_p(s^*\log p/n).
\end{aligned}
$$

Thus, we have that

$$
\begin{aligned}
& E_n^{(k)}\left[\left\{\widehat{\Omega}_+^{(k)}\phi''(\boldsymbol{X}^\top\boldsymbol{\beta}^*)+\widehat{\Omega}_-^{(k)}\phi''(-\boldsymbol{X}^\top\boldsymbol{\beta}^*)\right\}|\boldsymbol{X}_{-j}^\top(\widehat{\beta}_{-1}^{(k)}-\beta_{-1}^*)|^2|X_j-\boldsymbol{X}_{-j}^\top\boldsymbol{w}_j^*|\right]\\
= & O_p(R\sqrt{\log(np)}s^*\log p/n)
\end{aligned}
$$

Under the condition $Rs^*(\log p)^{3/2}/\sqrt{n} \to 0$, we have Line (12) to (13).

Now, we focus on a bound for (13). For the first term in (13), by Claim 13, we have that

$$\begin{aligned}
& n^{1/2} E_n^{(k)} \left[\left\{ \widehat{\Omega}_+^{(k)} \phi'(\boldsymbol{X}^\top \boldsymbol{\beta}^*) - \widehat{\Omega}_-^{(k)} \phi'(-\boldsymbol{X}^\top \boldsymbol{\beta}^*) \right\} (X_j - \boldsymbol{X}_{-j}^\top \boldsymbol{w}_j^*) \right] \\
= \; & n^{1/2} E_n^{(k)} \left[\left\{ \Omega_+ \phi'(\boldsymbol{X}^\top \boldsymbol{\beta}^*) - \Omega_- \phi'(-\boldsymbol{X}^\top \boldsymbol{\beta}^*) \right\} (X_j - \boldsymbol{X}_{-j}^\top \boldsymbol{w}_j^*) \right] \\
& + n^{1/2} E_n^{(k)} \left[\left\{ \left[\widehat{\Omega}_+^{(k)} - \Omega_+ \right] \phi'(\boldsymbol{X}^\top \boldsymbol{\beta}^*) - \left[\widehat{\Omega}_-^{(k)} - \Omega_- \right] \phi'(-\boldsymbol{X}^\top \boldsymbol{\beta}^*) \right\} (X_j - \boldsymbol{X}_{-j}^\top \boldsymbol{w}_j^*) \right] \\
= \; & n^{1/2} E_n^{(k)} \left[\left\{ \Omega_+ \phi'(\boldsymbol{X}^\top \boldsymbol{\beta}^*) - \Omega_- \phi'(-\boldsymbol{X}^\top \boldsymbol{\beta}^*) \right\} (X_j - \boldsymbol{X}_{-j}^\top \boldsymbol{w}_j^*) \right] + \\
& O_p\left(R n^{-\alpha-\beta} + R(n^{-\alpha} + n^{-\beta}) \sqrt{\log p / n} \right),
\end{aligned}$$

uniformaly holds in j. By the conditions, we have

$$R n^{-\alpha-\beta+1/2} \to 0, \quad R(n^{-\alpha} + n^{-\beta}) \sqrt{\log p} \to 0.$$

Thus, we have that the first term in (13) is equivalent to

$$(n/K)^{1/2} E_n^{(k)} \left[\left\{ \Omega_+ \phi'(\boldsymbol{X}^\top \boldsymbol{\beta}^*) - \Omega_- \phi'(-\boldsymbol{X}^\top \boldsymbol{\beta}^*) \right\} (X_j - \boldsymbol{X}_{-j}^\top \boldsymbol{w}_j^*) \right]$$

uniformly.

The second term in (13) can be bounded by the following

$$\begin{aligned}
& \max_{j \in \mathcal{J}} \Big| (n/K)^{1/2} E_n^{(k)} \left[\left\{ \widehat{\Omega}_+^{(k)} \phi''(\boldsymbol{X}^\top \boldsymbol{\beta}^*) + \widehat{\Omega}_-^{(k)} \phi''(-\boldsymbol{X}^\top \boldsymbol{\beta}^*) \right\} \right. \\
& \left. \boldsymbol{X}_{-j}^\top (\widehat{\boldsymbol{\beta}}_{-j}^{(k)} - \boldsymbol{\beta}_{-j}^*)(X_j - \boldsymbol{X}_{-j}^\top \boldsymbol{w}_j^*) \right] \Big| \\
\le \; & (n/K)^{1/2} \max_{j \in \mathcal{J}} \left\| E_n^{(k)} \left[\left\{ \widehat{\Omega}_+^{(k)} \phi''(\boldsymbol{X}^\top \boldsymbol{\beta}^*) + \widehat{\Omega}_-^{(k)} \phi''(-\boldsymbol{X}^\top \boldsymbol{\beta}^*) \right\} (X_j - \boldsymbol{X}_{-j}^\top \boldsymbol{w}_j^*) \boldsymbol{X}_{-j} \right] \right\|_\infty \\
& \left\| \widehat{\boldsymbol{\beta}}^{(k)} - \boldsymbol{\beta}^* \right\|_1 \\
\le \; & (n/K)^{1/2} \max_{j \in \mathcal{J}} \left\| E_n^{(k)} \left[\left\{ \Omega_+ \phi''(\boldsymbol{X}^\top \boldsymbol{\beta}^*) + \Omega_- \phi''(-\boldsymbol{X}^\top \boldsymbol{\beta}^*) \right\} (X_j - \boldsymbol{X}_{-j}^\top \boldsymbol{w}_j^*) \boldsymbol{X}_{-j} \right] \right\|_\infty \\
& \left\| \widehat{\boldsymbol{\beta}}^{(k)} - \boldsymbol{\beta}^* \right\|_1 + O_p\left(\left(R n^{-\alpha-\beta+1/2} + R(n^{-\alpha} + n^{-\beta}) \sqrt{\log p} \right) s^* (\log p / n)^{1/2} \right).
\end{aligned}$$

The second equality comes from Claim 13 and Lemma 10. To bound

$$\left\| E_n^{(k)} \left[\left\{ \Omega_+ \phi''(\boldsymbol{X}^\top \boldsymbol{\beta}^*) + \Omega_- \phi''(-\boldsymbol{X}^\top \boldsymbol{\beta}^*) \right\} (X_j - \boldsymbol{X}_{-j}^\top \boldsymbol{w}_j^*) \boldsymbol{X}_{-j} \right] \right\|_\infty,$$

we consider the following decomposition

$$\begin{aligned}
& \left\| E_n^{(k)} \left[\left\{ \Omega_+ \phi''(\boldsymbol{X}^\top \boldsymbol{\beta}^*) + \Omega_- \phi''(-\boldsymbol{X}^\top \boldsymbol{\beta}^*) \right\} (X_j - \boldsymbol{X}_{-j}^\top \boldsymbol{w}_j^*) \boldsymbol{X}_{-j} \right] \right\|_\infty \\
\le \; & \Bigg\| E_n^{(k)} \Bigg[\Bigg\{ \frac{1\{A = 1\}}{\pi_1} (Y - Q(1; \boldsymbol{X})) \phi''(\boldsymbol{X}^\top \boldsymbol{\beta}^*) + \\
& \frac{1\{A = -1\}}{\pi_{-1}} (Y - Q(-1; \boldsymbol{X})) \phi''(-\boldsymbol{X}^\top \boldsymbol{\beta}^*) \Bigg\} (X_j - \boldsymbol{X}_{-j}^\top \boldsymbol{w}_j^*) \boldsymbol{X}_{-j} \Bigg] \Bigg\|_\infty \\
& + \left\| E_n^{(k)} \left[\left\{ Q(1; \boldsymbol{X}) \phi''(\boldsymbol{X}^\top \boldsymbol{\beta}^*) + Q(1; \boldsymbol{X}) \phi''(-\boldsymbol{X}^\top \boldsymbol{\beta}^*) \right\} (X_j - \boldsymbol{X}_{-j}^\top \boldsymbol{w}_j^*) \boldsymbol{X}_{-j} \right] \right\|_\infty.
\end{aligned}$$

By the condition on $Y - Q(a;\boldsymbol{X})$ on $A = a$, by the proof of Claim 13, the first term can be bounded

$$\begin{aligned} & n^{1/2}\max_{j\in\mathcal{J}}\left\|E_n^{(k)}\left[\left\{\frac{1\{A=1\}}{\pi_1}\left(Y-Q(1;\boldsymbol{X})\right)\phi''(\boldsymbol{X}^\top\boldsymbol{\beta}^*)+\right.\right.\right. \\ & \left.\left.\left.\frac{1\{A=-1\}}{\pi_{-1}}\left(Y-Q(-1;\boldsymbol{X})\right)\phi''(-\boldsymbol{X}^\top\boldsymbol{\beta}^*)\right\}(X_j-\boldsymbol{X}_{-j}^\top\boldsymbol{w}_j^*)\boldsymbol{X}_{-j}\right]\right\|_\infty \\ = & \ O_p(R\sqrt{\log p/n}). \end{aligned}$$

For the second term, by the boundedness of $Q(a;\boldsymbol{X})$'s and $\phi^{''}(\cdot)$, by Lemma 14 in Loh and Wainwright (2015), if $\log p = O(n)$, we have

$$\begin{aligned} & \max_{j\in\mathcal{J}}\left\|E_n^{(k)}\left[\left\{Q(1;\boldsymbol{X})\phi''(\boldsymbol{X}^\top\boldsymbol{\beta}^*)+Q(1;\boldsymbol{X})\phi''(-\boldsymbol{X}^\top\boldsymbol{\beta}^*)\right\}(X_j-\boldsymbol{X}_{-j}^\top\boldsymbol{w}_j^*)\boldsymbol{X}_{-j}\right]\right\|_\infty \\ = & \ O_p(R\sqrt{\log p/n}). \end{aligned}$$

Because our condition that

$$Rn^{-1/2}s^*\log p\to 0,\quad Rn^{-\alpha-\beta+1/2}\to 0,\quad \text{and } R(n^{-\alpha}+n^{-\beta})\sqrt{\log p}\to 0,$$

the second term in (13) is $o_p(1)$.

The third term in (13) can also be bounded following a similar steps to the first term in (13).

$$\begin{aligned} & \left|(n/K)^{1/2}E_n^{(k)}\left[\left\{\widehat{\Omega}_+^{(k)}\phi'(\boldsymbol{X}^\top\widehat{\boldsymbol{\beta}}_{\text{null(j)}}^{(k)})-\widehat{\Omega}_-^{(k)}\phi'(-\boldsymbol{X}^\top\widehat{\boldsymbol{\beta}}_{\text{null(j)}}^{(k)})\right\}(\boldsymbol{X}_{-j}^\top\widehat{\boldsymbol{w}}_j^{(k)}-\boldsymbol{X}_{-j}^\top\boldsymbol{w}_j^*)\right]\right| \\ \le & \left|(n/K)^{1/2}E_n^{(k)}\left[\left\{\widehat{\Omega}_+^{(k)}\phi'(\boldsymbol{X}^\top\boldsymbol{\beta}^*)-\widehat{\Omega}_-^{(k)}\phi'(-\boldsymbol{X}^\top\boldsymbol{\beta}^*)\right\}(\boldsymbol{X}_{-j}^\top\widehat{\boldsymbol{w}}^{(k)}-\boldsymbol{X}_{-j}^\top\boldsymbol{w}_j^*)\right]\right| \\ & +\left|(n/K)^{1/2}E_n^{(k)}\left[\left\{\widehat{\Omega}_+^{(k)}\phi^{''}(\boldsymbol{X}^\top\boldsymbol{\beta}^*)+\widehat{\Omega}_-^{(k)}\phi^{''}(-\boldsymbol{X}^\top\boldsymbol{\beta}^*)\right\}\right.\right. \\ & \left.\left.\boldsymbol{X}_{-j}^\top(\widehat{\boldsymbol{w}}_j^{(k)}-\boldsymbol{w}_j^*)\boldsymbol{X}_{-j}^\top(\widehat{\boldsymbol{\beta}}_{-j}^{(k)}-\boldsymbol{\beta}_{-j}^*)\right]\right|+o_p(1), \end{aligned}$$

uniformly in j. To bound the second term above, by Lemma 10 and 11, we have that

$$\begin{aligned} & \max_{j\in\mathcal{J}}\left|(n/K)^{1/2}E_n^{(k)}\left[\left\{\widehat{\Omega}_+^{(k)}\phi^{''}(\boldsymbol{X}^\top\boldsymbol{\beta}^*)+\widehat{\Omega}_-^{(k)}\phi^{''}(-\boldsymbol{X}^\top\boldsymbol{\beta}^*)\right\}\right.\right. \\ & \left.\left.\boldsymbol{X}_{-j}^\top(\widehat{\boldsymbol{w}}_j^{(k)}-\boldsymbol{w}_j^*)\boldsymbol{X}_{-j}^\top(\widehat{\boldsymbol{\beta}}_{-j}^{(k)}-\boldsymbol{\beta}_{-j}^*)\right]\right| \\ \le & \ (n/K)^{1/2}\left\{\max_{j\in\mathcal{J}}E_n^{(k)}\left[\left\{\widehat{\Omega}_+^{(k)}\phi^{''}(\boldsymbol{X}^\top\boldsymbol{\beta}^*)+\widehat{\Omega}_-^{(k)}\phi^{''}(-\boldsymbol{X}^\top\boldsymbol{\beta}^*)\right\}\left|\boldsymbol{X}_{-j}^\top(\widehat{\boldsymbol{w}}_j^{(k)}-\boldsymbol{w}_j^*)\right|^2\right]\right. \\ & \left.\max_{j\in\mathcal{J}}E_n^{(k)}\left[\left\{\widehat{\Omega}_+^{(k)}\phi^{''}(\boldsymbol{X}^\top\boldsymbol{\beta}^*)+\widehat{\Omega}_-^{(k)}\phi^{''}(-\boldsymbol{X}^\top\boldsymbol{\beta}^*)\right\}\left|\boldsymbol{X}_{-j}^\top(\widehat{\boldsymbol{\beta}}_{-j}^{(k)}-\boldsymbol{\beta}_{-j}^*)\right|^2\right]\right\}^{1/2} \\ = & \ o_p(1). \end{aligned}$$

To bound the first term, we have

$$\begin{aligned}
& \max_{j\in\mathcal{J}} \left|(n/K)^{1/2}E_n^{(k)}\left[\left\{\widehat{\Omega}_+^{(k)}\phi'(\boldsymbol{X}^\top\boldsymbol{\beta}^*)-\widehat{\Omega}_-^{(k)}\phi'(-\boldsymbol{X}^\top\boldsymbol{\beta}^*)\right\}(\boldsymbol{X}_{-j}^\top\widehat{\boldsymbol{w}}_j^{(k)}-\boldsymbol{X}_{-j}^\top\boldsymbol{w}_j^*)\right]\right| \\
\leq\ & \max_{j\in\mathcal{J}} \left\|(n/K)^{1/2}E_n^{(k)}\left[\left\{\widehat{\Omega}_+^{(k)}\phi'(\boldsymbol{X}^\top\boldsymbol{\beta}^*)-\widehat{\Omega}_-^{(k)}\phi'(-\boldsymbol{X}^\top\boldsymbol{\beta}^*)\right\}\boldsymbol{X}_{-j}\right]\right\|_\infty\left\|\widehat{\boldsymbol{w}}_j^{(k)}-\boldsymbol{w}_j^*)\right\|_1 \\
\leq\ & \left\|(n/K)^{1/2}E_n^{(k)}\left[\left\{\Omega_+\phi'(\boldsymbol{X}^\top\boldsymbol{\beta}^*)-\Omega_-\phi'(-\boldsymbol{X}^\top\boldsymbol{\beta}^*)\right\}\boldsymbol{X}\right]\right\|_\infty \max_{j\in\mathcal{J}}\left\|\widehat{\boldsymbol{w}}_j^{(k)}-\boldsymbol{w}_j^*)\right\|_1 \\
& +\left\|(n/K)^{1/2}E_n^{(k)}\left[\left\{\left(\widehat{\Omega}_+^{(k)}-\Omega_+\right)\phi'(\boldsymbol{X}^\top\boldsymbol{\beta}^*)-\left(\widehat{\Omega}_-^{(k)}-\Omega_-\right)\phi'(-\boldsymbol{X}^\top\boldsymbol{\beta}^*)\right\}\boldsymbol{X}\right]\right\|_\infty \\
& \max_{j\in\mathcal{J}}\left\|\widehat{\boldsymbol{w}}_j^{(k)}-\boldsymbol{w}_j^*)\right\|_1
\end{aligned}$$

For the second term, by Lemma 11 and Claim 13, we know that the second term is negligible. For the first term, we decompose it into two terms, i.e.,

$$\begin{aligned}
& n^{1/2}E_n^{(k)}\left[\left\{\Omega_+\phi'(\boldsymbol{X}^\top\boldsymbol{\beta}^*)-\Omega_-\phi'(-\boldsymbol{X}^\top\boldsymbol{\beta}^*)\right\}\boldsymbol{X}\right] \\
=\ & n^{1/2}E_n^{(k)}\left[\left\{\frac{1\{A=1\}}{\pi_1}\left(Y-Q(1;\boldsymbol{X})\right)\phi'(\boldsymbol{X}^\top\boldsymbol{\beta}^*)\right.\right. \\
& \left.\left.-\frac{1\{A=-1\}}{\pi_{-1}}\left(Y-Q(-1;\boldsymbol{X})\right)\phi'(-\boldsymbol{X}^\top\boldsymbol{\beta}^*)\right\}\boldsymbol{X}\right] \\
& +n^{1/2}E_n^{(k)}\left[\left\{Q(1;\boldsymbol{X})\phi'(\boldsymbol{X}^\top\boldsymbol{\beta}^*)-Q(-1;\boldsymbol{X})\phi'(-\boldsymbol{X}^\top\boldsymbol{\beta}^*)\right\}\boldsymbol{X}\right].
\end{aligned}$$

Because $Q(a;\boldsymbol{X})$'s are bounded and $\phi^{'}(\cdot)$ is bounded, by the sub-Gaussian condition on $\boldsymbol{X}$, under the null hypothesis, we have that

$$\begin{aligned}
& \max_{j\in\mathcal{J}}\left\|E_n^{(k)}\left[\left\{Q(1;\boldsymbol{X})\phi'(\boldsymbol{X}^\top\boldsymbol{\beta}^*)-Q(-1;\boldsymbol{X})\phi'(-\boldsymbol{X}^\top\boldsymbol{\beta}^*)\right\}\boldsymbol{X}\right]\right\|_\infty \\
=\ & O_p(\sqrt{\log p/n}).
\end{aligned}$$

By the condition of $Y-Q(a;\boldsymbol{X})$ on $A=1$ and the sub-Gaussian condition on $\boldsymbol{X}$, by the proof of Claim 13, if $\log p = O(n)$, we have

$$\begin{aligned}
& \max_{j\in\mathcal{J}}\left|E_n^{(k)}\left[\frac{1\{A=1\}}{\pi_1}\left(Y-Q(1;\boldsymbol{X})\right)\phi'(\boldsymbol{X}^\top\boldsymbol{\beta}^*)(X_j-\boldsymbol{X}_{-j}^\top\boldsymbol{w}_j^*)\right]\right| \\
=\ & O_p(\sqrt{\log p/n}).
\end{aligned}$$

Similarly, we can show that

$$\begin{aligned}
& \max_{j\in\mathcal{J}}\left\|E_n^{(k)}\left[\frac{1\{A=-1\}}{\pi_{-1}}\left(Y-Q(-1;\boldsymbol{X})\right)\phi'(-\boldsymbol{X}^\top\boldsymbol{\beta}^*)\boldsymbol{X}\right]\right\|_\infty \\
=\ & O_p(\sqrt{\log p/n}).
\end{aligned}$$

Thus, the first term is $O_p(n^{1/2}\sqrt{\log p/n}\max\{s^*,s'\}\sqrt{\log p/n})$, which is negligiable.

In conclusion, we have that

$$\begin{aligned}
& \max_{j\in\mathcal{J}}\left|(n/K)^{1/2}S_j^{(k)}\left(\widehat{\boldsymbol{\beta}}_{\text{null(j)}}^{(k)},\widehat{\boldsymbol{w}}_j^{(k)}\right)-(n/K)^{1/2}E_n^{(k)}\left[\left\{\Omega_+\phi'(\boldsymbol{X}^\top\boldsymbol{\beta}^*)\right.\right.\right. \\
& \left.\left.\left.-\Omega_-\phi'(-\boldsymbol{X}^\top\boldsymbol{\beta}^*)\right\}(X_j-\boldsymbol{X}_{-j}^\top\boldsymbol{w}_j^*)\right]\right| = o_p(1).
\end{aligned}$$

Define

$$\sigma_j^2 = E\left[\left\{\Omega_+\phi'(\boldsymbol{X}^\top\boldsymbol{\beta}) - \Omega_-\phi'(-\boldsymbol{X}^\top\boldsymbol{\beta})\right\}^2 (X_j - \boldsymbol{X}_{-j}^\top \boldsymbol{w}_j^*)^2\right].$$

Applying the Berry-Esseen bound for CLT, there exists some universal constant such that

$$\begin{aligned}
&\max_{j\in\mathcal{J}} \sup_{\alpha\in(0,1)} \Big| P\Big(\Big|\sigma_j^{-1}(n/K)^{1/2} E_n^{(k)} \Big[\Big\{\Omega_+\phi'(\boldsymbol{X}^\top\boldsymbol{\beta}^*) \\
&\quad -\Omega_-\phi'(-\boldsymbol{X}^\top\boldsymbol{\beta}^*)\Big\}(X_j - \boldsymbol{X}_{-j}^\top\boldsymbol{w}_j^*)\Big]\Big| \le \Phi^{-1}(1-\alpha/2)\Big) - (1-\alpha)\Big| \\
\le\ & \frac{c_0}{\sqrt{n}} \max_j E[|M_j|^3],
\end{aligned}$$

where

$$M_j = \left\{\Omega_+\phi'(\boldsymbol{X}^\top\boldsymbol{\beta}^*) - \Omega_-\phi'(-\boldsymbol{X}^\top\boldsymbol{\beta}^*)\right\}(X_j - \boldsymbol{X}_{-j}^\top\boldsymbol{w}_j^*).$$

From the sub-Gaussian conditions, we have that $\max_j E[|M_j|^3] = O(R^3)$. Thus, if $R^3/\sqrt{n} \to 0$, we have that $\frac{c_0}{\sqrt{n}} \max_j E[|M_j|^3] \to 0$.

Combining the inequalities above, we have

$$\max_{j\in\mathcal{J}} \sup_{\alpha\in(0,1)} \left| P\left(\left|\sigma_j^{-1}(n/K)^{1/2} S_j^{(k)}\left(\widehat{\boldsymbol{\beta}}^{(k)}_{\mathrm{null(j)}}, \widehat{\boldsymbol{w}}_j^{(k)}\right)\right| \le \Phi^{-1}(1-\alpha/2)\right) - (1-\alpha)\right| = o_p(1).$$

Averaging all the k's, we can conclude the proof.

□

Proof of Theorem 8. We can observe that if Claim 13 holds with

$$n^{1/2}\left\|E_n\left[\left(\widehat{\Omega}_a^{(k)} - \Omega_a^{(k)}\right)h(\boldsymbol{X})\boldsymbol{X}\right]\right\|_\infty = o_p(1),$$

then the proof of Theorem 4 is applicable to Algorithm 2. However, the proof of Claim 13 uses the fact that the data used to train $\widehat{\pi}^{(-k)}$ and $\widehat{Q}^{(-k)}$ is independent with those used to fit the proposed method and form the score test statistic. Here, we provide a separate proof of Claim 13 utilizing the parametric structure. Without loss of generality, we assume that π is estimated parametrically. To start with, denote the parametric model of π as $\pi(A;\boldsymbol{X},\boldsymbol{\beta})$. Under this notation, we rewrite that $\widehat{\pi}(A;\boldsymbol{X}) = \pi\left(A;\boldsymbol{X},\widehat{\boldsymbol{\beta}}_\pi\right)$ and $\pi(A;\boldsymbol{X}) = \pi\left(A;\boldsymbol{X},\boldsymbol{\beta}_\pi^*\right)$.

We want to show that for any function h with $\|h\|_\infty \le C$, we have for $a = 1$ or -1,

$$\left\|E_n\left[\left(\widehat{\Omega}_a^{(k)} - \Omega_a^{(k)}\right)h(\boldsymbol{X})\boldsymbol{X}\right]\right\|_\infty = O_p\left(n^{-\alpha-\beta} + (n^{-\alpha}+n^{-\beta})(\log p/n)^{1/2}\right).$$

We consider $a = 1$. Note that

$$\begin{aligned}
&\left\|E_n\left[\left(\widehat{\Omega}_a^{(k)} - \Omega_a\right)h(\boldsymbol{X})\boldsymbol{X}\right]\right\|_\infty \\
\le & \left\|E_n\left[I\{A=1\}\left(\widehat{\pi}^{-1}(1;\boldsymbol{X}) - \pi^{-1}(1;\boldsymbol{X})\right)\left(\widehat{Q}^{(-k)}(1;\boldsymbol{X}) - Q(1;\boldsymbol{X})\right)h(\boldsymbol{X})\boldsymbol{X}\right]\right\|_\infty \\
&+ \left\|E_n\left[I\{A=1\}\left(\widehat{\pi}^{-1}(1;\boldsymbol{X}) - \pi^{-1}(1;\boldsymbol{X})\right)(Y_1 - Q(1;\boldsymbol{X}))h(\boldsymbol{X})\boldsymbol{X}\right]\right\|_\infty \\
&+ \left\|E_n\left[(I\{A=1\}/\pi(1;\boldsymbol{X}) - 1)\left(\widehat{Q}^{(-k)}(1;\boldsymbol{X}) - Q(1;\boldsymbol{X})\right)h(\boldsymbol{X})\boldsymbol{X}\right]\right\|_\infty \\
=& I_1 + I_2 + I_3
\end{aligned}$$

For I_1, we can show that $I_1 \lesssim n^{-\beta}\left\|\widehat{\boldsymbol{\beta}}_\pi - \boldsymbol{\beta}_\pi^*\right\|_1$ following the proof of Claim 13 and $\left(\widehat{\boldsymbol{\beta}}_\pi - \boldsymbol{\beta}_\pi^*\right)^\top E_n[\boldsymbol{X}\boldsymbol{X}^\top]\left(\widehat{\boldsymbol{\beta}}_\pi - \boldsymbol{\beta}_\pi^*\right) \lesssim n^{-1/2}$. Likewise, from the proof of Claim 13, we can conclude that $I_3 \lesssim O_p\left(n^{-\beta}(\log p/n)^{1/2}\right)$. For I_2, we have

$$\begin{aligned}
& \left\|E_n\left[I\{A=1\}\left(\widehat{\pi}^{-1}(1;\boldsymbol{X}) - \pi^{1/2}(1;\boldsymbol{X})\right)(Y_1 - Q(1;\boldsymbol{X}))\,h(\boldsymbol{X})\boldsymbol{X}\right]\right\|_\infty \\
= & \left\|E_n\left[I\{A=1\}\left(\pi^{-1}\left(A;\boldsymbol{X},\widehat{\boldsymbol{\beta}}_\pi\right) - \pi^{-1}\left(A;\boldsymbol{X},\boldsymbol{\beta}_\pi^*\right)\right)(Y_1 - Q(1;\boldsymbol{X}))\,h(\boldsymbol{X})\boldsymbol{X}\right]\right\|_\infty \\
\lesssim & \left\|E_n\left[-I\{A=1\}\pi^{-2}\left(A;\boldsymbol{X},\boldsymbol{\beta}_\pi^*\right)\nabla_{\boldsymbol{\beta}}\pi(A;\boldsymbol{X},\boldsymbol{\beta}^*)\left(\widehat{\boldsymbol{\beta}}_\pi - \boldsymbol{\beta}^*\right)^\top(Y_1 - Q(1;\boldsymbol{X}))\right.\right. \\
& \left.\left. h(\boldsymbol{X})\boldsymbol{X}\right]\right\|_\infty \\
\leq & \left\|E_n\left[-\boldsymbol{X}I\{A=1\}\pi^{-2}\left(A;\boldsymbol{X},\boldsymbol{\beta}_\pi^*\right)\{\nabla_{\boldsymbol{\beta}}\pi(A;\boldsymbol{X},\boldsymbol{\beta}^*)\}^\top(Y_1 - Q(1;\boldsymbol{X}))\,h(\boldsymbol{X})\right]\right\|_\infty \\
& \times\left\|\widehat{\boldsymbol{\beta}}_\pi - \boldsymbol{\beta}^*\right\|_1 \\
= & \left\|\widehat{\boldsymbol{\beta}}_\pi - \boldsymbol{\beta}^*\right\|_1(\log p/n)^{1/2}
\end{aligned}$$

When π is estimated by linear or logistic regression with lasso penalty, we have

$$\left\|\widehat{\boldsymbol{\beta}}_\pi - \boldsymbol{\beta}^*\right\|_1 \lesssim n^{-\alpha}.$$

Thus, the claim holds. □

Proof of Theorem 5. First, we will show that

$$(n/K)^{1/2}\max_j\left|\left(\widetilde{\beta}_j^{(k)} - \beta_j^*\right)\widehat{I}_{j|-j}^{(k)} + E_n^{(k)}\left[\{\nabla l_\phi\left(\boldsymbol{\beta}^*;\Omega_+,\Omega_-\right)\}\left(X_j - \boldsymbol{X}_{-j}^\top\boldsymbol{w}_j^*\right)\right]\right| = o_p(1).$$

By the definition of $\widetilde{\beta}_j^{(k)}$,

$$\begin{aligned}
& (n/K)^{1/2}\left|\left(\widetilde{\beta}_j^{(k)} - \beta_j^*\right)\widehat{I}_{j|-j}^{(k)} + E_n^{(k)}\left[\{\nabla l_\phi\left(\boldsymbol{\beta}^*;\Omega_+,\Omega_-\right)\}\left(X_j - \boldsymbol{X}_{-j}^\top\boldsymbol{w}_j^*\right)\right]\right| \\
= & (n/K)^{1/2}\left|\left(\widetilde{\beta}_j^{(k)} - \beta_j^*\right)\widehat{I}_{j|-j}^{(k)} - \left\{S_j^{(k)}\left(\widehat{\boldsymbol{\beta}}^{(k)},\widehat{\boldsymbol{w}}_j^{(k)}\right) - E_n^{(k)}\left[\{\nabla l_\phi\left(\boldsymbol{\beta}^*;\Omega_+,\Omega_-\right)\}\right.\right.\right. \\
& \left.\left.\left.\left(X_j - \boldsymbol{X}_{-j}^\top\boldsymbol{w}_j^*\right)\right]\right\}\right| \\
\leq & (n/K)^{1/2}\left|\left(\widetilde{\beta}_j^{(k)} - \beta_j^*\right)\widehat{I}_{j|-j}^{(k)} - \left\{S_j^{(k)}\left(\widehat{\boldsymbol{\beta}}^{(k)},\widehat{\boldsymbol{w}}_j^{(k)}\right) - S_j^{(k)}\left(\widehat{\boldsymbol{\beta}}_{\text{null(j)}}^{(k)},\widehat{\boldsymbol{w}}_j^{(k)}\right)\right\}\right| \\
& (n/K)^{1/2}\left|S_j^{(k)}\left(\widehat{\boldsymbol{\beta}}_{\text{null(j)}}^{(k)},\widehat{\boldsymbol{w}}_j^{(k)}\right) - E_n^{(k)}\left[\{\nabla l_\phi\left(\boldsymbol{\beta}^*;\Omega_+,\Omega_-\right)\}\left(X_j - \boldsymbol{X}_{-j}^\top\boldsymbol{w}_j^*\right)\right]\right| \\
\leq & I_{1j} + I_{2j},
\end{aligned}$$

where $\widehat{\boldsymbol{\beta}}_{\text{null(j)}}^{(k)}$ equals to $\widehat{\boldsymbol{\beta}}^{(k)}$ except its jth coefficient replaced by β_j^*.

By the proof of Theorem 4, we have

$$\max_j I_{2j} = o_p(1).$$

To bound I_{1j} uniformly, we consider

$$
\begin{aligned}
& (n/K)^{1/2} S_j^{(k)}\left(\widehat{\boldsymbol{\beta}}^{(k)}, \widehat{\boldsymbol{w}}_j^{(k)}\right) - S_j^{(k)}\left(\widehat{\boldsymbol{\beta}}_{\text{null(j)}}^{(k)}, \widehat{\boldsymbol{w}}_j^{(k)}\right) \\
= \; & (n/K)^{1/2} E_n^{(k)}\left[\left\{\widehat{\Omega}_+^{(k)} \phi''(\boldsymbol{X}^\top \boldsymbol{\beta}^*) + \widehat{\Omega}_-^{(k)} \phi''(-\boldsymbol{X}^\top \boldsymbol{\beta}^*)\right\} X_j(\widehat{\beta}_j^{(k)} - \beta_j^*)(X_j - \boldsymbol{X}_{-j}^\top \boldsymbol{w}_j^*)\right] \\
& + (n/K)^{1/2} E_n^{(k)}\left[\left\{\widehat{\Omega}_+^{(k)} \phi''(\boldsymbol{X}^\top \boldsymbol{\beta}_{\text{null(j)}}) + \widehat{\Omega}_-^{(k)} \phi''(-\boldsymbol{X}^\top \boldsymbol{\beta}_{\text{null(j)}})\right\} X_j(\widehat{\beta}_j^{(k)} - \beta_j^*)\right. \\
& \left. \boldsymbol{X}_{-j}^\top(\widehat{\boldsymbol{w}}_j^{(k)} - \boldsymbol{w}_j^*)\right] \\
& + (n/K)^{1/2} E_n^{(k)}\left[\left\{\widehat{\Omega}_+^{(k)}\left\{\phi''(\boldsymbol{X}^\top \boldsymbol{\beta}_{\text{null(j)}}) - \phi''(\boldsymbol{X}^\top \boldsymbol{\beta}^*)\right\} + \right.\right. \\
& \left.\left. \widehat{\Omega}_-^{(k)}\left\{\phi''(-\boldsymbol{X}^\top \boldsymbol{\beta}_{\text{null(j)}}) - \phi''(-\boldsymbol{X}^\top \boldsymbol{\beta}^*)\right\}\right\} X_j(\widehat{\beta}_j^{(k)} - \beta_j^*)(X_j - \boldsymbol{X}_{-j}^\top \boldsymbol{w}_j^*\right],
\end{aligned}
$$

where $\boldsymbol{\beta}_{\text{null(j)}}$ is in between $\widehat{\boldsymbol{\beta}}_{\text{null(j)}}^{(k)}$ and $\widehat{\boldsymbol{\beta}}_j^{(k)}$. By Claim 13 and the proof of Theorem 4, we can see that

$$
\begin{aligned}
& (n/K)^{1/2} E_n^{(k)}\left[\left\{\widehat{\Omega}_+^{(k)} \phi''(\boldsymbol{X}^\top \boldsymbol{\beta}^*) + \widehat{\Omega}_-^{(k)} \phi''(-\boldsymbol{X}^\top \boldsymbol{\beta}^*)\right\} X_j(\widehat{\beta}_j^{(k)} - \beta_j^*)(X_j - \boldsymbol{X}_{-j}^\top \boldsymbol{w}_j^*)\right] \\
= \; & (n/K)^{1/2}\left(\widehat{\beta}_j^{(k)} - \beta_j^*\right) I_{j|-j} + o_p(1),
\end{aligned}
$$

uniformly holds over $j = 1, \cdots, J$. For the third term, we have

$$
\begin{aligned}
& (n/K)^{1/2}\left|E_n^{(k)}\left[\left\{\widehat{\Omega}_+^{(k)}\left\{\phi''(\boldsymbol{X}^\top \boldsymbol{\beta}_{\text{null(j)}}) - \phi''(\boldsymbol{X}^\top \boldsymbol{\beta}^*)\right\} + \right.\right.\right. \\
& \left.\left.\left. \widehat{\Omega}_-^{(k)}\left\{\phi''(-\boldsymbol{X}^\top \boldsymbol{\beta}_{\text{null(j)}}) - \phi''(-\boldsymbol{X}^\top \boldsymbol{\beta}^*)\right\}\right\} X_j(\widehat{\beta}_j^{(k)} - \beta_j^*)(X_j - \boldsymbol{X}_{-j}^\top \boldsymbol{w}_j^*)\right]\right| \\
\leq \; & (n/K)^{1/2} C E_n^{(k)}\left[\left\{\widehat{\Omega}_+^{(k)} \phi''(\boldsymbol{X}^\top \boldsymbol{\beta}^*) + \widehat{\Omega}_-^{(k)} \phi''(-\boldsymbol{X}^\top \boldsymbol{\beta}^*)\right\}\right. \\
& \left. \boldsymbol{X}^\top(\widehat{\boldsymbol{\beta}}^{(k)} - \boldsymbol{\beta}^*) X_j(\widehat{\beta}_j^{(k)} - \beta_j^*)(X_j - \boldsymbol{X}_{-j}^\top \boldsymbol{w}_j^*)\right].
\end{aligned}
$$

By the proof of Thoerem 4, we can show that

$$
\begin{aligned}
& \max j E_n^{(k)}\left[\left\{\widehat{\Omega}_+^{(k)} \phi''(\boldsymbol{X}^\top \boldsymbol{\beta}^*) + \widehat{\Omega}_-^{(k)} \phi''(-\boldsymbol{X}^\top \boldsymbol{\beta}^*)\right\}\right. \\
& \left. \boldsymbol{X}^\top(\widehat{\boldsymbol{\beta}}^{(k)} - \boldsymbol{\beta}^*) X_j(\widehat{\beta}_j^{(k)} - \beta_j^*)(X_j - \boldsymbol{X}_{-j}^\top \boldsymbol{w}_j^*)\right] \\
= \; & O_p(Rs^*(\log p)^{3/2}/n).
\end{aligned}
$$

Thus, the second term is negligible due to $Rs^*(\log p)^{3/2}/\sqrt{n} \to 0$. Similarly, we can derive the second term is negligible.

Combining these results, we have that

$$
\begin{aligned}
& (n/K)^{1/2} \max_j \left|\left(\widetilde{\beta}_j^{(k)} - \beta_j^*\right) \widehat{I}_{j|-j}^{(k)} + E_n^{(k)}\left[\left\{\nabla l_\phi\left(\boldsymbol{\beta}^*; \Omega_+, \Omega_-\right)\right\}\left(X_j - \boldsymbol{X}_{-j}^\top \boldsymbol{w}_j^*\right)\right]\right| \\
= \; & (n/K)^{1/2} \max_j \left|\left(\widehat{\beta}_j^{(k)} - \beta_j^*\right)\left(\widehat{I}_{j|-j}^{(k)} - I_{j|-j}\right)\right| + o_p(1).
\end{aligned}
$$

Notice that

$$\begin{aligned}
& \left(\widehat{\beta}_j^{(k)} - \beta_j^*\right)\left(\widehat{I}_{j|-j}^{(k)} - I_{j|-j}^{(k)}\right) \\
= \; & E_n^{(k)}\left[\left\{\widehat{\Omega}_+^{(k)}\phi''(\boldsymbol{X}^\top\widehat{\boldsymbol{\beta}}^{(k)}) + \widehat{\Omega}_-^{(k)}\phi''(-\boldsymbol{X}^\top\widehat{\boldsymbol{\beta}}^{(k)})\right\} X_j(\widehat{\beta}_j^{(k)} - \beta_j^*)(X_j - \boldsymbol{X}_{-j}^\top\widehat{\boldsymbol{w}}_j^{(k)})\right] \\
& - E\left[\left\{\Omega_+\phi''(\boldsymbol{X}^\top\boldsymbol{\beta}^*) + \Omega_-\phi''(-\boldsymbol{X}^\top\boldsymbol{\beta}^*)\right\} X_j(\widehat{\beta}_j^{(k)} - \beta_j^*)(X_j - \boldsymbol{X}_{-j}^\top\boldsymbol{w}_j^*)\right].
\end{aligned}$$

By Lemma 10, 11 and the proof of Thoerem 4, we have

$$\begin{aligned}
& (n/K)^{1/2}E_n^{(k)}\left[\left\{\widehat{\Omega}_+^{(k)}\phi''(\boldsymbol{X}^\top\widehat{\boldsymbol{\beta}}^{(k)}) + \widehat{\Omega}_-^{(k)}\phi''(-\boldsymbol{X}^\top\widehat{\boldsymbol{\beta}}^{(k)})\right\} X_j(\widehat{\beta}_j^{(k)} - \beta_j^*)\right. \\
& \left.(X_j - \boldsymbol{X}_{-j}^\top\widehat{\boldsymbol{w}}_j^{(k)})\right] \\
= \; & (n/K)^{1/2}E_n^{(k)}\left[\left\{\widehat{\Omega}_+^{(k)}\phi''(\boldsymbol{X}^\top\boldsymbol{\beta}^*) + \widehat{\Omega}_-^{(k)}\phi''(-\boldsymbol{X}^\top\boldsymbol{\beta}^*)\right\} X_j(\widehat{\beta}_j^{(k)} - \beta_j^*)(X_j - \boldsymbol{X}_{-j}^\top\boldsymbol{w}_j^*)\right] \\
& + O_p(Rs^*(\log p)^{3/2}/\sqrt{n})
\end{aligned}$$

uniformly over j. Thus,

$$\begin{aligned}
& \max_j\left|\left(\widehat{\beta}_j^{(k)} - \beta_j^*\right)\left(\widehat{I}_{j|-j}^{(k)} - I_{j|-j}^{(k)}\right)\right| \\
\leq \; & \max_j\left|E_n^{(k)}\left[\left\{\widehat{\Omega}_+^{(k)}\phi''(\boldsymbol{X}^\top\boldsymbol{\beta}^{(k)}) + \widehat{\Omega}_-^{(k)}\phi''(-\boldsymbol{X}^\top\boldsymbol{\beta}^{(k)})\right\} X_j(X_j - \boldsymbol{X}_{-j}^\top\boldsymbol{w}_j^*)\right] - \right. \\
& \left. E\left[\left\{\Omega_+\phi''(\boldsymbol{X}^\top\boldsymbol{\beta}^*) + \Omega_-\phi''(-\boldsymbol{X}^\top\boldsymbol{\beta}^*)\right\} X_j(X_j - \boldsymbol{X}_{-j}^\top\boldsymbol{w}_j^*)\right]\right| \|\widehat{\boldsymbol{\beta}}^{(k)} - \boldsymbol{\beta}^*\|_2.
\end{aligned}$$

By Claim 13, we have

$$\begin{aligned}
& \max_j\left|E_n^{(k)}\left[\left\{\widehat{\Omega}_+^{(k)}\phi''(\boldsymbol{X}^\top\boldsymbol{\beta}^*) + \widehat{\Omega}_-^{(k)}\phi''(-\boldsymbol{X}^\top\boldsymbol{\beta}^*)\right\} X_j(X_j - \boldsymbol{X}_{-j}^\top\boldsymbol{w}_j^*)\right] - \right. \\
& \left. E\left[\left\{\Omega_+\phi''(\boldsymbol{X}^\top\boldsymbol{\beta}^*) + \Omega_-\phi''(-\boldsymbol{X}^\top\boldsymbol{\beta}^*)\right\} X_j(X_j - \boldsymbol{X}_{-j}^\top\boldsymbol{w}_j^*)\right]\right| \\
= \; & O_p(Rn^{-\alpha-\beta} + R(n^{-\alpha} + n^{-\beta})\sqrt{\log p/n}).
\end{aligned}$$

Thus, we have $(n/K)^{1/2}\max_j\left|\left(\widehat{\beta}_j^{(k)} - \beta_j^*\right)\left(\widehat{I}_{j|-j}^{(k)} - I_{j|-j}\right)\right| = o_p(1)$.

Next, define

$$\left(\widehat{\sigma}_j^{(k)}\right)^2 = E_n^{(k)}\left[\left\{\nabla l_\phi\left(\widehat{\boldsymbol{\beta}}^{(k)}; \widehat{\Omega}_+^{(k)}, \widehat{\Omega}_-^{(k)}\right)\right\}^2\left(X_j - \boldsymbol{X}_{-j}^\top\widehat{\boldsymbol{w}}_j^{(k)}\right)^2\right].$$

and

$$\sigma_j^2 = E\left[\left\{\nabla l_\phi\left(\boldsymbol{\beta}^*; \Omega_+, \Omega_-\right)\right\}^2\left(X_j - \boldsymbol{X}_{-j}^\top\boldsymbol{w}_j^*\right)^2\right].$$

We will show that $\max_j \left|\left(\widehat{\sigma}_j^{(k)}\right)^2 - \sigma_j^2\right| = o_p(1)$. To show this,

$$\begin{aligned}
& E_n^{(k)}\left[\left\{\nabla l_\phi\left(\widehat{\boldsymbol{\beta}}^{(k)}; \widehat{\Omega}_+^{(k)}, \widehat{\Omega}_-^{(k)}\right)\right\}^2\left(X_j - \boldsymbol{X}_{-j}^\top \widehat{\boldsymbol{w}}_j^{(k)}\right)^2\right] \\
& -E\left[\left\{\nabla l_\phi\left(\boldsymbol{\beta}^*; \Omega_+, \Omega_-\right)\right\}^2\left(X_j - \boldsymbol{X}_{-j}^\top \boldsymbol{w}_j^*\right)^2\right] \\
= \ & E_n^{(k)}\left[\left\{\nabla l_\phi\left(\widehat{\boldsymbol{\beta}}^{(k)}; \widehat{\Omega}_+^{(k)}, \widehat{\Omega}_-^{(k)}\right)\right\}^2\left\{\left(X_j - \boldsymbol{X}_{-j}^\top \widehat{\boldsymbol{w}}_j^{(k)}\right)^2 - \left(X_j - \boldsymbol{X}_{-j}^\top \boldsymbol{w}_j^*\right)^2\right\}\right] \\
& +E_n^{(k)}\left[\left\{\left\{\nabla l_\phi\left(\widehat{\boldsymbol{\beta}}^{(k)}; \widehat{\Omega}_+^{(k)}, \widehat{\Omega}_-^{(k)}\right)\right\}^2 - \left\{\nabla l_\phi\left(\boldsymbol{\beta}^*; \Omega_+, \Omega_-\right)\right\}^2\right\}\left(X_j - \boldsymbol{X}_{-j}^\top \boldsymbol{w}_j^*\right)^2\right] \\
& +\left(E_n^{(k)} - E\right)\left[\left\{\nabla l_\phi\left(\boldsymbol{\beta}^*; \Omega_+, \Omega_-\right)\right\}^2\left(X_j - \boldsymbol{X}_{-j}^\top \boldsymbol{w}_j^*\right)^2\right] \\
= \ & I_1 + I_2 + I_3.
\end{aligned}$$

For I_1, we have

$$\begin{aligned}
& E_n^{(k)}\left[\left\{\nabla l_\phi\left(\widehat{\boldsymbol{\beta}}^{(k)}; \widehat{\Omega}_+^{(k)}, \widehat{\Omega}_-^{(k)}\right)\right\}^2\left\{\left(X_j - \boldsymbol{X}_{-j}^\top \widehat{\boldsymbol{w}}_j^{(k)}\right)^2 - \left(X_j - \boldsymbol{X}_{-j}^\top \boldsymbol{w}_j^*\right)^2\right\}\right] \\
\leq \ & CE_n^{(k)}\left[\left\{\widehat{\Omega}_+^{(k)}\right\}^2\left\{\left(X_j - \boldsymbol{X}_{-j}^\top \widehat{\boldsymbol{w}}_j^{(k)}\right)^2 - \left(X_j - \boldsymbol{X}_{-j}^\top \boldsymbol{w}_j^*\right)^2\right\}\right] \\
& +CE_n^{(k)}\left[\left\{\widehat{\Omega}_-^{(k)}\right\}^2\left\{\left(X_j - \boldsymbol{X}_{-j}^\top \widehat{\boldsymbol{w}}_j^{(k)}\right)^2 - \left(X_j - \boldsymbol{X}_{-j}^\top \boldsymbol{w}_j^*\right)^2\right\}\right]
\end{aligned}$$

By Condition (C5), we have

$$\begin{aligned}
& E_n^{(k)}\left[\left\{\widehat{\Omega}_+^{(k)}\right\}^2\left\{\left(X_j - \boldsymbol{X}_{-j}^\top \widehat{\boldsymbol{w}}_j^{(k)}\right)^2 - \left(X_j - \boldsymbol{X}_{-j}^\top \boldsymbol{w}_j^*\right)^2\right\}\right] \\
= \ & E_n^{(k)}\left[\Omega_+\widehat{\Omega}_+^{(k)}\left\{\left(X_j - \boldsymbol{X}_{-j}^\top \widehat{\boldsymbol{w}}_j^{(k)}\right)^2 - \left(X_j - \boldsymbol{X}_{-j}^\top \boldsymbol{w}_j^*\right)^2\right\}\right] \\
& +(n^{-\alpha} + n^{-\beta})E_n^{(k)}\left[\widehat{\Omega}_+^{(k)}\left\{\left(X_j - \boldsymbol{X}_{-j}^\top \widehat{\boldsymbol{w}}_j^{(k)}\right)^2 - \left(X_j - \boldsymbol{X}_{-j}^\top \boldsymbol{w}_j^*\right)^2\right\}\right].
\end{aligned}$$

Notice that

$$\begin{aligned}
& \left(X_j - \boldsymbol{X}_{-j}^\top \widehat{\boldsymbol{w}}_j^{(k)}\right)^2 - \left(X_j - \boldsymbol{X}_{-j}^\top \boldsymbol{w}_j^*\right)^2 \\
= \ & \boldsymbol{X}_{-j}^\top(\widehat{\boldsymbol{w}}_j^{(k)} - \boldsymbol{w}_j^*)\left(2X_j - \boldsymbol{X}_{-j}^\top \widehat{\boldsymbol{w}}_j^{(k)} - \boldsymbol{X}_{-j}^\top \boldsymbol{w}_j^*\right) \\
= \ & -\left\{\boldsymbol{X}_{-j}^\top(\widehat{\boldsymbol{w}}_j^{(k)} - \boldsymbol{w}_j^*)\right\}^2 + 2\boldsymbol{X}_{-j}^\top(\widehat{\boldsymbol{w}}_j^{(k)} - \boldsymbol{w}_j^*)\left(X_j - \boldsymbol{X}_{-j}^\top \boldsymbol{w}_j^*\right).
\end{aligned}$$

By the condition on $Y - Q(a; \boldsymbol{X})$, $\boldsymbol{X}$ and $X_j - \boldsymbol{X}_{-j}^\top \boldsymbol{w}_j^*$, we have

$$\begin{aligned}
& \max_j E_n^{(k)}\left[\left\{\widehat{\Omega}_+^{(k)}\right\}^2\left\{\left(X_j - \boldsymbol{X}_{-j}^\top \widehat{\boldsymbol{w}}_j^{(k)}\right)^2 - \left(X_j - \boldsymbol{X}_{-j}^\top \boldsymbol{w}_j^*\right)^2\right\}\right] \\
= \ & O_p(R \log n \log(np) \max\{s^*, s'\}\sqrt{\log p/n}).
\end{aligned}$$

For I_2, we have

$$
\begin{aligned}
& I_2 \\
= \; & E_n^{(k)}\left[\left\{\nabla l_\phi\left(\widehat{\boldsymbol{\beta}}^{(k)};\widehat{\Omega}_+^{(k)},\widehat{\Omega}_-^{(k)}\right)-\nabla l_\phi\left(\boldsymbol{\beta}^*;\Omega_+,\Omega_-\right)\right\}^2\left(X_j-\boldsymbol{X}_{-j}^\top\boldsymbol{w}_j^*\right)^2\right] \\
& -2E_n^{(k)}\left[\left\{\nabla l_\phi\left(\widehat{\boldsymbol{\beta}}^{(k)};\widehat{\Omega}_+^{(k)},\widehat{\Omega}_-^{(k)}\right)-\nabla l_\phi\left(\boldsymbol{\beta}^*;\Omega_+,\Omega_-\right)\right\}\nabla l_\phi\left(\boldsymbol{\beta}^*;\Omega_+,\Omega_-\right)\right. \\
& \left.\left(X_j-\boldsymbol{X}_{-j}^\top\boldsymbol{w}_j^*\right)^2\right].
\end{aligned}
$$

Under the sub-Gaussian conditions, we have

$$
\begin{aligned}
& \max_i\left|\nabla l_\phi\left(\widehat{\boldsymbol{\beta}}^{(k)};\widehat{\Omega}_+^{(k)},\widehat{\Omega}_-^{(k)}\right)-\nabla l_\phi\left(\boldsymbol{\beta}^*;\Omega_+,\Omega_-\right)\right| \\
= \; & O_p\left((n^{-\alpha}+n^{-\beta})+R\sqrt{\log(np)}s^*\sqrt{\log p/n}\right).
\end{aligned}
$$

Thus, we have

$$
\begin{aligned}
& I_2 \\
\le \; & O_p\left((n^{-\alpha}+n^{-\beta})+R\sqrt{\log(np)}s^*\sqrt{\log p/n}\right)E_n^{(k)}\left[\left(X_j-\boldsymbol{X}_{-j}^\top\boldsymbol{w}_j^*\right)^2\right] \\
& +O_p\left((n^{-\alpha}+n^{-\beta})+R\sqrt{\log(np)}s^*\sqrt{\log p/n}\right) \\
& \times E_n^{(k)}\left[\left|\nabla l_\phi\left(\boldsymbol{\beta}^*;\Omega_+,\Omega_-\right)\right|\left(X_j-\boldsymbol{X}_{-j}^\top\boldsymbol{w}_j^*\right)^2\right].
\end{aligned}
$$

By the condition on $Y-Q(a;\boldsymbol{X})$, we have

$$
\max_i\left|\nabla l_\phi\left(\boldsymbol{\beta}^*;\Omega_+,\Omega_-\right)\right|=O_p(\sqrt{\log n}).
$$

By Lemma 14 in Loh and Wainwright (2015), we have

$$
\begin{aligned}
& \max_j\left|E_n^{(k)}\left[\left(X_j-\boldsymbol{X}_{-j}^\top\boldsymbol{w}_j^*\right)^2\right]\right| \\
= \; & \max_j E\left[\left(X_j-\boldsymbol{X}_{-j}^\top\boldsymbol{w}_j^*\right)^2\right]+O_p(\sqrt{\log p/n}).
\end{aligned}
$$

Thus, we have $\max_j I_2=o_p(1)$. For I_3, similar to I_2, we can derive that $\max_j I_3=o_p(1)$. Thus, we have

$$
\max_j\left|\left(\widehat{\sigma}_j^{(k)}\right)^2-\sigma_j^2\right|=o_p(1).
$$

Now, we show that $\min_j\sigma_j^2$ is bounded away from 0. To see this,

$$
\begin{aligned}
& \sigma_j^2 \\
\ge \; & E\left[(X_j-\boldsymbol{X}_{-j}^\top\boldsymbol{w}_j^*)^2\right]\left\{E\left[\left\{\nabla l_\phi\left(\boldsymbol{\beta}^*;\Omega_+,\Omega_-\right)\right\}^2\right]\right\}^{-1} \\
\ge \; & \lambda_{\min}\left\{E\left[\left\{\nabla l_\phi\left(\boldsymbol{\beta}^*;\Omega_+,\Omega_-\right)\right\}^2\right]\right\}^{-1}>0.
\end{aligned}
$$

Combining these results, we have

$$\begin{aligned}&(n/K)^{1/2}\max_j\Big|\left(\widetilde{\beta}_j^{(k)}-\beta_j^*\right)\widehat{I}_{j|-j}^{(k)}/\widehat{\sigma}_j^{(k)}\\&+\sigma_j^{-1}E_n^{(k)}\left[\{\nabla l_\phi\left(\boldsymbol{\beta}^*;\Omega_+,\Omega_-\right)\}\left(X_j-\boldsymbol{X}_{-j}^\top\boldsymbol{w}_j^*\right)\right]\Big|=o_p(1).\end{aligned}$$

By the Berry-Esseen bound for CLT, we have

$$\max_j\sup_{\alpha\in(0,1)}\left|P\left(\left|\left(\widetilde{\beta}_j^{(k)}-\beta_j^*\right)\widehat{I}_{j|-j}^{(k)}/\widehat{\sigma}_j^{(k)}\right|\le\Phi^{-1}(1-\alpha/2)\right)-(1-\alpha)\right|=o_p(1).$$

□

Proof of Theorem 7. Let the two split data set be $\mathcal{I}_1$ and $\mathcal{I}_2$ with sample size n_1 and n_2. We rearrange the data as the order of index in $\mathcal{I}_1$ and $\mathcal{I}_2$. Define the sigma-field generated by first j samples as $\mathcal{F}_j$.

$$\begin{aligned}\widehat{V}(\widehat{D})-V(D^*)&=(\widehat{V}-V)(\widehat{D})\\&\quad+V(\widehat{D})-V(D^*)\\&=I_1+I_2.\end{aligned}$$

For I_1,we have

$$\begin{aligned}I_1&=(E_{n_2}^{(2)}-E)\left(\widehat{W}_{\widehat{D}(\boldsymbol{X})}(Y,\boldsymbol{X},A,\widehat{\pi}_{(-2)},\widehat{Q}_{(-2)})\right)\\&\quad+E\left[\widehat{W}_{\widehat{D}(\boldsymbol{X})}(Y,\boldsymbol{X},A,\widehat{\pi}_{(-2)},\widehat{Q}_{(-2)})-W_{\widehat{D}(\boldsymbol{X})}(Y,\boldsymbol{X},A,\pi,Q)\right]\\&=(E_{n_2}^{(2)}-E)\left(\widehat{W}_{\widehat{D}(\boldsymbol{X})}(Y,\boldsymbol{X},A,\widehat{\pi}_{(-2)},\widehat{Q}_{(-2)})\right)+O_p(n_1^{-\alpha-\beta}).\end{aligned}$$

The second equality is due to Condition (C5). Let $Z_{n,i}=\widehat{W}_{\widehat{D}(\boldsymbol{X}_i)}(Y_i,\boldsymbol{X}_i,A_i,\widehat{\pi}_{(-2)},\widehat{Q}_{(-2)})-E\left[\widehat{W}_{\widehat{D}(\boldsymbol{X}_i)}(Y_i,\boldsymbol{X}_i,A_i,\widehat{\pi}_{(-2)},\widehat{Q}_{(-2)})\right]$. Consider $M_{n,i}=n_2^{-1/2}\widehat{\sigma}_V^{-1}Z_{n,i}$. We have

$$E[M_{n,i}|\mathcal{F}_{i-1}]=0$$

for any $i\in\mathcal{I}_2$. We also have that $\sum_{i\in\mathcal{I}_2}E[M_{n,i}^2|\mathcal{F}_{i-1}]=\sigma_V^2/\widehat{\sigma}_V^2\to 1$. Because Y is bounded, $\widehat{\pi}$ and $\widehat{Q}$ are consistent, and $\pi(a;\boldsymbol{X})$ is bounded away from 0 and 1, we have that $Z_{n,i}$ is bounded. Because $\widehat{\sigma}_V^2\to\sigma_V^2>0$, the conditional Linderberg condition (Condition C2) in Luedtke and Van Der Laan (2016)) holds, the martingle central limit theorem for triangular arrays [see, e.g., Theorem 2 in] shows that $n_2^{1/2}\widehat{\sigma}_V^{-1}I_1=\sum_{i\in\mathcal{I}_2}M_{n,i}\to N(0,1)$.

Next, we will show that $I_2=o_p(n_2^{-1/2})$.

$$\begin{aligned}V(\widehat{D}^*)-V(D^*)&=E\left[|\Delta|1\left\{\widehat{D}^*\ne D^*\right\}\right]\\&\le E\left[|\Delta|1\left\{|\boldsymbol{X}^\top(\widehat{\boldsymbol{\beta}}-\boldsymbol{\beta}^*)|\ge|\boldsymbol{X}^\top\boldsymbol{\beta}^*|\right\}\right]\\&\le E\left[|\Delta|1\left\{\|\widehat{\boldsymbol{\beta}}-\boldsymbol{\beta}^*\|_1\ge|\boldsymbol{X}^\top\boldsymbol{\beta}^*|\right\}\right]\end{aligned}$$

If $|\Delta|\le\psi\left(|\boldsymbol{X}^\top\boldsymbol{\beta}^*|\right)$ when $\boldsymbol{X}^\top\boldsymbol{\beta}^*$ is at a neighborhood of 0, then $V(\widehat{D}^*)-V(D^*)\lesssim\|\widehat{\boldsymbol{\beta}}-\boldsymbol{\beta}^*\|_1^{\zeta+\gamma}$. If we do not have condition, then $V(\widehat{D}^*)-V(D^*)\lesssim\|\widehat{\boldsymbol{\beta}}-\boldsymbol{\beta}^*\|_1^{\gamma}$. Under conditions, we have $I_2=o_p(n_2^{-1/2})$. This concludes the proof. □

Proof of Lemmas and Claims.

The proofs of theorems use lemmas and claims below. For the simplicity of the notation, we omit the superscript indicating split data set. For example, $\widehat{\beta}^{(k)}$ is written as $\widehat{\beta}$.

Lemma 10 *Assuming conditions in Theorem 4, we have*

$$\|\widehat{\boldsymbol{\beta}} - \boldsymbol{\beta}^*\|_1 = O_p\left(s^*(\log p/n)^{1/2}\right),$$

where $s^* = \|\boldsymbol{\beta}^*\|_0$. *Further,*

$$(\widehat{\boldsymbol{\beta}} - \boldsymbol{\beta}^*)^\top H_X(\widehat{\boldsymbol{\beta}} - \boldsymbol{\beta}^*) \lesssim s^* \log p/n,$$

and

$$\max_j(\widehat{\boldsymbol{\beta}}_{-j} - \boldsymbol{\beta}^*_{-1})^\top H_{-j}(\widehat{\boldsymbol{\beta}}_{-j} - \boldsymbol{\beta}^*_{-j}) \lesssim s^* \log p/n,$$

where $H_{\boldsymbol{X}}$ *and* H_{-j} *are defined in the proof.*

Lemma 11 *We have*

$$\max_j \|\widehat{\boldsymbol{w}}_j - \boldsymbol{w}_j^*\|_1 = O_p\left(R \max\left\{s^*, s'\right\}(\log p/n)^{1/2}\right),$$

where $s' = \max \|\boldsymbol{w}_j^*\|_0$.

The following claims are useful in the proofs of lemmas. It essentially takes advantage of the subgaussian tail of $Y_a - Q(a; \boldsymbol{X})$.

Claim 12 *Let* $Y = (Y_1, \cdots, Y_n)^T$ *be the* n *dimensional independent random vector and* $a \in \mathrm{R}^n$. *Then*

a. If Y_i*'s are bounded in* $[c, d]$ *for some* $c, d \in \mathrm{R}$.*, then for any* $t \in (0, +\infty)$

$$P\left(\left|a^\top Y - a^\top E[Y]\right| > t\right) \le 2\exp\left\{-t^2/\|a\|_2^2(d-c)^2\right\}.$$

b. If Y_i*'s are unbounded and there exists some* $M, \nu_0 \in \mathrm{R}$ *such that*

$$\max_{i=1,\cdots,n} E\left\{\exp\left[|Y_i - E(Y_i)|/M\right] - 1 - |Y_i - E(Y_i)|/M\right\} M^2 \le \nu_0/2,$$

then for any $t \in (0, +\infty)$

$$P\left(\left|a^\top Y - a^\top E(Y)\right| > t\right) \le 2\exp\left\{-t^2/2(\|a\|_2^2\nu_0 + \|a\|_\infty Mt)\right\}.$$

Proof of Claim 12. The proof is the same as Proposition 4 in Fan and Lv (2011). The results from Hoeffding's and Bernstein's inequality, respectively. □

Claim 13 *For any function vector $\boldsymbol{h}:\mathbb{R}^p\to\mathbb{R}^q$ with $\boldsymbol{h}$ is sub-Gaussian with a proxy of the order $O(R)$, $\|\boldsymbol{h}(\boldsymbol{X})\boldsymbol{X}^\top\|_{\max}\le O_p(a_n)$ and $\|E[\boldsymbol{h}(\boldsymbol{X})\boldsymbol{X}^\top]\|_\infty=O(R)$ and $\max_{j_1,j_2}E\left[h_{j_1}^4(\boldsymbol{X})\right]E\left[X_{j_2}^4\right]\le R^4$, if $a_nR^{-1}\sqrt{\log p/n}\to 0$, we have for $a=1$ or -1,*

$$\left\|E_n[(\widehat{\Omega}_a-\Omega_a)\boldsymbol{h}(\boldsymbol{X})\boldsymbol{X}^\top]\right\|_{\max}=O_p\left(Rn^{-\alpha-\beta}+R(n^{-\alpha}+n^{-\beta})\sqrt{\log p/n}\right),$$

where $\|\boldsymbol{V}\|_{\max}$ denotes the maximum of the absolute values of the entries if $\boldsymbol{V}$ is a matrix; if $\boldsymbol{V}$ is a vector, $\|\boldsymbol{V}\|_{\max}=\|\boldsymbol{V}\|_\infty$.

Proof of Claim 13. For simplicity, we will prove when $a=1$. The proof can also be applied to $a=-1$. Note that

$$\begin{aligned}&\|E_n[(\widehat{\Omega}_a-\Omega_a)\boldsymbol{h}(\boldsymbol{X})\boldsymbol{X}^\top]\|_{\max}\\ \le&\left\|E_n\left[I\{A=1\}\left(\widehat{\pi}^{-1}(1;\boldsymbol{X})-\pi^{-1}(1;\boldsymbol{X})\right)\left(\widehat{Q}(1;\boldsymbol{X})-Q(1;\boldsymbol{X})\right)\boldsymbol{h}(\boldsymbol{X})\boldsymbol{X}^\top\right]\right\|_{\max}\\ &+\left\|E_n\left[I\{A=1\}\left(\widehat{\pi}^{-1}(1;\boldsymbol{X})-\pi^{-1}(1;\boldsymbol{X})\right)(Y-Q(1;\boldsymbol{X}))\boldsymbol{h}(\boldsymbol{X})\boldsymbol{X}^\top\right]\right\|_{\max}\\ &+\left\|E_n\left[\left(I\{A=1\}\pi^{-1}(1;\boldsymbol{X})-1\right)\left(\widehat{Q}(1;\boldsymbol{X})-Q(1;\boldsymbol{X})\right)\boldsymbol{h}(\boldsymbol{X})\boldsymbol{X}^\top\right]\right\|_{\max}\\ =&I_1+I_2+I_3\end{aligned}$$

Define

$$\begin{aligned}\Omega_n(c)&=&\{\|\widehat{Q}-Q\|_\infty\le cn^{\beta}\}\cap\{\|\widehat{\pi}-\pi\|_\infty\le cn^{-\alpha}\}\cap\{\|\boldsymbol{h}(\boldsymbol{X})\boldsymbol{X}^\top\|_{\max}\le ca_n\}\\ \Theta_n&=&\{\|E_n[\boldsymbol{h}(\boldsymbol{X})\boldsymbol{h}^\top(\boldsymbol{X})]-E[\boldsymbol{h}(\boldsymbol{X})\boldsymbol{h}^\top(\boldsymbol{X})]\|_{\max}\le cR\sqrt{\log p/n}\}\\ &&\cup\{\|E_n[\boldsymbol{X}\boldsymbol{X}^\top]-E[\boldsymbol{X}\boldsymbol{X}^\top]\|_{\max}\le cR\sqrt{\log p/n}\}\end{aligned}$$

For I_1, we have

$$P(|I_1|>t)\le P(|I_1|>t\mid\Omega_n(c)\cap\Theta_n)+P(\Omega_n^c(c))+P(\Theta_n^c).$$

By Condition (C5) and sub-Gaussian conditions, we have

$$P(\Omega_n^c(c))\to 0,\quad P(\Theta_n^c)\to 0.$$

On $\Omega_n(c)\cap\Theta_n$, we have

$$\begin{aligned}|I_1|&\le&cn^{-\alpha}cn^{-\beta}\max_{j_1,j_2}E_n[|h_{j_1}(\boldsymbol{X})X_{j_2}|]\\ &\le&c^2n^{-\alpha-\beta}\max_{j_1,j_2}\left\{(E_n-E)[|h_{j_1}(\boldsymbol{X})X_{j_2}|]+E[|h_{j_1}(\boldsymbol{X})X_{j_2}|]\right\}.\end{aligned}$$

By Lemma 14 in Loh and Wainwright (2011), we have

$$P(|(E_n-E)[|h_{j_1}(\boldsymbol{X})X_{j_2}|]|\ge t)\le 6pq\exp\left\{-cn\min\left(t^2/\sigma^2,t/\sigma\right)\right\}$$

where σ is the multiplication of the proxy of X_{j_2} and $h_{j_1}(\boldsymbol{X})$. Thus, we know that

$$|I_1|\le CRn^{-\alpha-\beta}$$

on $\Omega_n(c) \cap \Theta_n$.

Now we focus on I_2. For any $t, c > 0$, we have

$$P\left(I_2 \geq t\right) \leq P\left(I_2 \geq t \mid \Omega_n(c) \cap \Theta_n\right) + P(\Omega_n^c(c)) + P(\Theta_n^c).$$

Notice that by Claim 12 and the independence of $\widehat{\pi}$. Let

$$\mathcal{E}_t = \left\{\left|E_n\left[I\{A=1\}\left(\widehat{\pi}^{-1}(1;\boldsymbol{X}) - \pi^{-1}(1;\boldsymbol{X})\right)\left(Y_1 - Q(1;\boldsymbol{X})\right)h_{j_1}(\boldsymbol{X})X_{j_2}\right]\right| > t\right\}.$$

For any $j_1 \in \{1, \cdots, q\}$ and $j_2 \in \{1, \cdots, p\}$, we have

$$\begin{aligned}
& P\left\{\mathcal{E}_t \mid A=1, \Omega_n \cap \Theta_n\right\} \\
\leq\ & 2\exp\left\{-\frac{1}{2}nt^2\left\{c^2 n^{-2\alpha}\nu_0 E_n\left[h_{j_1}^2(\boldsymbol{X})X_{j_2}^2\right] + \max_{i=1,\cdots,n}|h_{j_1}(\boldsymbol{X}_i)X_{i,j_2}|cn^{-\alpha}Mt\right\}\right\} \\
\leq\ & 2\exp\left\{-\frac{1}{2}nt^2\left\{c^2 n^{-2\alpha}\nu_0\sqrt{E_n\left[h_{j_1}^4(\boldsymbol{X})\right]E_n\left[X_{j_2}^4\right]}\right.\right. \\
& \left.\left. + \max_{i=1,\cdots,n}|h_{j_1}(\boldsymbol{X}_i)X_{i,j_2}|cn^{-\alpha}Mt\right\}\right\} \\
\leq\ & 2\exp\left\{-\frac{1}{2}nt^2\left\{c^4 n^{-2\alpha}\nu_0 \max_{j_1,j_2}\sqrt{E\left[h_{j_1}^4(\boldsymbol{X})\right]E\left[X_{j_2}^4\right]} + c^2 a_n n^{-\alpha}Mt\right\}\right\}.
\end{aligned}$$

The last inequality uses the concentration inequality for polynomials of sub-Gaussian random variables Adamczak and Wolff (2015), .i.e. there exist C,C_d, and c_1, c_2 such that

$$\begin{aligned}
P\left(|(E_n - E)[h_j^4(\boldsymbol{X})]| > t\right) &\leq 2C\exp\left\{-\frac{1}{C_D}\min\left(nt^2/(c_1R^2), n^{1/2}t^{1/2}/(c_2R^2)\right)\right\}, \\
P\left(|(E_n - E)[X_j^4]| > t\right) &\leq 2C\exp\left\{-\frac{1}{C_D}\min\left(nt^2/c_1, n^{1/2}t^{1/2}/c_2\right)\right\},
\end{aligned}$$

and the assumption that $\log p/\sqrt{n} \to 0$.

Thus, under $\log p/\sqrt{n} \to 0$, we have

$$\begin{aligned}
& P\left(I_2 \geq t \mid \Omega_n(c) \cap \Theta_n\right) \\
\leq & 2p^2\exp\left\{-nt^2/2\left\{Cc^4R^2n^{-2\alpha}\nu_0 + c^2a_nn^{-\alpha}Mt\right\}\right\}.
\end{aligned}$$

Let $t = 8(Cc^4\nu_0 + c^2M)Rc^2n^{-\alpha}\sqrt{\log p/n}$, we have

$$P\left\{I_2 \geq 8(Cc^4\nu_0 + c^2M)Rc^2n^{-\alpha}\sqrt{\log p/n} \mid \Omega_n(c) \cap \Theta_n\right\} \to 0.$$

For I_3, notice that π is bounded and apply (a) in Claim 12, we can similarly conclude $I_3 \lesssim O_p(Rn^{-\beta}\sqrt{\log pn})$. Thus, we con conclude the claim. □

Proof of Lemma 10. To simplify the notation, we omite the superscript (k)l instead, we assume that the nuisance parameter estimations used in constructing $\widehat{\Omega}_+$ and $\widehat{\Omega}_-$ are independent from the observed samples. Let S denote the support of $\boldsymbol{\beta}^*$. Denote

$$\begin{aligned}
& D(\boldsymbol{\beta}, \boldsymbol{\beta}^*) \\
=\ & E_n\left[\left\{\widehat{\Omega}_+[\phi'(\boldsymbol{X}^\top\boldsymbol{\beta}) - \phi'(\boldsymbol{X}^\top\beta^*)] - \widehat{\Omega}_-[\phi'(-\boldsymbol{X}^\top\boldsymbol{\beta}) - \phi'(-\boldsymbol{X}^\top\boldsymbol{\beta}^*)]\right\}\boldsymbol{X}^\top(\boldsymbol{\beta} - \boldsymbol{\beta}^*)\right].
\end{aligned}$$

Let $\widehat{\boldsymbol{\Delta}} = \widehat{\boldsymbol{\beta}} - \boldsymbol{\beta}^*$. Thus,

$$\begin{aligned} & D(\widehat{\boldsymbol{\beta}}, \boldsymbol{\beta}^*) \\ = \; & E_n\left[\left\{\widehat{\Omega}_+\phi'(\boldsymbol{X}^\top\widehat{\boldsymbol{\beta}}) - \widehat{\Omega}_-\phi'(-\boldsymbol{X}^\top\widehat{\boldsymbol{\beta}})\right\}\boldsymbol{X}_S^\top\widehat{\boldsymbol{\Delta}}_S\right] \\ & + E_n\left[\left\{\widehat{\Omega}_+\phi'(\boldsymbol{X}^\top\widehat{\boldsymbol{\beta}}) - \widehat{\Omega}_-\phi'(-\boldsymbol{X}^\top\widehat{\boldsymbol{\beta}})\right\}\boldsymbol{X}_S^\top\widehat{\boldsymbol{\beta}}_{\bar{S}}\right] \\ & - E_n\left[\left\{\widehat{\Omega}_+\phi'(\boldsymbol{X}^\top\boldsymbol{\beta}^*) - \widehat{\Omega}_-\phi'(-\boldsymbol{X}^\top\boldsymbol{\beta}^*)\right\}\boldsymbol{X}^\top\widehat{\boldsymbol{\Delta}}\right] \\ = \; & (I) + (II) + (III) \end{aligned}$$

By KKT condition,

$$(I) \leq \lambda_n\|\widehat{\boldsymbol{\Delta}}_S\|_1, (II) = -\lambda_n\|\widehat{\boldsymbol{\Delta}}_{\bar{S}}\|.$$

Assuming that $\widehat{W}_1$, $\widehat{W}_{-1}$, W_1 and W_{-1} are positive, we have

$$\begin{aligned} & (III) \\ = & - E_n\left[\left\{\widehat{W}_1\phi'(\boldsymbol{X}^\top\boldsymbol{\beta}^*) - \widehat{W}_{-1}\phi'(-\boldsymbol{X}^\top\boldsymbol{\beta}^*)\right\}\boldsymbol{X}^\top\widehat{\boldsymbol{\Delta}}\right] \\ = & - E_n\left[\left\{W_1\phi'(\boldsymbol{X}^\top\boldsymbol{\beta}^*) - W_{-1}\phi'(-\boldsymbol{X}^\top\boldsymbol{\beta}^*)\right\}\boldsymbol{X}^\top\widehat{\boldsymbol{\Delta}}\right] \\ & - E_n\left[\left\{[\widehat{W}_1 - W_1]\phi'(\boldsymbol{X}^\top\boldsymbol{\beta}^*) - [\widehat{W}_{-1} - W_{-1}]\phi'(-\boldsymbol{X}^\top\boldsymbol{\beta}^*)\right\}\boldsymbol{X}^\top\widehat{\boldsymbol{\Delta}}\right]. \end{aligned}$$

Due to the sub-Gaussian condition on X, following the proof of Theorem 4, we can show that

$$\begin{aligned} & P\left(\left\|E_n\left[\left\{W_1\phi'(\boldsymbol{X}^\top\boldsymbol{\beta}^*) - W_{-1}\phi'(-\boldsymbol{X}^\top\boldsymbol{\beta}^*)\right\}\boldsymbol{X}\right]\right\|_\infty \geq C\sqrt{\log p/n}\right) \\ \leq \; & c_0\exp(-c_1\log p), \end{aligned}$$

where C, c_0 and c_1 are som constants. By (the proof of) Claim 13, the second term can be bounded by $O_p\left\{[(n^{-\alpha} + n^{-\beta})(\log p/n)^{1/2} + n^{-\alpha-\beta}]\|\widehat{\Delta}\|_1\right\}$ when both model are correct. Thus, $(III) \leq C(\log p/n)^{1/2}\|\widehat{\boldsymbol{\Delta}}\|_1$ with a large enough C.

Let $\lambda_n = 2C(\log p/n)^{1/2}$, on the event,

$$\left\{\left\|E_n\left[\left\{W_1\phi'(\boldsymbol{X}^\top\boldsymbol{\beta}^*) - W_{-1}\phi'(-\boldsymbol{X}^\top\boldsymbol{\beta}^*)\right\}\boldsymbol{X}\right]\right\|_\infty \leq C\sqrt{\log p/n}\right\},$$

we have

$$D(\widehat{\boldsymbol{\beta}}, \boldsymbol{\beta}^*) \leq C(\log p/n)^{1/2}(3\|\widehat{\boldsymbol{\Delta}}_S\|_1 - \|\widehat{\boldsymbol{\Delta}}_{\bar{S}}\|_1).$$

Since ϕ is convex, we have $D(\widehat{\boldsymbol{\beta}}, \boldsymbol{\beta}^*) \geq 0$ and $\|\widehat{\boldsymbol{\Delta}}_{\bar{S}}\|_1 \leq 3\|\widehat{\boldsymbol{\Delta}}_S\|_1$. In addition, $D(\widehat{\boldsymbol{\beta}}, \boldsymbol{\beta}^*)$ can be rewritten as

$$\begin{aligned} & (\widehat{\boldsymbol{\beta}} - \boldsymbol{\beta}^*)^\top E_n[\nabla l_\phi(\widehat{\boldsymbol{\beta}}; \widehat{\Omega}_+, \widehat{\Omega}_-) - \nabla l_\phi(\boldsymbol{\beta}^*; \widehat{\Omega}_+, \widehat{\Omega}_-)X] \\ = & E_n\left\{[\widehat{\Omega}_+\phi''(\boldsymbol{X}^\top\widetilde{\boldsymbol{\beta}}) + \widehat{\Omega}_-\phi''(-\boldsymbol{X}^\top\widetilde{\boldsymbol{\beta}})](\boldsymbol{X}^\top\widehat{\boldsymbol{\Delta}})^2\right\} \\ = & E_n\left\{\left[\Omega_+\phi''(\boldsymbol{X}^\top\widetilde{\boldsymbol{\beta}}) + \Omega_-\phi''(-\boldsymbol{X}^\top\widetilde{\boldsymbol{\beta}})\right](\boldsymbol{X}^\top\widehat{\Delta})^2\right\} \\ & + E_n\left\{\left[\left(\widehat{\Omega}_+ - \Omega_+\right)\phi''(\boldsymbol{X}^\top\widetilde{\boldsymbol{\beta}}) + \left(\widehat{\Omega}_- - \Omega_-\right)\phi''(-\boldsymbol{X}^\top\widetilde{\boldsymbol{\beta}})\right](\boldsymbol{X}^\top\widehat{\boldsymbol{\Delta}})^2\right\} \end{aligned}$$

The first term satisfies the RSC condition following the Proposition 1 in Loh and Wainwright (2015) when X's are zero-mean sub-Gaussians with probablity at least $1 - c_1 \exp(-c_2 n)$. The second term has the following bound

$$\begin{aligned}
&\left| E_n \left\{ \left[\left(\widehat{\Omega}_+ - \Omega_+ \right) \phi''(\boldsymbol{X}^\top \widetilde{\boldsymbol{\beta}}) + \left(\widehat{\Omega}_- - \Omega_- \right) \phi''(-\boldsymbol{X}^\top \widetilde{\boldsymbol{\beta}}) \right] (\boldsymbol{X}^\top \widehat{\boldsymbol{\Delta}})^2 \right\} \right| \\
\leq & \|\widehat{\boldsymbol{\Delta}}\|_1^2 \left\| E_n \left[\left\{ \left(\widehat{\Omega}_+ - \Omega_+ \right) \phi''(\boldsymbol{X}^\top \widetilde{\boldsymbol{\beta}}) + \left(\widehat{\Omega}_- - \Omega_- \right) \phi''(-\boldsymbol{X}^\top \widetilde{\boldsymbol{\beta}}) \right\} \boldsymbol{X}\boldsymbol{X}^\top \right] \right\|_{\max} \\
\lesssim & (n^{-\alpha-\beta} + (n^{-\beta} + n^{-\beta}) \sqrt{\log p/n}) s^* \|\widehat{\boldsymbol{\Delta}}\|_2^2.
\end{aligned}$$

The last inequality uses the fact that $\|\widehat{\boldsymbol{\Delta}}\|_1 \leq 4\|\widehat{\boldsymbol{\Delta}}_S\|_1 \leq 4(s^*)^{1/2}\|\widehat{\boldsymbol{\Delta}}_S\|_2 \leq 4(s^*)^{1/2}\|\widehat{\boldsymbol{\Delta}}\|_2$ and the concentration inequality for the polynomial functions of independent sub-Gaussian (Adamczak and Wolff, 2015). Thus, we have $D(\widehat{\boldsymbol{\beta}}, \boldsymbol{\beta}^*) \geq \kappa\|\widehat{\boldsymbol{\Delta}}\|_2^2 - \tau\sqrt{\log p/n}\|\widehat{\boldsymbol{\Delta}}\|_1\|\widehat{\boldsymbol{\Delta}}\|_2$ on the event

$$\begin{aligned}
&\left\{ \left\| E_n \left[\left\{ W_1 \phi'(\boldsymbol{X}^\top \boldsymbol{\beta}^*) - W_{-1} \phi'(-\boldsymbol{X}^\top \boldsymbol{\beta}^*) \right\} \boldsymbol{X} \right] \right\|_\infty \leq C\sqrt{\log p/n} \right\} \cup \\
&\left\{ \|\widehat{Q} - Q\|_\infty \leq cn^{\beta} \right\} \cap \left\{ \|\widehat{\pi} - \pi\|_\infty \leq cn^{-\alpha} \right\} \cup \{\text{RSC condition holds}\}.
\end{aligned}$$

Notice that

$$\tau\sqrt{\log p/n}\|\widehat{\boldsymbol{\Delta}}\|_1\|\widehat{\boldsymbol{\Delta}}\|_2 \leq \kappa/2\|\widehat{\boldsymbol{\Delta}}\|_2^2 + \frac{\tau^2}{2\kappa}\frac{\log p}{n}\|\widehat{\boldsymbol{\Delta}}\|_1^2$$

Combining the upper bound of $D(\widehat{\boldsymbol{\beta}}, \boldsymbol{\beta}^*)$ derived above, given $\|\boldsymbol{\beta}^*\|_1 \leq \sqrt{n/\log p}$, we have

$$\kappa/2\|\widehat{\boldsymbol{\Delta}}\|_2^2 \leq C(\log p/n)^{1/2}(3\|\widehat{\boldsymbol{\Delta}}_S\|_1 - \|\widehat{\boldsymbol{\Delta}}_{\bar{S}}\|_1) + \frac{\tau^2}{2\kappa}\sqrt{\frac{\log p}{n}}\|\widehat{\boldsymbol{\Delta}}\|_1.$$

Thus, we have

$$\begin{aligned}
\|\widehat{\boldsymbol{\Delta}}\|_2 &\leq \left\{ \frac{24C}{\kappa} + \frac{4\tau^2}{\kappa^2} \right\} \sqrt{\frac{s^* \log p}{n}}, \\
\|\widehat{\boldsymbol{\Delta}}\|_1 &\leq \left\{ \frac{96C}{\kappa} + \frac{16\tau^2}{\kappa^2} \right\} s^* \sqrt{\frac{\log p}{n}}, \\
D(\widehat{\boldsymbol{\beta}}, \boldsymbol{\beta}^*) &\leq 3C \left\{ \frac{96C}{\kappa} + \frac{16\tau^2}{\kappa^2} \right\} s^* \frac{\log p}{n}.
\end{aligned}$$

Let $H_{\boldsymbol{X}} = E_n \left\{ [\widehat{\Omega}_+ \phi''(\boldsymbol{X}^\top \boldsymbol{\beta}^*) + \widehat{\Omega}_- \phi''(-\boldsymbol{X}^\top \boldsymbol{\beta}^*)](\boldsymbol{X}^\top \boldsymbol{X}) \right\}$. Note that

$$\begin{aligned}
&|D(\widehat{\beta}, \boldsymbol{\beta}^*) - (\widehat{\beta} - \boldsymbol{\beta}^*)^\top H_X(\widehat{\beta} - \boldsymbol{\beta}^*)| \\
=& \left| E_n \left[\widehat{\Omega}_+ \phi''(\boldsymbol{X}^\top \boldsymbol{\beta}) + \widehat{\Omega}_- \phi''(-\boldsymbol{X}^\top \boldsymbol{\beta}) - \widehat{\Omega}_+ \phi''(\boldsymbol{X}^\top \boldsymbol{\beta}^*) - \widehat{\Omega}_- \phi''(-\boldsymbol{X}^\top \boldsymbol{\beta}^*)(\boldsymbol{X}^\top \widehat{\boldsymbol{\Delta}})^2 \right] \right| \\
\lesssim & C\sqrt{\log(np)} s^* (\log p/n)^{1/2} \left| (\widehat{\boldsymbol{\beta}} - \boldsymbol{\beta}^*)^\top H_{\boldsymbol{X}} (\widehat{\boldsymbol{\beta}} - \boldsymbol{\beta}^*) \right|.
\end{aligned}$$

Combining with the upper bound on $D(\widehat{\boldsymbol{\beta}}, \boldsymbol{\beta}^*)$, by $s^* \log p/\sqrt{n} \to 0$, we know that

$$(\widehat{\boldsymbol{\beta}} - \boldsymbol{\beta}^*)^\top H_{\boldsymbol{X}}(\widehat{\boldsymbol{\beta}} - \boldsymbol{\beta}^*) \leq \frac{3C}{1+C}\left\{\frac{96C}{\kappa} + \frac{16\tau^2}{\kappa^2}\right\} s^* \frac{\log p}{n}.$$

Let $H_{-j} = E_n\left[\{\widehat{\Omega}_+\phi''(\boldsymbol{X}^\top\boldsymbol{\beta}^*) + \widehat{\Omega}_-\phi''(-\boldsymbol{X}^\top\boldsymbol{\beta}^*)\}\boldsymbol{X}_{-j}\boldsymbol{X}_{-j}^\top\right]$. We have

$$\begin{aligned}&(\widehat{\boldsymbol{\beta}}_{-j} - \boldsymbol{\beta}^*_{-j})^\top H_{-j}(\widehat{\boldsymbol{\beta}}_{-j} - \boldsymbol{\beta}^*_{-j})\\ \leq& 2(\widehat{\boldsymbol{\beta}} - \boldsymbol{\beta}^*)^\top H_{\boldsymbol{X}}(\widehat{\boldsymbol{\beta}} - \boldsymbol{\beta}^*)\\ &+ 2(\widehat{\beta}_j - \beta^*_j)^2 E_n\left[[\Omega_+\phi''(\boldsymbol{X}^\top\boldsymbol{\beta}^*) + \Omega_-\phi''(-\boldsymbol{X}^\top\boldsymbol{\beta}^*)]X_j^2\right].\end{aligned}$$

Thus, we have

$$\begin{aligned}&\max_j(\widehat{\boldsymbol{\beta}}_{-j} - \boldsymbol{\beta}^*_{-j})^\top H_{-j}(\widehat{\boldsymbol{\beta}}_{-j} - \boldsymbol{\beta}^*_{-j})\\ \leq\ & 2(\widehat{\boldsymbol{\beta}} - \boldsymbol{\beta}^*)^\top H_{\boldsymbol{X}}(\widehat{\boldsymbol{\beta}} - \boldsymbol{\beta}^*)\\ &+2\|\widehat{\boldsymbol{\beta}} - \boldsymbol{\beta}^*\|_2^2 \max_j E_n\left[[\widehat{\Omega}_+\phi''(\boldsymbol{X}^\top\boldsymbol{\beta}^*) + \widehat{\Omega}_-\phi''(-\boldsymbol{X}^\top\boldsymbol{\beta}^*)]X_j^2\right].\end{aligned}$$

By the sub-Gaussian assumption, we have

$$\begin{aligned}&\max_j\Big|E_n\left[[\widehat{\Omega}_+\phi''(\boldsymbol{X}^\top\boldsymbol{\beta}^*) + \widehat{\Omega}_-\phi''(-\boldsymbol{X}^\top\boldsymbol{\beta}^*)]X_j^2\right]\\ &-E\left[[\widehat{\Omega}_+\phi''(\boldsymbol{X}^\top\boldsymbol{\beta}^*) + \widehat{\Omega}_-\phi''(-\boldsymbol{X}^\top\boldsymbol{\beta}^*)]X_j^2\right]\Big| = o_p(1).\end{aligned}$$

By $\max_j E[X_j^2]$ is bounded, we have that

$$\max_j(\widehat{\boldsymbol{\beta}}_{-j} - \boldsymbol{\beta}^*_{-j})^\top H_{-j}(\widehat{\boldsymbol{\beta}}_{-j} - \boldsymbol{\beta}^*_{-j}) = O_p\left(s^*\frac{\log p}{n}\right).$$

□

Before the formal proof of Lemma 11, we establish the following claim. The following claim plays the same role as the RSC in the proof of Lemma 10. Let S' be the support of w^*.

Claim 14 *Denote $\widehat{F}(\boldsymbol{\beta}) = E_n[\widehat{U}(\boldsymbol{\beta})\boldsymbol{X}\boldsymbol{X}^\top]$, where $\widehat{U}(\boldsymbol{\beta}) = \widehat{\Omega}_+\phi''(\boldsymbol{X}^\top\boldsymbol{\beta}) + \widehat{\Omega}_-\phi''(-\boldsymbol{X}^\top\boldsymbol{\beta})$. And*

$$\kappa_D(s') = \min\left\{(s')^{1/2}(\boldsymbol{v}^\top\widehat{F}(\widehat{\boldsymbol{\beta}})\boldsymbol{v})^{1/2}/\|\boldsymbol{v}_{S'}\|_1 : \boldsymbol{v} \in R^p\backslash\{0\}, \|\boldsymbol{v}_{\bar{S}'}\|_1 \leq \xi\|\boldsymbol{v}_{S'}\|_1\right\},$$

where ξ is a positive constant. Assuming assumptions in Theorem 4, with probability tending to one, $\kappa_D(s') \geq \kappa/\sqrt{6}$.

Proof of Claim 14. By the definition of $\kappa_D(s')$ and the fact that $\|\boldsymbol{v}_{S'}\|_1 \le (s')^{1/2}\|\boldsymbol{v}_{S'}\|_2 \le (s')^{1/2}\|\boldsymbol{v}\|_2$, we only need to show that

$$\kappa_D^2(s') = \min\left\{\boldsymbol{v}^\top \widehat{F}(\widehat{\boldsymbol{\beta}})\boldsymbol{v}/\|\boldsymbol{v}\|_2^2 : \boldsymbol{v} \in \mathrm{R}^p\backslash\{0\}, \|\boldsymbol{v}_{\bar{S}'}\| \le \xi\|\boldsymbol{v}_{S'}\|_1\right\}.$$

Let $F(\boldsymbol{\beta}) = E_n[U(\boldsymbol{\beta})\boldsymbol{X}\boldsymbol{X}^\top]$, where $U(\boldsymbol{\beta}) = \Omega_+\phi''(\boldsymbol{X}^\top\boldsymbol{\beta}) + \Omega_-\phi''(-\boldsymbol{X}^\top\boldsymbol{\beta})$. We have

$$\begin{aligned}
&\boldsymbol{v}^\top \widehat{F}(\widehat{\boldsymbol{\beta}})\boldsymbol{v}/\|\boldsymbol{v}\|_2^2\\
=&\boldsymbol{v}^\top F(\boldsymbol{\beta}^*)\boldsymbol{v}/\|\boldsymbol{v}\|_2^2\\
&+ E_n\left[\left\{(\widehat{\Omega}_+ - \Omega_+)\phi''(\boldsymbol{X}^\top\boldsymbol{\beta}^*) + (\widehat{\Omega}_- - \Omega_-)\phi''(-\boldsymbol{X}^\top\boldsymbol{\beta}^*)\right\}(\boldsymbol{X}^\top\boldsymbol{v})^2/\|\boldsymbol{v}\|_2^2\right]\\
&+ E_n\left[\left\{\Omega_+(\phi''(\boldsymbol{X}^\top\widehat{\boldsymbol{\beta}}) - \phi''(\boldsymbol{X}^\top\boldsymbol{\beta}^*)) + \Omega_-(\phi''(-\boldsymbol{X}^\top\widehat{\boldsymbol{\beta}}) - \phi''(-\boldsymbol{X}^\top\boldsymbol{\beta}^*))\right\}(\boldsymbol{X}^\top\boldsymbol{v})^2/\|\boldsymbol{v}\|_2^2\right]\\
&+ E_n\left[\left\{(\widehat{\Omega}_+ - \Omega_+)(\phi''(\boldsymbol{X}^\top\widehat{\boldsymbol{\beta}}) - \phi''(\boldsymbol{X}^\top\boldsymbol{\beta}^*))\right\}(\boldsymbol{X}^\top\boldsymbol{v})^2/\|\boldsymbol{v}\|_2^2\right]\\
&+ E_n\left[\left\{(\widehat{\Omega}_- - \Omega_-)(\phi''(-\boldsymbol{X}^\top\widehat{\boldsymbol{\beta}}) - \phi''(-\boldsymbol{X}^\top\boldsymbol{\beta}^*))\right\}(\boldsymbol{X}^\top\boldsymbol{v})^2/\|\boldsymbol{v}\|_2^2\right]\\
=&\boldsymbol{v}^\top F(\boldsymbol{\beta}^*)\boldsymbol{v}/\|\boldsymbol{v}\|_2^2 + I_1 + I_2 + I_3 + I_4
\end{aligned}$$

By (the proof of) Lemma 10, I_1 can be bounded by $O_p\left(n^{-\alpha} + n^{-\beta}\right)$. For I_2, we have

$$\begin{aligned}
I_2 &\le CE_n\left[\left\{\Omega_+\phi''(\boldsymbol{X}^\top\boldsymbol{\beta}^*) + \Omega_-\phi''(-\boldsymbol{X}^\top\boldsymbol{\beta}^*)\right\}\boldsymbol{X}^\top(\widehat{\boldsymbol{\beta}} - \boldsymbol{\beta}^*)(\boldsymbol{X}^\top\boldsymbol{v})^2/\|\boldsymbol{v}\|_2^2\right]\\
&\le Cs^*\log(np)/\sqrt{n}\boldsymbol{v}^\top F(\boldsymbol{\beta}^*)\boldsymbol{v}/\|\boldsymbol{v}\|_2^2.
\end{aligned}$$

By $s^*\log(np)/\sqrt{n} \to 0$, we know that

$$I_2 \le \boldsymbol{v}^\top F(\boldsymbol{\beta}^*)\boldsymbol{v}/2\|\boldsymbol{v}\|_2^2.$$

For I_3, we have the following.

$$\begin{aligned}
I_3 \le& E_n\left[\left\{|\widehat{\Omega}_+ - \Omega_+|\phi''(\boldsymbol{X}^\top\boldsymbol{\beta}^*)|\boldsymbol{X}^\top(\widehat{\beta} - \boldsymbol{\beta}^*)|\right\}(\boldsymbol{X}_{-j}^\top v)^2/\|v\|_2^2\right]\\
\le& Cs^*\log(np)/\sqrt{n}E_n\left[\left\{|\widehat{\Omega}_+ - \Omega_+|\phi''(\boldsymbol{X}^\top\boldsymbol{\beta}^*)\right\}(\boldsymbol{X}_{-j}^\top v)^2/\|v\|_2^2\right]\\
\lesssim& (n^{-\alpha} + n^{-\beta})s^*\log(np)/\sqrt{n}.
\end{aligned}$$

Similarly, $I_4 \lesssim (n^{-\alpha} + n^{-\beta})s^*\log(np)/\sqrt{n}$. Hence, $\boldsymbol{v}^\top \widehat{F}(\widehat{\boldsymbol{\beta}})\boldsymbol{v}/\|\boldsymbol{v}\|_2^2 \ge (1/4)\boldsymbol{v}^\top F(\boldsymbol{\beta}^*)\boldsymbol{v}/\|\boldsymbol{v}\|_2^2$, with probability tending to 1. Note that

$$\begin{aligned}
\boldsymbol{v}^\top \widehat{F}(\widehat{\boldsymbol{\beta}})\boldsymbol{v}/\|\boldsymbol{v}\|_2^2 &\ge (1/4)\boldsymbol{v}^\top F(\boldsymbol{\beta}^*)\boldsymbol{v}/\|\boldsymbol{v}\|_2^2\\
&= 1/4\left\{\boldsymbol{v}^\top \boldsymbol{I}^*\boldsymbol{v}/\|\boldsymbol{v}\|_2^2 + \boldsymbol{v}^\top\left(F(\boldsymbol{\beta}^*) - \boldsymbol{I}^*\right)\boldsymbol{v}/\|\boldsymbol{v}\|_2^2\right\}\\
&\ge 1/4\left\{\lambda_{\min}\left(\boldsymbol{I}^*\right) - \left|\boldsymbol{v}^\top\left(F(\boldsymbol{\beta}^*) - \boldsymbol{I}^*\right)\boldsymbol{v}\right|/\|\boldsymbol{v}\|_2^2\right\}\\
&\ge 1/4\left\{\kappa^2 - \|\boldsymbol{v}\|_1^2\|F(\boldsymbol{\beta}^*) - \boldsymbol{I}^*\|_{\max}/\|\boldsymbol{v}\|_2^2\right\}.
\end{aligned}$$

By $\|\boldsymbol{v}\|^2 \le (\xi+1)^2\|\boldsymbol{v}_{S'}\|_1^2 \le s'(\xi+1)^2\|\boldsymbol{v}\|_2^2$, we have

$$\boldsymbol{v}^\top \widehat{F}(\widehat{\boldsymbol{\beta}})\boldsymbol{v}/\|\boldsymbol{v}\|_2^2 \ge 1/4\left\{\kappa^2 - (\xi+1)^2 s'\|F(\boldsymbol{\beta}^*) - \boldsymbol{I}^*\|_{\max}\right\}.$$

By sub-gaussian condition, we know that $\|F(\boldsymbol{\beta}^*) - \boldsymbol{I}^*\|_{\max} = O_P((\log p/n)^{1/2})$. By

$$s'(\log p/n)^{1/2} \to 0,$$

we have probability tending to 1 such that $s'\|F(\boldsymbol{\beta}^*) - \boldsymbol{I}^*\|_{\max} \le \kappa^2/[3(\xi+1)^2]$. Thus, we can conclude the claim. $\square$

Proof of Lemma 11. Let $\widehat{\Delta}_j = \widehat{\boldsymbol{w}}_j - \boldsymbol{w}_j^*$. By definition, we have

$$\begin{aligned}
&E_n\left[\left\{\widehat{\Omega}_+\phi''(\boldsymbol{X}^\top\widehat{\boldsymbol{\beta}}) + \widehat{\Omega}_-\phi''(-\boldsymbol{X}^\top\widehat{\boldsymbol{\beta}})\right\}(X_j - \boldsymbol{X}_{-j}^\top\widehat{\boldsymbol{w}}_j)^2\right] + \lambda_n'\|\widehat{\boldsymbol{w}}_j\|_1\\
\le& E_n\left[\left\{\widehat{\Omega}_+\phi''(\boldsymbol{X}^\top\widehat{\boldsymbol{\beta}}) + \widehat{\Omega}_-\phi''(-\boldsymbol{X}^\top\widehat{\boldsymbol{\beta}})\right\}(X_j - \boldsymbol{X}_{-j}^\top\boldsymbol{w}_j^*)^2\right] + \lambda_n'\|\boldsymbol{w}_j^*\|_1.
\end{aligned}$$

By rearranging terms, we have equivalently

$$\begin{aligned}
&E_n\left[\left\{\widehat{\Omega}_+\phi''(\boldsymbol{X}^\top\widehat{\boldsymbol{\beta}}) + \widehat{\Omega}_-\phi''(-\boldsymbol{X}^\top\widehat{\boldsymbol{\beta}})\right\}(\widehat{\boldsymbol{\Delta}}_j^\top\boldsymbol{X}_{-j})^2\right]\\
\le& 2E_n\left[\left\{\widehat{\Omega}_+\phi''(\boldsymbol{X}^\top\widehat{\boldsymbol{\beta}}) + \widehat{\Omega}_-\phi''(-\boldsymbol{X}^\top\widehat{\boldsymbol{\beta}})\right\}(X_j - \boldsymbol{X}_{-j}^\top\boldsymbol{w}_j^*)\widehat{\boldsymbol{\Delta}}_j^\top\boldsymbol{X}_{-j}\right]\\
&+ \lambda_n'\|\boldsymbol{w}_j^*\|_1 - \lambda_n'\|\widehat{\boldsymbol{w}}_j\|_1\\
\le& 2I_1 + \lambda_n'\|\boldsymbol{w}_j^*\|_1 - \lambda_n'\|\widehat{\boldsymbol{w}}_j\|_1.
\end{aligned}$$

Notice that

$$\lambda_n'\|\boldsymbol{w}_j^*\|_1 - \lambda_n'\|\widehat{\boldsymbol{w}}_j\|_1 = \lambda_n'\|\boldsymbol{w}_{j,S'}^*\|_1 - \lambda_n'\|\widehat{\boldsymbol{w}}_{j,S'}\|_1 - \lambda_n'\|\widehat{\boldsymbol{w}}_{j,\bar{S}'}\|_1 \le \lambda_n'\|\widehat{\boldsymbol{\Delta}}_{j,S'}\|_1 - \lambda_n'\|\widehat{\boldsymbol{\Delta}}_{j,\bar{S}'}\|_1.$$

We just need to bound I_1.

$$\begin{aligned}
&I_1\\
=&E_n\left[\left\{\Omega_+\phi''(\boldsymbol{X}^\top\boldsymbol{\beta}^*) + \Omega_-\phi''(-\boldsymbol{X}^\top\boldsymbol{\beta}^*)\right\}(X_j - \boldsymbol{X}_{-j}^\top\boldsymbol{w}^*)\widehat{\boldsymbol{\Delta}}_j^\top\boldsymbol{X}_{-j}\right]\\
&+ E_n\left[\left\{(\widehat{\Omega}_+ - \Omega_+)\phi''(\boldsymbol{X}^\top\widehat{\boldsymbol{\beta}}) + (\widehat{\Omega}_- - \Omega_-)\phi''(-\boldsymbol{X}^\top\widehat{\boldsymbol{\beta}})\right\}(X_j - \boldsymbol{X}_{-j}^\top\boldsymbol{w}_j^*)\widehat{\boldsymbol{\Delta}}_j^\top\boldsymbol{X}_{-j}\right]\\
&+ E_n\left[\left\{\Omega_+(\phi''(\boldsymbol{X}^\top\widehat{\boldsymbol{\beta}}) - \phi''(\boldsymbol{X}^\top\boldsymbol{\beta}^*)) + \Omega_-(\phi''(-\boldsymbol{X}^\top\widehat{\boldsymbol{\beta}}) - \phi''(-\boldsymbol{X}^\top\boldsymbol{\beta}^*))\right\}\right.\\
&\left.(X_j - \boldsymbol{X}_{-j}^\top\boldsymbol{w}_j^*)\widehat{\boldsymbol{\Delta}}_j^\top\boldsymbol{X}_{-j}\right]\\
=&I_{11} + I_{12} + I_{13}.
\end{aligned}$$

By the proof of Theorem 4, on the event

$$\begin{aligned}
&\left\{\max_j\left\|E_n\left[\left\{\Omega_+\phi''(\boldsymbol{X}^\top\boldsymbol{\beta}^*) + \Omega_-\phi''(-\boldsymbol{X}^\top\boldsymbol{\beta}^*)\right\}(X_j - \boldsymbol{X}_{-j}^\top\boldsymbol{w}^*)\boldsymbol{X}_{-j}\right]\right\|_\infty\right.\\
&\left.\le CR(\log p/n)^{1/2}\right\},
\end{aligned}$$

we have that

$$\begin{aligned}
|I_{11}| \le& \left\|E_n\left[\left\{\Omega_+\phi''(\boldsymbol{X}^\top\boldsymbol{\beta}^*) + \Omega_-\phi''(-\boldsymbol{X}^\top\boldsymbol{\beta}^*)\right\}(X_j - \boldsymbol{X}_{-j}^\top\boldsymbol{w}^*)\boldsymbol{X}_{-j}\right]\right\|_\infty\|\widehat{\boldsymbol{\Delta}}_j\|_1\\
\le& CR(\log p/n)^{1/2}\|\widehat{\boldsymbol{\Delta}}_j\|_1.
\end{aligned}$$

For I_{12}, we have

$$
\begin{aligned}
&|I_{12}| \\
\le &\|E_n\left[\left\{(\widehat{\Omega}_+ - \Omega_+)\phi''(\boldsymbol{X}^\top\widehat{\boldsymbol{\beta}}) + (\widehat{\Omega}_- - \Omega_-)\phi''(-\boldsymbol{X}^\top\widehat{\boldsymbol{\beta}})\right\}(X_j - \boldsymbol{X}_{-j}^\top\boldsymbol{w}_j^*)\boldsymbol{X}_{-j}\right]\|_\infty\|\widehat{\boldsymbol{\Delta}}_j\|_1.
\end{aligned}
$$

and

$$
\begin{aligned}
&\|E_n\left[\left\{(\widehat{\Omega}_+ - \Omega_+)\phi''(\boldsymbol{X}^\top\widehat{\boldsymbol{\beta}}) + (\widehat{\Omega}_- - \Omega_-)\phi''(-\boldsymbol{X}^\top\widehat{\boldsymbol{\beta}})\right\}(X_j - \boldsymbol{X}_{-j}^\top\boldsymbol{w}_j^*)\boldsymbol{X}_{-j}\right]\|_\infty \\
\le &\|E_n\left[\left\{(\widehat{\Omega}_+ - \Omega_+)\phi''(\boldsymbol{X}^\top\boldsymbol{\beta}^*) + (\widehat{\Omega}_- - \Omega_-)\phi''(-\boldsymbol{X}^\top\boldsymbol{\beta}^*)\right\}(X_j - \boldsymbol{X}_{-j}^\top\boldsymbol{w}^*)\boldsymbol{X}_{-j}\right]\|_\infty \\
&+ \|E_n\left[\left\{(\widehat{\Omega}_+ - \Omega_+)(\phi''(\boldsymbol{X}^\top\widehat{\boldsymbol{\beta}}) - \phi''(\boldsymbol{X}^\top\boldsymbol{\beta}^*))\right\}(X_j - \boldsymbol{X}_{-j}^\top\boldsymbol{w}^*)\boldsymbol{X}_{-j}\right]\|_\infty \\
&+ \|E_n\left[\left\{(\widehat{\Omega}_- - \Omega_-)(\phi''(-\boldsymbol{X}^\top\widehat{\boldsymbol{\beta}}) - \phi''(-\boldsymbol{X}^\top\boldsymbol{\beta}^*))\right\}(X_j - \boldsymbol{X}_{-j}^\top\boldsymbol{w}^*)\boldsymbol{X}_{-j}\right]\|_\infty \\
= &I_{21} + I_{22} + I_{23}.
\end{aligned}
$$

First, by (the proof of) Claim 13, $\max_j I_{21}$ can be bounded by $O_p[R(n^{-\alpha}+n^{-\beta})(\log p/n)^{1/2} + Rn^{-\alpha-\beta}]$. Second, by the sub-Gaussian of $\boldsymbol{X}$, Lemma 10, and Claim 13, we have

$$
\begin{aligned}
\max_j I_{22} \le &C\max_j\left\|E_n\left[\left\{|\widehat{\Omega}_+ - \Omega_+|\phi''(\boldsymbol{X}^\top\boldsymbol{\beta}^*)\right\}\boldsymbol{X}_{-j}|X_j - \boldsymbol{X}_{-j}^\top\boldsymbol{w}^*||\boldsymbol{X}^\top(\widehat{\boldsymbol{\beta}} - \boldsymbol{\beta}^*)|\right]\right\|_\infty \\
\le &C's^*\log p/\sqrt{n}\max_j\left\|E_n\left[\left\{|\widehat{\Omega}_+ - \Omega_+|\phi''(\boldsymbol{X}^\top\boldsymbol{\beta}^*)\right\}\boldsymbol{X}_{-j}|X_j - \boldsymbol{X}_{-j}^\top\boldsymbol{w}^*|\right]\right\|_\infty \\
\lesssim &R(n^{-\alpha} + n^{-\beta})s^*\log p/\sqrt{n}.
\end{aligned}
$$

Similarly, $\max I_{23} \lesssim R(n^{-\alpha} + n^{-\beta})s^*\log p/\sqrt{n}$. Thus,

$$
|I_{12}| \lesssim o\left(R(\log p/n)^{1/2}\|\widehat{\boldsymbol{\Delta}}_j\|_1\right)
$$

uniformly holds for all j's.

Now, we will bound I_{13}. Let $U(\boldsymbol{\beta}) = \Omega_+\phi''(\boldsymbol{X}^\top\boldsymbol{\beta}) + \Omega_+\phi''(-\boldsymbol{X}^\top\boldsymbol{\beta})$.

$$
\begin{aligned}
&|I_{13}| \\
\le &E_n\left[\left|(U(\widehat{\boldsymbol{\beta}}) - U(\boldsymbol{\beta}^*))/(U(\boldsymbol{\beta}^*)(\boldsymbol{X}^\top(\widehat{\boldsymbol{\beta}} - \boldsymbol{\beta}^*)))\right|\left|U^{1/2}(\boldsymbol{\beta}^*)(\boldsymbol{X}^\top(\widehat{\boldsymbol{\beta}} - \boldsymbol{\beta}^*))(X_j - \boldsymbol{X}_{-j}^\top\boldsymbol{w}_j^*)\right|\right. \\
&\left.\times\left|U^{1/2}(\boldsymbol{\beta}^*)\widehat{\boldsymbol{\Delta}}^\top\boldsymbol{X}_{-j}\right|\right] \\
\le &C\left(E_n[U(\boldsymbol{\beta}^*)(\boldsymbol{X}^\top(\widehat{\boldsymbol{\beta}} - \boldsymbol{\beta}^*))^2(X_j - \boldsymbol{X}_{-j}^\top\boldsymbol{w}_j^*)^2]\right)^{1/2}\left(E_n[U(\boldsymbol{\beta}^*)(\widehat{\boldsymbol{\Delta}}^\top\boldsymbol{X}_{-j})^2]\right)^{1/2} \\
= &C(\widehat{\boldsymbol{\Delta}}_j^\top F_{-j}(\boldsymbol{\beta}^*)\widehat{\boldsymbol{\Delta}}_j)^{1/2}\left(E_n[U(\boldsymbol{\beta}^*)(\boldsymbol{X}^\top(\widehat{\boldsymbol{\beta}} - \boldsymbol{\beta}^*))^2(X_j - \boldsymbol{X}_{-j}^\top\boldsymbol{w}_j^*)^2]\right)^{1/2},
\end{aligned}
$$

where $F_{-j}(\boldsymbol{\beta}) = E_n[U(\boldsymbol{\beta})\boldsymbol{X}_{-j}\boldsymbol{X}_{-j}^\top]$. To bound

$$
\max_j E_n[U(\boldsymbol{\beta}^*)(\boldsymbol{X}^\top(\widehat{\boldsymbol{\beta}} - \boldsymbol{\beta}^*))^2(X_j - \boldsymbol{X}_{-j}^\top\boldsymbol{w}_j^*)^2],
$$

we have

$$\begin{aligned}
& \max_j E_n[U(\boldsymbol{\beta}^*)(\boldsymbol{X}^\top(\widehat{\boldsymbol{\beta}}-\boldsymbol{\beta}^*))^2(X_j-\boldsymbol{X}_{-j}^\top \boldsymbol{w}_j^*)^2] \\
\leq\ & \max_j \left|(E_n-E)[U(\boldsymbol{\beta}^*)(\boldsymbol{X}^\top(\widehat{\boldsymbol{\beta}}-\boldsymbol{\beta}^*))^2(X_j-\boldsymbol{X}_{-j}^\top \boldsymbol{w}_j^*)^2]\right| \\
& +\max_j E[U(\boldsymbol{\beta}^*)(\boldsymbol{X}^\top(\widehat{\boldsymbol{\beta}}-\boldsymbol{\beta}^*))^2(X_j-\boldsymbol{X}_{-j}^\top \boldsymbol{w}_j^*)^2] \\
\leq\ & \|\widehat{\boldsymbol{\beta}}-\boldsymbol{\beta}^*\|_1^2\|(E_n-E)[U(\boldsymbol{\beta}^*)(X_j-\boldsymbol{X}_{-j}^\top \boldsymbol{w}_j^*)^2]\boldsymbol{X}\boldsymbol{X}^\top\|_{\max} \\
& +C\|\widehat{\boldsymbol{\beta}}-\boldsymbol{\beta}^*\|_2^2\max_j E[(X_j-\boldsymbol{X}_{-j}^\top \boldsymbol{w}_j^*)^2]
\end{aligned}$$

By Lemma 10, we know that there exists a constant C such that

$$|I_{13}|\leq CR(\widehat{\boldsymbol{\Delta}}_j^\top F_{-j}(\boldsymbol{\beta}^*)\widehat{\boldsymbol{\Delta}}_j)^{1/2}(s^*\log p/n)^{1/2}$$

uniformly holds for all j's.

Taking $\lambda_n'\asymp R(\log p/n)^{1/2}$ and combining bound on I_1 and $\lambda_n'\|\boldsymbol{w}_j^*\|_1-\lambda_n'\|\widehat{\boldsymbol{w}}_j\|_1$, we have

$$\begin{aligned}
& P\left(\cap_j\left\{E_n\left[\left\{\widehat{\Omega}_+\phi''(\boldsymbol{X}^\top\widehat{\boldsymbol{\beta}})+\widehat{\Omega}_-\phi''(-\boldsymbol{X}^\top\widehat{\boldsymbol{\beta}})\right\}(\widehat{\boldsymbol{\Delta}}_j^\top\boldsymbol{X}_{-j})^2\right]\right.\right. \\
& \leq CR(\widehat{\boldsymbol{\Delta}}_j^\top F_{-j}(\boldsymbol{\beta}^*)\widehat{\boldsymbol{\Delta}}_j)^{1/2}(s^*\log p/n)^{1/2} \\
& \left.\left.+3RC(\log p/n)^{1/2}\|\widehat{\boldsymbol{\Delta}}_{j,S'}\|_1-CR(\log p/n)^{1/2}\|\widehat{\boldsymbol{\Delta}}_{j,\bar{S}'}\|_1\right\}\right)\rightarrow 1.
\end{aligned}$$

Let $\widehat{F}_{-j}(\boldsymbol{\beta})=E_n[\widehat{U}(\boldsymbol{\beta})\boldsymbol{X}_{-j}\boldsymbol{X}_{-j}^\top]$, where $\widehat{U}(\boldsymbol{\beta})=\widehat{\Omega}_+\phi''(\boldsymbol{X}^\top\boldsymbol{\beta})+\widehat{\Omega}_-\phi''(-\boldsymbol{X}^\top\boldsymbol{\beta})$. To make the above inequality useful, we further link $\widehat{\boldsymbol{\Delta}}_j^\top\widehat{F}_{-j}(\widehat{\boldsymbol{\beta}})\widehat{\boldsymbol{\Delta}}_j$ with $\widehat{\boldsymbol{\Delta}}_j^\top F(\boldsymbol{\beta}^*)\widehat{\boldsymbol{\Delta}}_j$. Consider

$$\begin{aligned}
& \left|\widehat{\boldsymbol{\Delta}}_j^\top\left[\widehat{F}_{-j}(\widehat{\boldsymbol{\beta}})-F_{-j}(\boldsymbol{\beta}^*)\right]\widehat{\boldsymbol{\Delta}}_j\right| \\
=&\left|E_n\left[\left\{\widehat{\Omega}_+\phi''(\boldsymbol{X}^\top\widehat{\boldsymbol{\beta}})+\widehat{\Omega}_-\phi''(-\boldsymbol{X}^\top\widehat{\boldsymbol{\beta}})-\Omega_+\phi''(\boldsymbol{X}^\top\boldsymbol{\beta}^*)-\Omega_-\phi''(-\boldsymbol{X}^\top\boldsymbol{\beta}^*)\right\}(\widehat{\boldsymbol{\Delta}}_j^\top\boldsymbol{X}_{-j})^2\right]\right| \\
\leq&\left|E_n\left[\left\{(\widehat{\Omega}_+-\Omega_+)\phi''(\boldsymbol{X}^\top\widehat{\boldsymbol{\beta}})+(\widehat{\Omega}_--\Omega_-)\phi''(-\boldsymbol{X}^\top\widehat{\boldsymbol{\beta}})\right\}(\widehat{\boldsymbol{\Delta}}_j^\top\boldsymbol{X}_{-j})^2\right]\right| \\
&+\left|E_n\left[\left\{\Omega_+(\phi''(\boldsymbol{X}^\top\widehat{\boldsymbol{\beta}})-\phi''(\boldsymbol{X}^\top\boldsymbol{\beta}^*))+\Omega_-(\phi''(-\boldsymbol{X}^\top\widehat{\boldsymbol{\beta}})-\phi''(-\boldsymbol{X}^\top\boldsymbol{\beta}^*))\right\}(\widehat{\boldsymbol{\Delta}}_j^\top\boldsymbol{X}_{-j})^2\right]\right| \\
\leq&\left\|\widehat{\boldsymbol{\Delta}}_j\right\|_1^2\left\|E_n\left[\left\{(\widehat{\Omega}_+-\Omega_+)\phi''(\boldsymbol{X}^\top\widehat{\boldsymbol{\beta}})+(\widehat{\Omega}_--\Omega_-)\phi''(-\boldsymbol{X}^\top\widehat{\boldsymbol{\beta}})\right\}\boldsymbol{X}\boldsymbol{X}^\top\right]\right\|_{\max} \\
&+C\left|E_n\left[\left\{\Omega_+\phi''(\boldsymbol{X}^\top\boldsymbol{\beta}^*)+\Omega_-\phi''(-\boldsymbol{X}^\top\boldsymbol{\beta}^*)\right\}\boldsymbol{X}^\top(\widehat{\boldsymbol{\beta}}-\boldsymbol{\beta}^*)(\widehat{\boldsymbol{\Delta}}_j^\top\boldsymbol{X}_{-j})^2\right]\right| \\
\leq& C(n^{-\alpha}+n^{-\beta})\|\widehat{\boldsymbol{\Delta}}_j\|_1^2+C|\widehat{\boldsymbol{\Delta}}_j^\top F(\boldsymbol{\beta}^*)\widehat{\boldsymbol{\Delta}}_j|s^*\log(np)/n^{1/2}.
\end{aligned}$$

Thus, for some constant C'', we have

$$\widehat{\boldsymbol{\Delta}}_j^\top F_{-j}(\boldsymbol{\beta}^*)\widehat{\boldsymbol{\Delta}}_j\leq\left(1+C''s^*\log(np)/n^{1/2}\right)\widehat{\boldsymbol{\Delta}}_j^\top\widehat{F}_{-j}(\widehat{\boldsymbol{\beta}})\widehat{\boldsymbol{\Delta}}_j+C''(n^{-\alpha}+n^{-\beta})\|\widehat{\boldsymbol{\Delta}}_j\|_1^2,$$

uniformly hold over all j's. Combining with the inequality above, we have

$$\begin{aligned}
& \widehat{\boldsymbol{\Delta}}_j^\top \widehat{F}_{-j}(\widehat{\boldsymbol{\beta}})\widehat{\boldsymbol{\Delta}}_j \\
\leq\ & CR(\widehat{\boldsymbol{\Delta}}_j^\top F_{-j}(\boldsymbol{\beta}^*)\widehat{\boldsymbol{\Delta}}_j)^{1/2}(s^*\log p/n)^{1/2} \\
& +3CR(\log p/n)^{1/2}\|\widehat{\boldsymbol{\Delta}}_{S',j}\|_1 - CR(\log p/n)^{1/2}\|\widehat{\boldsymbol{\Delta}}_{\bar{S}',j}\|_1 \\
\leq\ & CR\sqrt{\frac{s^*\log p}{n}}\left(\left(1+C'' s^*\log(np)/n^{1/2}\right)\widehat{\boldsymbol{\Delta}}_j^\top \widehat{F}_{-j}(\widehat{\boldsymbol{\beta}})\widehat{\boldsymbol{\Delta}}_j + C''(n^{-\alpha}+n^{-\beta})\|\widehat{\boldsymbol{\Delta}}_j\|_1^2\right)^{1/2} \\
& +3CR(\log p/n)^{1/2}\|\widehat{\boldsymbol{\Delta}}_{S',j}\|_1 - CR(\log p/n)^{1/2}\|\widehat{\boldsymbol{\Delta}}_{\bar{S}',j}\|_1,
\end{aligned}$$

uniformly holds over all j's.

Notice that $(n^{-\alpha}+n^{-\beta})s^* \to 0$ and $s^*\log(np)/\sqrt{n} \to 0$, we have

$$\begin{aligned}
\widehat{\boldsymbol{\Delta}}_j^\top \widehat{F}_{-j}(\widehat{\boldsymbol{\beta}})\widehat{\boldsymbol{\Delta}}_j &\leq C'R(s^*\log p/n)^{1/2}(\widehat{\boldsymbol{\Delta}}_j^\top \widehat{F}_{-j}(\widehat{\boldsymbol{\beta}})\widehat{\boldsymbol{\Delta}}_j)^{1/2} \\
&+3CR(\log p/n)^{1/2}\|\widehat{\boldsymbol{\Delta}}_{S',j}\|_1 - CR(\log p/n)^{1/2}\|\widehat{\boldsymbol{\Delta}}_{\bar{S}',j}\|_1, \qquad (14)
\end{aligned}$$

uniformly holds over all j's with a sufficient large C' and C.

If $\left(\widehat{\boldsymbol{\Delta}}_j^\top \widehat{F}_{-j}(\widehat{\boldsymbol{\beta}})\widehat{\boldsymbol{\Delta}}_j\right)^{1/2} \leq C'R(s^*\log p/n)^{1/2}$, Inequality (14) holds trivially. If

$$\left(\widehat{\boldsymbol{\Delta}}_j^\top \widehat{F}_{-j}(\widehat{\boldsymbol{\beta}})\widehat{\boldsymbol{\Delta}}_j\right)^{1/2} > C'R(s^*\log p/n)^{1/2},$$

this implies that $3\|\widehat{\boldsymbol{\Delta}}_{S',j}\|_1 \geq \|\widehat{\boldsymbol{\Delta}}_{\bar{S}',j}\|_1$. Due to the claim with $\xi = 3$, we conclude that $\|\widehat{\boldsymbol{\Delta}}_{s',j}\|_1 \leq C(s')^{1/2}(\widehat{\boldsymbol{\Delta}}_j^\top \widehat{F}_{-j}(\widehat{\boldsymbol{\beta}})\widehat{\boldsymbol{\Delta}}_j)^{1/2}$. Combining with Inequality (14), we have $(\widehat{\boldsymbol{\Delta}}_j^\top \widehat{F}_{-j}(\widehat{\boldsymbol{\beta}})\widehat{\boldsymbol{\Delta}}_j)^{1/2} \lesssim R(\max\{s^*, s'\}\log p/n)^{1/2}$. Thus,

$$\widehat{\boldsymbol{\Delta}}_j^\top \widehat{F}_{-j}(\widehat{\boldsymbol{\beta}})\widehat{\boldsymbol{\Delta}}_j \lesssim R^2\max\{s^*, s'\}\log p/n$$

uniformly holds over all j's.

Now, we consider to bound $\|\widehat{\boldsymbol{\Delta}}_j\|_1$. First, if $6\|\widehat{\boldsymbol{\Delta}}_{S',j}\|_1 \geq \|\widehat{\boldsymbol{\Delta}}_{\bar{S}',j}\|_1$, then we have $\|\widehat{\boldsymbol{\Delta}}_j\|_1 \leq 7\|\widehat{\boldsymbol{\Delta}}_{S',j}\|_1 \lesssim (s')^{1/2}(\widehat{\boldsymbol{\Delta}}_j\widehat{F}_{-j}(\widehat{\beta})\widehat{\boldsymbol{\Delta}}_j)^{1/2}$ by claim with $\xi = 6$. Therefore, we obtain that

$$\|\widehat{\boldsymbol{\Delta}}_j\|_1 \lesssim R\max\{s^*, s'\}(\log p/n)^{1/2}.$$

Otherwise, we have $6\|\widehat{\boldsymbol{\Delta}}_{S',j}\|_1 \leq \|\widehat{\boldsymbol{\Delta}}_{\bar{S}',j}\|_1$. Then Inequality (14) implies that

$$\widehat{\boldsymbol{\Delta}}_j\widehat{F}_{-j}(\widehat{\boldsymbol{\beta}})\widehat{\boldsymbol{\Delta}}_j \leq C'R(s^*\log p/n)^{1/2}(\widehat{\boldsymbol{\Delta}}_j\widehat{F}_{-j}(\widehat{\boldsymbol{\beta}})\widehat{\boldsymbol{\Delta}}_j)^{1/2} - CR(\log p/n)^{1/2}\|\widehat{\boldsymbol{\Delta}}_{\bar{S}',j}\|_1/2.$$

Hence,

$$\|\widehat{\boldsymbol{\Delta}}_j\|_1 \leq 7/6\|\widehat{\boldsymbol{\Delta}}_{\bar{S}',j}\|_1 \lesssim R(s^*)^{1/2}(\widehat{\boldsymbol{\Delta}}_j\widehat{F}_{-j}(\widehat{\boldsymbol{\beta}})\widehat{\boldsymbol{\Delta}}_j)^{1/2} \lesssim R\max\{s^*, s'\}(\log p/n)^{1/2},$$

uniformly holds for all j's.

Because

$$\left|\widehat{\boldsymbol{\Delta}}_j^\top\left[\widehat{F}_{-j}(\widehat{\boldsymbol{\beta}}) - F(\boldsymbol{\beta}^*)\right]\widehat{\boldsymbol{\Delta}}_j\right| \lesssim (n^{-\alpha}+n^{-\beta})\|\widehat{\boldsymbol{\Delta}}_j\|_1^2 + |\widehat{\boldsymbol{\Delta}}_j^\top F_{-j}(\boldsymbol{\beta}^*)\widehat{\boldsymbol{\Delta}}_j| s^*\log p/\sqrt{n}$$

uniformly holds over all j's, we have $\widehat{\boldsymbol{\Delta}}_j^\top F_{-j}(\boldsymbol{\beta}^*)\widehat{\boldsymbol{\Delta}}_j \lesssim R\max\{s^*, s'\}\log p/n$ holds uniformly over all j's. □
